# Derivation of Diverse Hormone-Releasing Pituitary Cells from Human Pluripotent Stem Cells

**DOI:** 10.1016/j.stemcr.2016.05.005

**Published:** 2016-06-14

**Authors:** Bastian Zimmer, Jinghua Piao, Kiran Ramnarine, Mark J. Tomishima, Viviane Tabar, Lorenz Studer

**Affiliations:** 1 The Center for Stem Cell Biology, Sloan Kettering Institute for Cancer Research, 1275 York Avenue, New York, NY 10065, USA; 2 Developmental Biology Program, Sloan Kettering Institute for Cancer Research, 1275 York Avenue, New York, NY 10065, USA; 3 Department of Neurosurgery, Memorial Sloan Kettering Cancer Center, 1275 York Avenue, New York, NY 10065, USA; 4 SKI Stem Cell Research Facility, 1275 York Avenue, New York, NY 10065, USA

## Abstract

Human pluripotent stem cells (hPSCs) provide an unlimited cell source for regenerative medicine. Hormone-producing cells are particularly suitable for cell therapy, and hypopituitarism, a defect in pituitary gland function, represents a promising therapeutic target. Previous studies have derived pituitary lineages from mouse and human ESCs using 3D organoid cultures that mimic the complex events underlying pituitary gland development in vivo. Instead of relying on unknown cellular signals, we present a simple and efficient strategy to derive human pituitary lineages from hPSCs using monolayer culture conditions suitable for cell manufacturing. We demonstrate that purified placode cells can be directed into pituitary fates using defined signals. hPSC-derived pituitary cells show basal and stimulus-induced hormone release in vitro and engraftment and hormone release in vivo after transplantation into a murine model of hypopituitarism. This work lays the foundation for future cell therapy applications in patients with hypopituitarism.

## Highlights

• Defined, cGMP-ready protocol to derive anterior pituitary-lineage cells from hPSCs
• FGF8 and BMP2 patterning enables enrichment for specific hormone-producing cells
• Pituitary cells secrete multiple hormones and respond to physiological stimuli
• hPSC-pituitary cells partially rescue a rat model of hypopituitarism

## Graphical Abstract

**Figure:**
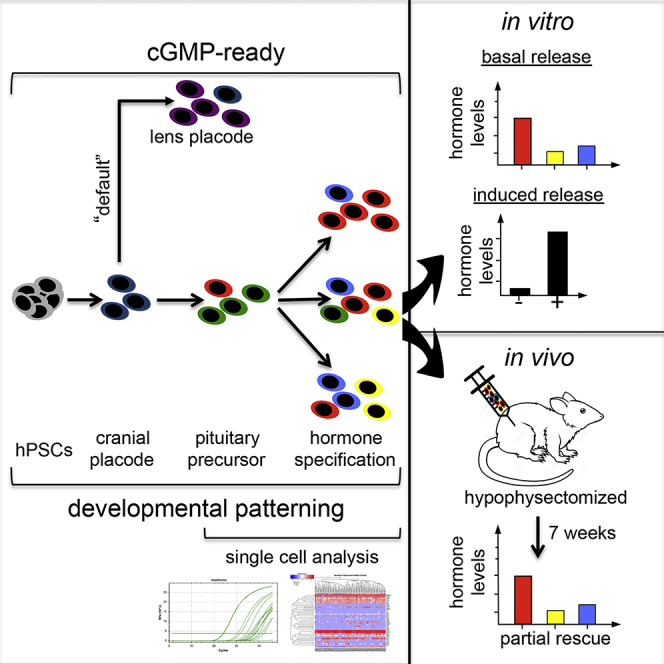

## Teaser

In this article, Studer and colleagues present the derivation of anterior pituitary cells from hPSCs under cGMP-ready monolayer conditions by mimicking in vivo developmental patterning cues. Single-cell gene-expression analysis confirms identity and diversity of in vitro generated pituitary cells. The generated cells are functional in vitro and capable of partially rescuing a rat model of hypopituitarism.

## Introduction

Human pluripotent stem cells (hPSCs) provide a unique resource for basic as well as translational research. Both human embryonic stem cells (hESCs) and human induced pluripotent stem cells (hiPSCs) are widely used to study early human development (Zhu and Huangfu, 2013), assess the toxic effects of chemicals (Dreser et al., 2015, Zimmer et al., 2012), model human diseases or cancer (Bellin et al., 2012, Funato et al., 2014, Merkle and Eggan, 2013), and discover novel potential drugs (Lee et al., 2012). Furthermore, access to greatly improved protocols for lineage-specific differentiation has led to the first experimental applications of hPSC-derived lineages in regenerative medicine such as in patients with macular degeneration (Schwartz et al., 2015). Other hPSC-based applications that are being pursued intensely include the replacement of hormone-producing cells such as in type 1 diabetes (Pagliuca et al., 2014, Rezania et al., 2014). Replacing hormone-producing cells is a particularly attractive approach for cell therapy, especially if restoration of feedback mechanisms with subsequent dynamic release of hormones can be achieved by the grafted cells.

The pituitary gland is considered the master gland of hormone function. Hypopituitarism is a disease condition with insufficient or absent function of the pituitary gland. Pituitary tumors are the most common cause but many other triggers can induce pituitary dysfunction including inborn genetic defects, brain trauma, immune and infectious diseases, or radiation therapy. The prevalence of hypopituitarism has been estimated at 46 per 100,000 (Regal et al., 2001), but this is likely an underestimation. The consequences of pituitary dysfunction are particularly serious in children where they can lead to severe learning disabilities, growth and skeletal problems, as well as effects on puberty and sexual function (Chemaitilly and Sklar, 2010). Chronic hypopituitarism requires lifelong complex hormone replacement therapies that are very costly and compromise quality of life. Furthermore, static delivery of hormones can only poorly mimic the dynamic secretion of the intact pituitary gland, which reacts to feedback mechanisms such as the hypothalamic-pituitary-adrenal (HPA) axis or the circadian clock. Therefore, there is a considerable clinical need to direct current treatment paradigms toward a more physiological and complete hormone replacement therapy (Smith, 2004).

It is conceivable that replacing the damaged cells via cell transplantation can restore pituitary function and permanently cure chronic hypopituitarism. Previous work in mouse ESCs has shown that anterior pituitary cells, capable of hormone secretion, can be generated in 3D cultures by recapitulating some of the complex morphogenetic interaction between the developing hypothalamic and oral ectoderm tissues in vitro (Suga et al., 2011). Our laboratory has recently reported a first attempt at generating functional adenohypophyseal cells from human PSCs (Dincer et al., 2013), and very recently pituitary cells have been generated from hPSCs using a 3D organoid approach (Ozone et al., 2016). While these studies represent a promising proof of concept, current protocols remain inefficient, poorly defined, and unsuitable for developing current good manufacturing practice (cGMP)-compatible culture conditions that will be eventually required for human therapeutic use.

Here, we report the efficient derivation of anterior pituitary cells from hPSCs in clinically compatible and scalable culture conditions. We further characterize the diversity of anterior pituitary subtypes achieved in vitro using single-cell mRNA expression analysis. The resulting hPSC-derived pituitary cells are functional in vitro, respond to appropriate stimuli, and are capable of secreting hormones in an animal model of hypopituitarism in vivo. Importantly, our data indicate that pituitary cell fate can be induced independent of mimicking the complex 3D organization of the developing gland. We demonstrate that by providing appropriate signals to purified placode precursor cells, pituitary identity can be specified at high efficiency, and that further manipulations of morphogen gradients enable controlled changes in the relative composition of hormonal cell types. In conclusion, we provide a robust differentiation platform to access diverse hormone-producing cell types suitable for further development toward a cell-based treatment of hypopituitarism.

## Results

### Derivation of Cranial Placode from hPSCs under Fully Defined Conditions

**Figure 1:**
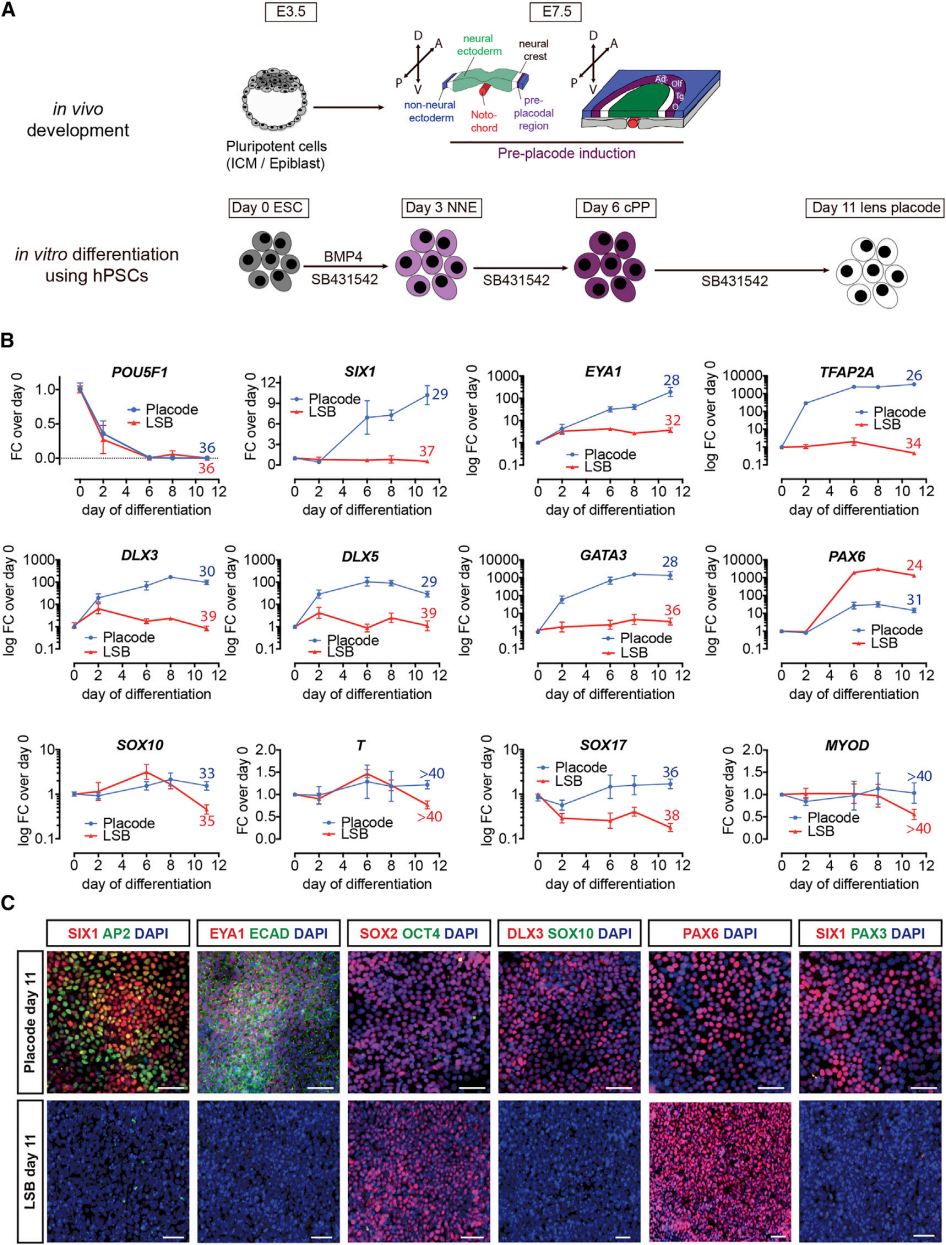
Differentiation of hPSCs into Cranial Placode Using Chemically Defined Conditions (A) Schematic representation of cranial placode in vivo development and protocol for directed differentiation of hPSCs. ICM, inner cell mass. (B) Real-time PCR gene expression time course of key cranial placode (*SIX1*, *EYA1*) and non-neural ectoderm (*TFAP2A*, *DLX3/5*, *GATA3*) genes as well as genes probing for potential contaminates (*SOX10*, *T*, *SOX17*, *MYOD*). Values are normalized to *GAPDH* and expression on day 0 of differentiation (directly before switch to differentiation medium) and plotted as mean ± SEM from four independent differentiations. Numbers above day-11 data point indicate average raw ct values for better comparison. (C) Immunofluorescence analysis comparing protein expression on day 11 of cranial placode induction protocol and LSB (LDN-193189/SB-431542; neuroectoderm). Scale bars, 50 μm. See also Figure S1.

The anterior pituitary gland is derived from cranial placode cells that form from the oral ectoderm. Therefore, the first step in establishing a defined protocol is the efficient induction of cranial placode cells competent in generating anterior pituitary lineages. The cranial placode induction protocol (PIP) presented here relies on serum-free monolayer-based induction conditions, uses fully defined cGMP-ready components, and eliminates ill-defined factors such as knockout serum replacement (KSR), Matrigel, or mouse embryonic fibroblast feeders that were part of previous protocols (Dincer et al., 2013). The specific factors used to trigger placode induction are based on signals shown to specify placode development in vivo (Figure 1A). We observed that exposure to moderate concentrations of bone morphogenetic protein 4 (BMP4) efficiently induced cranial placode cells that adopted by “default” a lens fate (Figures S1A–S1C). These results are in agreement with previous efforts of inducing placode fate from hPSCs and with data in the developing chick embryo reporting a lens default in the absence of fibroblast growth factor (FGF) signals (Bailey et al., 2006, Leung et al., 2013). Temporal expression analysis revealed the rapid loss of pluripotency markers and induction of key placode genes such as *SIX1*, *EYA1*, and *DLX3/5* after 6 days of differentiation at both mRNA (Figure 1B) and protein (Figure 1C) levels. Transcripts of contaminating cell types such as *SOX10* (neural crest), *SOX17* (endoderm), *T/Brachyury* (mesoderm), or *MYOD* (myogenic lineages) were not induced under these conditions (Figure 1B). Our previous study reported PAX3^+^ trigeminal placode as the “default” identity of hPSC-derived placode cells (Dincer et al., 2013). Our current method, using the defined placode induction conditions, shows PAX6 rather than PAX3 expression (Figure 1C). To further quantify the yield and selectivity of placode induction and PAX6 expression, we used hESC genetic reporter lines for *SIX1*::*H2B-GFP*, *PAX6*::*H2B-GFP*, *SOX10*::*GFP* (Chambers et al., 2012, Mica et al., 2013). Flow analysis confirmed robust induction of PAX6 and SIX1 without SOX10, consistent with anterior cranial placode in the absence of contaminating neural crest cells (Figures S1D and S1E).

### Patterning of Anterior Pituitary from hPSC-Derived Cranial Placode

**Figure 2:**
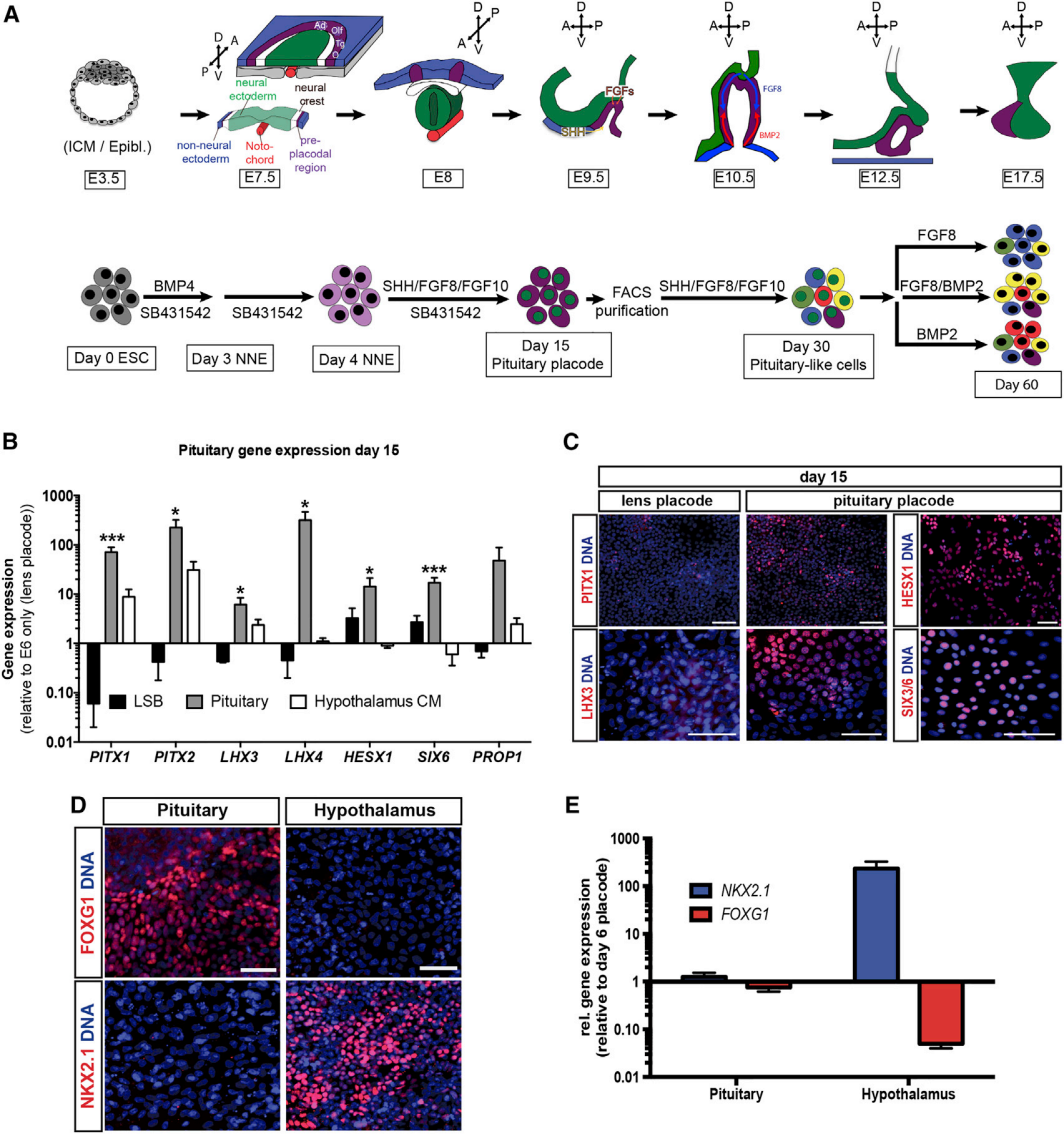
Pituitary Specification of Anterior Cranial Placode-Derived hPSCs (A) Schematic representation of pituitary gland in vivo development and protocol for directed differentiation of hPSCs into anterior pituitary-like cells. ICM/Epibl., inner cell mass/epiblast; NNE, non-neural ectoderm. (B) Real-time PCR analysis comparing expression of key genes involved in pituitary development in LSB, pituitary condition, and medium conditioned by hypothalamic neuroectoderm (Hypothalamus CM) after 15 days of differentiation in the respective medium. Values are normalized to *GAPDH* and gene expression on day 15 of lens differentiation (E6 only) and plotted as mean ± SEM of at least four independent experiments. ^∗^p < 0.05, ^∗∗∗^p < 0.001 compared with E6 only condition on day 15. (C) Immunofluorescence analysis comparing expression of PITX1 and LHX3 after 15 days of differentiation under lens or pituitary conditions as well as expression of HESX1 and SIX3/6 on day 15 of pituitary differentiation. Scale bars, 50 μm. (D) Immunofluorescence comparison of cells differentiated for 15 days under either pituitary or hypothalamus condition. Cells were stained for either FOXG1 (Pituitary) or NKX2.1 (Hypothalamus). Scale bars, 50 μm. (E) qRT-PCR analysis of day 0-15 cells differentiated under pituitary or hypothalamic ectoderm condition probing for *NKX2.1* and *FOXG1*. Values have been normalized to *GAPDH* and expression in day-6 placode cells and are plotted as means ± SEM of two to four independent experiments. See also Figures S2 and S3.

The complex morphogenetic development of the pituitary gland occurs during early embryonic stages (∼embryonic day 10 [E10] in mouse and between embryonic weeks 4 and 8 in human). Both the anterior and intermediate lobes of the pituitary gland are derived from oral ectoderm, which corresponds to the pituitary placode while the posterior pituitary gland develops from the neural ectoderm (Figure 2A). Inductive tissue interactions as well as various defined signaling pathways including FGFs, BMPs, and Sonic hedgehog (SHH) are thought to be important for proper gland development and hormonal subtype specifications in vivo (Zhu et al., 2007) (Figure 2A, upper panel). Here we assessed the ability of these developmental pathways to induce pituitary identity (Figure 2A, lower panel). Our data show that timed exposure to SHH, FGF8, and FGF10 robustly induces gene expression associated with anterior pituitary development including *PITX1/2*, *LHX3/4*, *HESX1*, and *SIX6* (Figure 2B). Where possible, we confirmed expression at the protein level using antibodies against PITX1, LHX3, HESX1, and SIX6 (Figure 2C). We compared our cGMP-ready E8/E6-based induction protocol with our published KSR-based PIP (Dincer et al., 2013). To compensate for KSR lot-to-lot variability, which can dramatically affect differentiation efficiency, we performed PIP using two distinct concentrations of the BMP inhibitor LDN-193189. After 15 days of differentiation, the cells were analyzed using qRT-PCR probing for pan-placodal markers such as *SIX1* as well as the pan-pituitary markers *PITX1*, *PITX2*, *LHX3*, and *LHX4*. In addition, the neuroectoderm marker *PAX6* and the non-neural ectoderm transcription factor *TFAP2A* were included in the analysis (Figure S2A). Cell identity was further confirmed at the protein level using immunofluorescence staining for SIX1 and LHX3 (Figure S2B). The KSR lot used for these experiments failed to effectively induce pituitary or placode identity as shown by the low expression of *SIX1* and *TFAP2A* and high expression of *PAX6*. Lowering the LDN-193189 concentration was able to partially but not fully rescue that effect compared with our new E8/E6-based protocol. The cGMP-ready protocol presented here works reliably and with comparable efficiency across various hESC and hiPSC lines (Figures S3A–S3C). Furthermore, we confirmed that the recombinant protein SHH can be replaced by small-molecule smoothened agonists such as purmorphamine and SAG (Figures S3D and S3E). However, despite robust induction of anterior pituitary-lineage markers, we observed an increase in cell death when using the small-molecule-based induction conditions, which prompted us to use recombinant SHH for subsequent studies.

**Figure 3:**
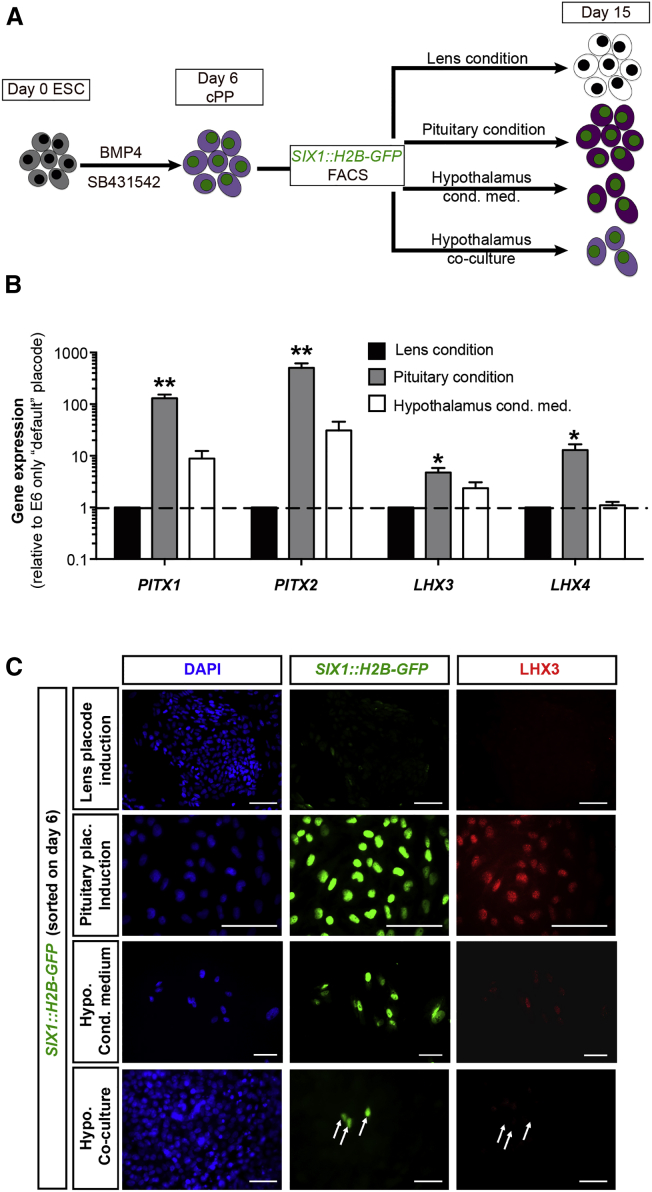
Pituitary Placode Induction from Purified SIX1:GFP-H2B Cells (A) Schematic representation of the experimental outline. hESC were differentiated under default conditions for 6 days. Unpatterned SIX1^+^ cells were FACS purified and cultured for an additional 9 days in various conditions. Cells were analyzed on day 15. (B) Gene-expression analysis of key pituitary genes in cells grown in three conditions described in (A). Values are normalized to *GAPDH* and gene expression on day 15 of lens differentiation (E6 only) and plotted as mean ± SEM of at least four independent experiments. ^∗^p < 0.05, ^∗∗^p < 0.01 compared with E6-only condition on day 15. (C) Immunofluorescence analysis of SIX1 sorted cells after 9 days of differentiation in respective medium condition. Arrows indicate absence of LHX3 expression in SIX1^+^ cells in co-culture condition. Scale bars, 50 μm.

Interestingly, medium conditioned by hPSC-derived hypothalamic anlage (Maroof et al., 2013, Merkle et al., 2015) (Figures 2D and 2E) was not sufficient to robustly induce pituitary marker expression (such as *LHX4*, *HESX1*, and *SIX6*; Figure 2B). *LHX4* and *SIX6* have been implicated in pituitary progenitor expansion, which might explain the reduced cell yield using hypothalamic-conditioned medium. Despite compelling evidence to the contrary (Suga et al., 2011), our data suggest that defined extrinsic cues might be sufficient to induce pituitary placode identity. To directly assess the role of hypothalamic tissue during pituitary placode induction and differentiation, we made use of the *SIX1*::*H2B-GFP* reporter cell line. Early placode cells differentiated under default conditions were sorted at day 6 of differentiation for *SIX1*::*H2B-GFP* expression followed by further differentiation under either lens conditions (default), in the presence of SHH, FGF8, and FGF10 (pituitary conditions) or in the presence of medium conditioned by hPSC-derived hypothalamic neuroectoderm (Figure 3A). Gene-expression analysis revealed that even purified *SIX1*::*H2B-GFP*^*+*^ cells, devoid of any hypothalamic lineage cells, are capable of expressing all key pituitary markers in response to defined cues. In contrast, hypothalamic conditioned medium failed to induce *LHX4* expression above levels observed under default lens conditions (Figure 3B). The levels of induction, especially *LHX4*, were slightly lower in the *SIX1*::*H2B-GFP* purified cells compared with our standard pituitary PIP. This is possibly due to the relatively late pituitary induction, which started on day 6 (versus day 4) to exclude patterning of the pre-placode tissue. Alternatively, the sorting process or the associated cell dissociation may have also contributed to the slight decrease in induction efficiency.

In a separate set of experiments, we co-cultured day-6 sorted, *SIX1*::*H2B-GFP*^*+*^ cells in direct contact with hPSC-derived hypothalamic anlage and stained the cells for the pan-placodal marker SIX1 and the pituitary marker LHX3 after 9 days of additional co-culture (day 15). As additional controls, we included the “default” lens conditions as well as our standard pituitary conditions and hypothalamic-conditioned medium (Figure 3A). While the lens condition formed lentoid-like clusters and downregulated SIX1 expression, the pituitary condition resulted in SIX1/LHX3 double-positive cells. The conditioned medium maintained SIX1 while inducing only low levels of LHX3. In the co-culture, SIX1 expression was maintained but no LHX3 expression was detected (Figure 3C). These findings support the idea that exposure to defined extrinsic cues is sufficient to direct hPSC into pituitary lineages cells. While we cannot rule out that further optimization of co-culture conditions may yield pituitary-lineage cells, we demonstrate that defined signals present a robust alternative that during cell manufacturing should greatly reduce variability inherent to complex co-culture systems.

### hPSC-Derived Adenohypophyseal Cells Are Functional

**Figure 4:**
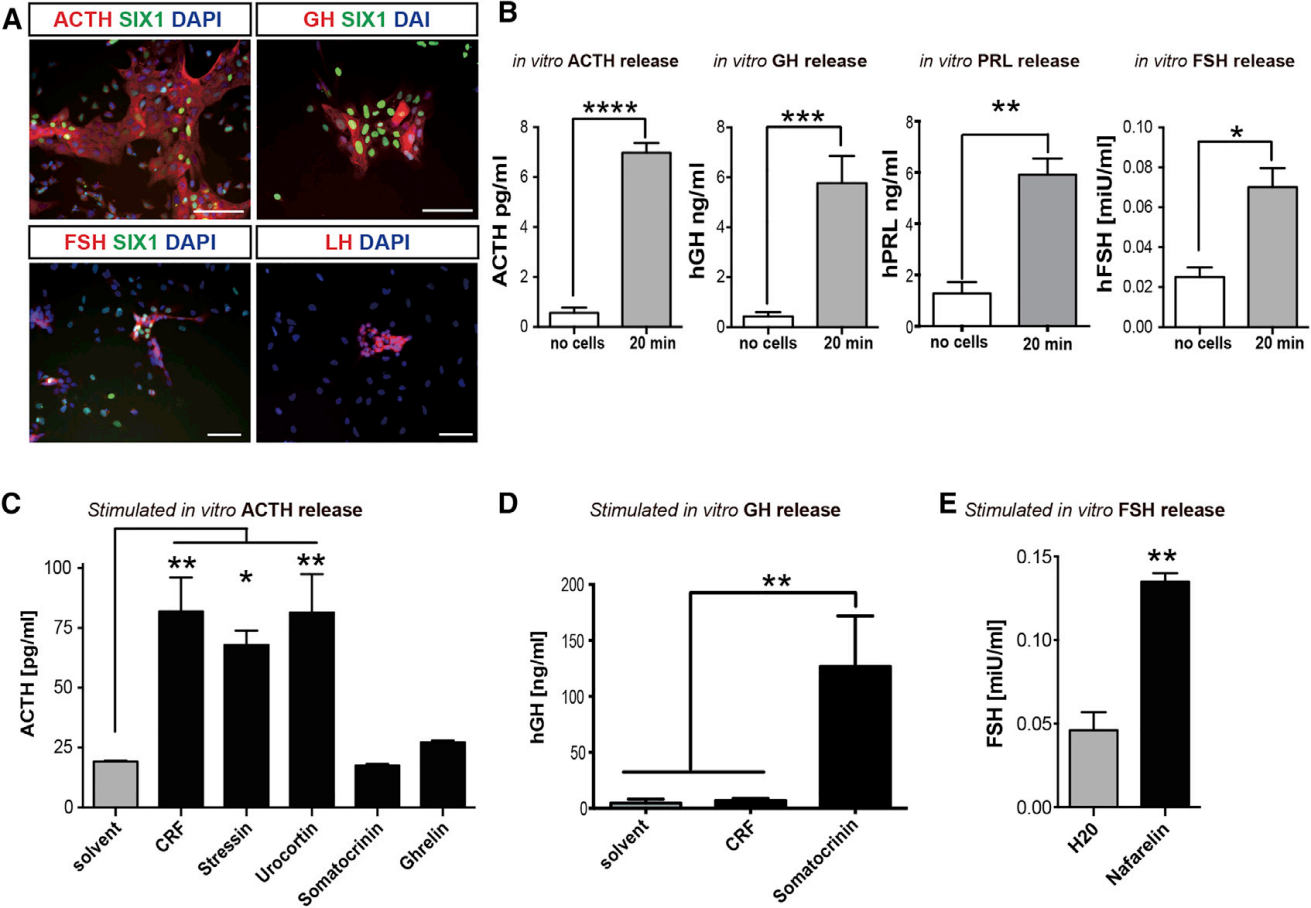
Functional Characterization of Anterior Pituitary Cells (A) Immunofluorescence analysis of anterior pituitary cells after 30 days of differentiation. On day 30 the culture contains corticotrophs (ACTH), somatotrophs (GH), and gonadotrophs (FSH, LH). Scale bar, 50 μm. (B) In vitro basal hormone release on day 30 of differentiation as assessed by ELISA. Data are plotted as mean ± SEM of three independent experiments. ^∗^p < 0.05, ^∗∗^p < 0.01, ^∗∗∗^p < 0.001, ^∗∗∗∗^p < 0.0001 compared with no cells (differentiation medium only). (C–E) Quantification of hormone levels after 24 hr of in vitro stimulation using compounds triggering hormone release. ACTH release was specifically induced by CRF, stressin, or urocortin and not by somatocrinin or ghrelin (C), GH release was induced by somatocrinin but not CRF (D), and FSH release was induced by nafarelin (E). Data are plotted as mean ± SEM of three independent experiments. ^∗^p < 0.05, ^∗∗^p < 0.01 compared with the solvent control.

The main function of the anterior pituitary (adenohypophysis) is to secrete six specific hormones controlling key events in the human body including stress response (adrenocorticotropic hormone [ACTH]), skeletal growth (growth hormone [GH]), metabolism (thyroid-stimulating hormone [TSH]), and reproductive functions (prolactin [PRL], follicle-stimulating hormone [FSH], luteinizing hormone [LH]). We therefore assessed the presence of hormonal subtypes in our culture. We were able to detect ACTH, GH, and PRL as well as FSH- and LH-expressing cells in our culture at day 30 of differentiation (Figure 4A). ELISA measurements of cell-culture supernatant confirmed basal secretion rates for ACTH, GH, and FSH (Figure 4B). Hormone release in the anterior pituitary gland is tightly regulated by several feedback mechanisms from the various target organs as well as from upstream hypothalamic signals delivered via the hypophyseal portal venous plexus. Therefore, the functional response of pituitary cells must integrate multiple, distinct regulatory stimuli. hPSC-derived pituitary cells at day 30 of differentiation showed induction of ACTH release in response to stimulation with corticotropin-releasing factor (CRF), stressin, or urocortin. In contrast, exposure to inappropriate stimuli such as ghrelin or somatocrinin did not trigger ACTH release (Figure 4C). On the other hand, somatocrinin but not CRF exposure triggered a significant increase in GH release (Figure 4D). Finally, the cultures also induced FSH release upon exposure to nafarelin (Figure 4E).

### Single-Cell Gene-Expression Analysis Reveals Diversity of Hormonal Lineages

**Figure 5:**
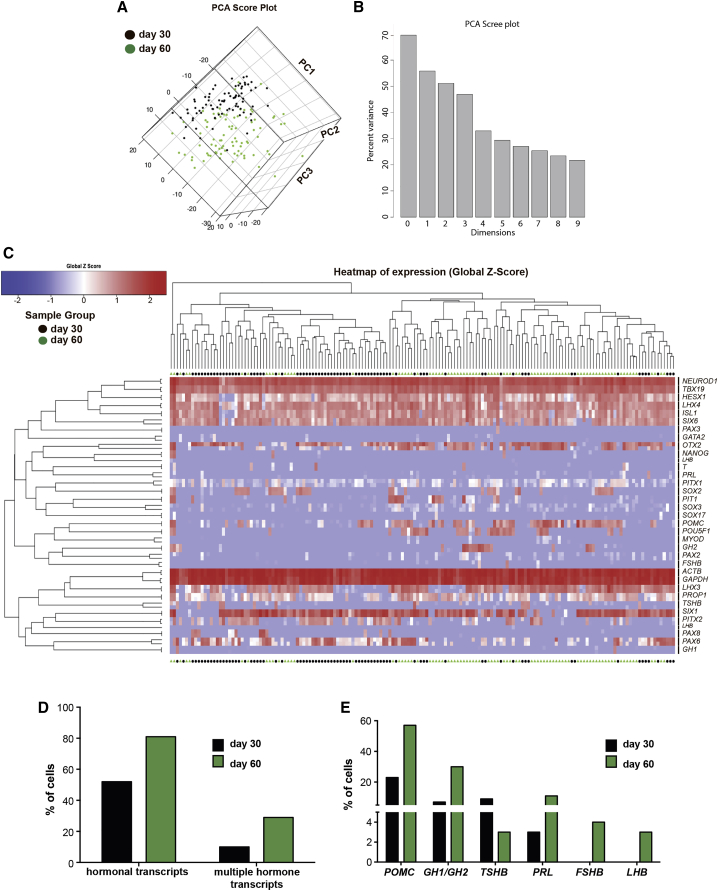
Temporal Single-Cell qRT-PCR Analysis of Anterior Pituitary Development In Vitro (A and B) Principal component analysis of single cells on day 30 (black) and day 60 (green) of differentiation reveals two distinct populations of cells. (C) Unsupervised hierarchical clustering of day-30 and day-60 cells using 34 different primer pairs identifies two clusters of cells with very few leading cells (day-30 cells resembling day-60 cells) and cells lagging behind (day-60 cells still more closely resembling day-30 cells). (D) Quantification of hormone-expressing cells on day 30 and day 60 as well as percentage of cells expressing more than one hormonal transcript per cell. (E) Expression of individual hormonal transcripts per single cell on day 30 and day 60, respectively. See also Figures S4 and S5.

Differentiation of the hormonal progenitor lineages within the adenohypophysis (Tabar, 2011) is a tightly regulated spatial and temporal process involving various patterning events (Figure 2A). To address the diversity of progenitor fates in our hPSC-based culture system, we performed single-cell qRT-PCR analyses. We probed for 34 genes spanning pituitary development from undifferentiated PSCs to mature hormone-producing cells. We also included primers to survey for potential mesodermal or endodermal contaminants such as *T*/*Brachyury*, *MYOD*, and *SOX17*. Principal component analysis (PCA) of cells at day 30 and day 60 of differentiation showed a clear time-dependent change in expression with only few cells moving ahead of schedule (i.e., day-30 cells showing a day-60 profile) or being delayed (i.e., day-60 cells retaining a day-30 signature) (Figure 5A). The scree plot (Figure 5B) defined the PCA components that explain most of the variability of the data. Hierarchical clustering confirmed the separation of cells largely along the time axis, resulting in two main clusters interspersed with several smaller subclusters (Figure 5C). In addition, heatmaps based on the raw ct values are provided in Figure S4. We further validated our single-cell data by immunofluorescence staining in day-30 cultures for the progenitor marker HESX1, and for NEUROD1, a more mature marker transiently expressed in corticotrophs. Immunofluorescence analysis at day 15 of differentiation served as negative control for NEUROD1 (Figure S5A). We confirmed co-labeling of HESX1 and NEUROD1 in the same cell at day 30 of differentiation. However, the levels of HESX1 expression were much lower at day 30 compared with day 15.

Our analysis revealed that day-30 cultures contain an already high percentage of pituitary-like cells with ∼70% of cells co-expressing pituitary transcripts such as *PITX1* and *LHX3*. Only four cells (∼5% of all cells analyzed) expressed *T*, *SOX17*, or *MYOD*, suggesting a low percentage of contaminating cells. Most cells expressed *TBX19* (*TPIT*) a transcription factor shown to be crucial for the development of the *POMC* lineage (Lamolet et al., 2001). Furthermore, most cells expressed the pan-placodal marker *SIX1* and co-expressed *PAX6* compatible with pituitary placode fate. However, we also observed expression of other placode fates including *PAX2* (epibranchial), *PAX3* (trigeminal), or *PAX8* (otic) that together were detected in about 20% of the *SIX1*^+^ population.

The ultimate functional units of the anterior pituitary are cells that secrete specific hormones. Our single-cell analysis showed that at day 30 of differentiation approximately 50% of the cells expressed at least one hormonal mRNA species. This percentage increased to about 80% by day 60, indicating further in vitro maturation (Figure 5D). This increase in the percentage of hormone-positive cells was one of the main factors responsible for the overall difference observed between day-30 and day-60 cells. There have been reports suggesting that both the developing and adult rodent pituitary gland contain cells that express more than a single hormone (Nunez et al., 2003, Villalobos et al., 2004). Indeed, in our hPSC-derived cultures we could detect expression of more than one hormonal transcript (hereafter termed “multiple hormone transcripts”) in 10% of the cells by day 30 of differentiation. By day 60 of differentiation, this percentage increased to ∼30% of the total cell population (Figure 5D). We found that the majority of multiple hormone transcript-expressing cells by day 60 expressed both *POMC* and *GH* (∼10%). Cells expressing more than two hormone transcript types were only detected by day 60 and always included *POMC* expression (Figure S5B).

The most frequent hormonal transcript expressed in hPSC-derived pituitary cells at day 30 of differentiation was *POMC* (30% of total cells), thought to emerge from the dorsal pituitary anlage. The more ventral cell types such as *GH* or *TSH* made up about 20% of the total cell population by day 30 of differentiation. *PRL* was expressed in an even smaller subset of cells. Finally, *FSH* and *LH*, the two most ventral cell types, which appear only at later stages of development, were not detected by day 30 (Figure 5E). At day 60 of differentiation the number of *POMC*- and *GH*-expressing cells increased to 55% and 30%, respectively. Only few cells expressed *FSH* (∼4%) and *LH* (∼3%), even at day 60 of differentiation (Figure 5E). In addition to the single-cell PCR we characterized the cell-surface marker expression of the day-30 culture using the commercially available BD Lyoplate screening kit (Figure S6).

### Dorsal-Ventral Patterning of Anterior Pituitary Cells In Vitro Using Patterning Factors

**Figure 6:**
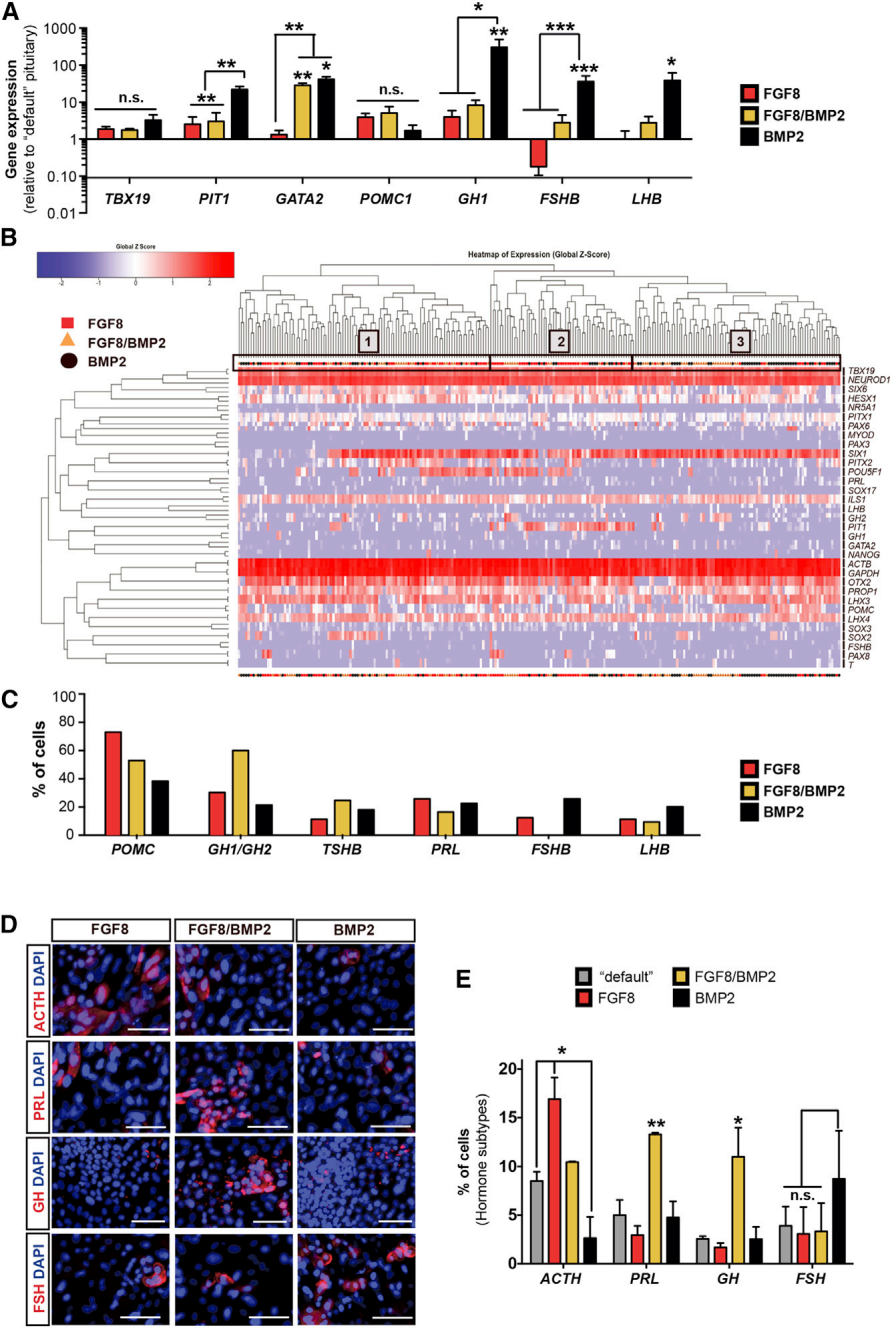
Specification of Hormonal Cells of the Pituitary In Vitro (A) Bulk qRT-PCR analysis of day-60 cells patterned with FGF8, FGF8/BMP2, or BMP2 for 30 days. Patterning with BMP2 induced a more ventral cell identity (*PIT1*, *GATA2*, *GH1*, *FSHB*, and *LHB*) while FGF8 suppressed ventral cell types (FSHB). Data are plotted as mean ± SEM of two to four independent experiments. ^∗^p < 0.05, ^∗∗^p < 0.01, ^∗∗∗^p < 0.001 (n.s., not significant) compared with the “default” pituitary differentiation on day 60. (B) Unsupervised hierarchical clustering of FGF8, FGF8/BMP2, and BMP2 patterned cells using 34 primer pairs identified three larger clusters of cells, with cluster 2 mainly comprising cells patterned by FGF8 (or FGF8/BMP2) and cluster 3 mainly comprising cells patterned by BMP2 (or FGF8/BMP2). (C) Quantification of hormonal transcripts per cell in different patterning conditions. Data are plotted as percentage of cells expressing the respective transcript (ct < 35 cycles in combination with a proper melting curve). (D) Immunofluorescence analysis (representative images) of hormone expression in cells patterned with FGF8, FGF8/BMP2, or BMP2 on day 60 of differentiation. Scale bars, 50 μm. (E) Quantification of hormone-expressing cells (per subtype) in different patterning conditions on day 60 of differentiation. High levels of FGF8 induced dorsal fate (ACTH) while intermediate levels of FGF8 and BMP2 induced dorsal/ventral fates (PRL and GH) compared with the default condition (E6 only). Data are plotted as mean ± SEM of two independent experiments. ^∗^p < 0.05, ^∗∗^p < 0.01 (n.s., not significant) compared with the “default” (E6 only) pituitary differentiation on day 60. See also Figure S4.

Hypopituitarism is a very diverse and complex disease. Depending on the cause of pituitary dysfunction, the type of hormones affected can vary. For example, GH deficits are commonly observed in patients with inborn genetic disease (van Gelderen and van der Hoog, 1981) but can also occur in patients following radiation treatment (Sklar and Constine, 1995). In contrast, lymphocytic hypophysitis, an autoimmune disease of the pituitary gland, affects primarily ACTH (Rivera, 2006). Therefore, for the broad application of hPSC-derived pituitary cells in the future, cell replacement therapy may need to be customized to the specific needs of a given patient population. Since our standard conditions mostly yield dorsal, ACTH^+^ cells, we asked whether additional signals can be used to enhance the production of more ventral cell types. It has been shown that FGF8 and BMP2 signaling gradients play an important role in dorsal-ventral patterning of the mouse pituitary gland (Rosenfeld et al., 2000) (Figure 2A). We therefore treated pituitary-lineage cells with high concentrations of either FGF8 (dorsalizing) or BMP2 (ventralizing), or with a mixture of the two patterning factors at intermediate concentrations to mimic morphogen gradients occurring in vivo. Gene-expression studies for key transcription factors of pituitary precursor lineage and hormonal subtypes confirmed the need for BMP2 to generate the most ventral cell types. *FSHB* and *LHB* were significantly upregulated in the presence of BMP2 while FGF8 exerted a negative effect on *FSHB* yield (Figure 6A).

We next performed single-cell qRT-PCR analysis to increase the resolution of our analysis. Unsupervised hierarchical clustering of single cells at day 60, treated with FGF8, BMP2, or a combination of both factors, revealed three larger clusters of cells (Figure 6B). In addition, heatmaps based on the raw ct values are provided in Figure S4. Cells corresponding to each of the three treatments were observed in every cluster. However, 49% of all the total cells in cluster 3 were from the BMP2-treated group while 56% of all the cells in cluster 2 were derived from the FGF8 group. Cluster 1 represented cells from all three treatments in roughly equal proportions. By analyzing single-cell expression for each of the anterior pituitary hormones, we confirmed findings predicted from mouse in vivo studies (Rosenfeld et al., 2000) in our hPSC-based culture system. High concentrations of FGF8 led to increased numbers of *POMC*^+^ cells compared with high concentrations of BMP2 (75% versus 40%, respectively) (Figure 6C). Furthermore, intermediate concentrations of both signaling molecules resulted in an increase of cells expressing *GH* and *TSHB* compared with either FGF8 or BMP2 alone (Figure 6C). Finally, high concentrations of BMP2 decreased the number of *POMC*^+^ dorsal cell types and increased the number of ventral *FSHB* and *LHB* cell types.

Immunofluorescence confirmed the single-cell qRT-PCR data for four hormones under the various dorsal-ventral patterning conditions (FGF8, FGF8/BMP2, BMP2; Figure 6D). Quantification showed a bias toward dorsal ACTH (*POMC*)-expressing cells in the FGF8 treated culture. PRL and GH were the most abundant hormones observed upon patterning with FGF8/BMP2 while FSH was the most abundant cell type in BMP2-treated cultures (Figure 6E). POMC is the precursor polypeptide of ACTH and 44 amino acids are removed during translation, which ultimately gives rise to the hormone ACTH.

### Grafted Human ESC-Derived Anterior Pituitary Cells Function in a Model of Hypopituitarism

**Figure 7:**
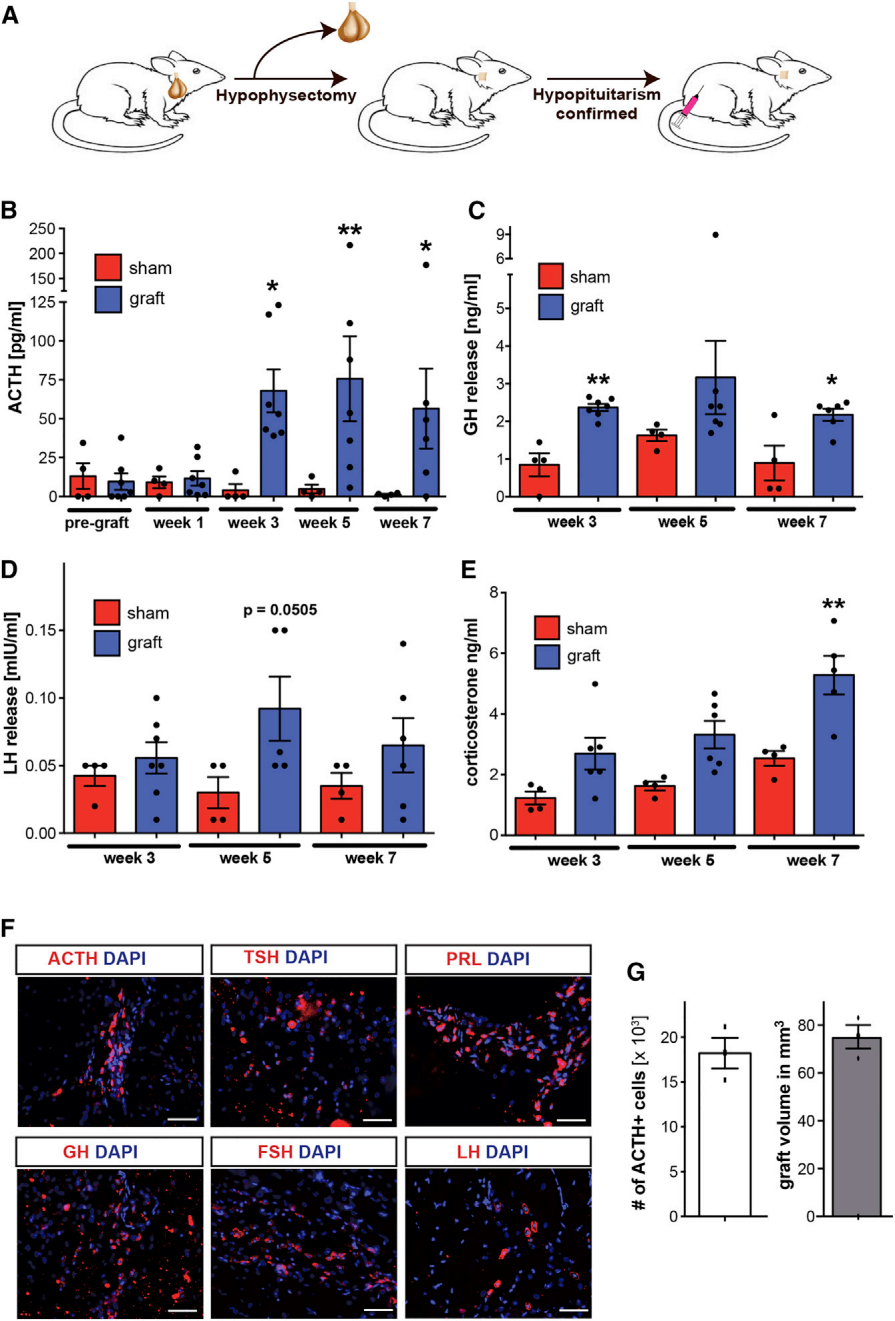
In Vivo Survival and Function of hPSC-Derived Anterior Pituitary Cells (A) Schematic representation of experimental layout. After surgical removal of the pituitary gland and confirmation of hypopituitarism, cells embedded in Matrigel were transplanted subcutaneously. (B–E) ACTH (B), GH (C), LH (D), and corticosterone (E) levels were quantified in plasma for up to 7 weeks after transplantation of the cells using ELISA. Data are plotted as mean ± SEM with each dot representing an individual animal. ^∗^p < 0.05, ^∗∗^p < 0.01 compared with the corresponding sham control. (F) Immunohistological analysis of grafts 7 weeks after transplantation. Cells of each of the six hormonal lineages of the anterior pituitary gland were detectable within the graft. Scale bars, 50 μm. (G) Quantification of cells expressing ACTH and the corresponding graft volume 7 weeks after transplantation. Data are plotted as mean ± SEM with each dot representing an individual animal (three animals in total). See also Figure S7.

To assess the ability of hPSC-derived pituitary cells to survive and function in vivo, we transplanted day-30 cells into hypophysectomized rats. After surgical removal of the pituitary gland (Figure 7A) using a parapharyngeal approach, hypopituitarism in the rats was confirmed by measuring ACTH levels in the plasma via ELISA. Rats that were successfully hypophysectomized were divided into two experimental groups, the sham group (n = 4) receiving Matrigel-only injections and the grafting group (n = 7) receiving Matrigel containing hESC-derived pituitary cells (day 30, standard conditions without BMP2 treatment). Following transplantation of 2 × 10^6^ cells subcutaneously, both treatment and control groups were followed for 7 weeks post-transplantation and pituitary hormone levels in the plasma were monitored (Figures 7B–7D). At 3 weeks after transplantation, hormone levels in the grafted group increased compared with the 1-week time point while the levels in the sham group remained largely unchanged. ACTH levels remained at higher levels in the grafted versus control group for the 7-week period of the experiment (Figure 7B). Increases in the level of two other hormones, namely GH and LH, were more variable and did not reach significance at all the time points tested. However, significant increases in GH levels were detected at 3 and 7 weeks after transplantation (Figure 7C). No significant increase in LH levels was observed (Figure 7D). Hormone levels in grafted animals were compared with levels in intact age-matched rats and found to be ∼40% for ACTH, ∼28% for GH, and ∼20% for PRL (Figure S7). In a final step to evaluate the function of the transplanted cells, we performed measurements of the target hormones affected by pituitary hormone secretion. The normal HPA-axis response involves an increase in glucocorticoids secreted by the adrenal glands upon release of ACTH (Webster and Sternberg, 2004). In humans, ACTH triggers the release of cortisol, whereas the main glucocorticoid in rodents is corticosterone (Wand, 2008). We therefore measured corticosterone levels in both experimental groups (Figure 7E). The grafted group showed consistently higher levels of corticosterone than sham-grafted animals resulting in a statistically significant difference by 7 weeks after transplantation. These data indicate an appropriate response by the host adrenal glands to human ACTH released by grafted hPSC-derived cells. At 7 weeks after transplantation, the animals were euthanized and the graft was analyzed histologically. We were able to detect cells expressing each of the six anterior pituitary hormones within the grafts (Figures 7F and 7G), confirming cell survival and suggesting, at least in part, further in vivo differentiation and maturation of the cells. Stereological quantification of the grafts showed an average of 3.08 × 10^6^ ± 0.42 × 10^6^ (average ± SD; n = 3) human cells per graft, with the majority (>95%) having a placode identity, as determined by co-expression of SIX1 and human nuclear antigen (hNA) by immunohistochemistry. The proportion of Ki67-positive proliferating cells in the graft was 9.6% ± 0.6% (average ± SD; n = 3) of the hNA^+^ population. The entire graft stained negative for the pluripotency-associated surface markers SSEA-4 and Tra1-60. No signs of tumors were detected up to 7 weeks after transplantation. Stereological analyses showed an average of 18,212 ± 2,969 ACTH^+^ cells per animal (optical fractionator method), and a graft volume of 81 ± 10 mm^3^ (Cavalieri estimator; average ± SD, n = 3).

## Discussion

A goal of our study was the development of a defined and highly efficient protocol to generate anterior pituitary lineages that obviates the need for co-culture or complex media formulations and that should be suitable for clinical-grade cell manufacturing. Using fluorescence-activated cell sorting (FACS)-purified *SIX1*::*H2B-GFP*^+^ placode cells, we demonstrate that pituitary fate can be induced at the expense of the default lens fate upon exposure to SHH, FGF8, and FGF10. Under these conditions, induction occurs in the absence of any hypothalamic cells previously thought to be critical for pituitary specification. While the signals essential for pituitary cell-fate induction from hPSCs do not require the presence of hypothalamic lineage cells, the interaction with the hypothalamic anlage is likely important for the complex tissue movements characteristic of pituitary gland development such as the formation of the Rathke's pouch. In future studies it may be interesting to combine directed differentiation and organoid culture techniques to study developmental tissue interactions and to retain 3D cytoarchitecture. However, our current strategy offers obvious advantages for cell manufacturing of highly defined, therapeutically relevant cell types at scale. The cGMP-ready PIP presented here has several major advantages over the previously published protocol (Dincer et al., 2013). It is well established that KSR can exhibit considerable lot-to-lot variability (Rao, 2008, Schwartz et al., 2011). The protocol presented here overcomes these challenges and yields improved overall differentiation efficiencies under conditions of minimal lot-to-lot variability based on testing >10 different lots of Essential8 for hiPSC maintenance and >5 different lots of Essential6 for differentiation.

Our study made use of single-cell transcriptional profiling to define the heterogeneity of hormone-producing cells derived from hPSCs. The technology is a powerful tool that allowed us to determine the percentage of various hormone-producing cells and shifts in this distribution upon treatment with patterning factors or comparing different time points in vitro. However, one of the challenges of single-cell profiling is the largely binary readout of the expression data (McDavid et al., 2013). The HESX1/NEUROD1 validation experiment exemplifies this drawback, as the single-cell RT-PCR was not able to capture the decrease in HESX1 expression over time as observed by immunocytochemistry. Therefore, our binary approach of identifying expressed genes leads to the possibility that lowly expressed genes may be read as positive despite their expression levels not being high enough to achieve meaningful translation into protein. For example, we observed some instances of “inappropriate” expression patterns such as the co-expression of *SIX1* with *PAX6* and *PAX3* (though at different expression levels), which would indicate a mixed regional cranial placode identity. Similarly, the presence of putative plurihormonal cells was based purely on the expression of multiple hormonal transcripts. The presence of plurihormonal cells in the pituitary gland has been reported previously (Nunez et al., 2003, Villalobos et al., 2004) and is a well-known feature in pituitary adenomas (Scheithauer et al., 1986). However, it will be important to further assess in hPSC-derived pituitary cells whether transcripts for multiple hormones indeed result in the expression and secretion of multiple hormones from individual cells. To resolve some of the limitations of the single-cell gene-expression study, it will be important in the future to obtain appropriate positive and negative controls in order to define tissue-relevant thresholds of expression for each transcript. Unfortunately, developmentally matched human tissues are not readily available. In the present study we used immunofluorescence analysis as an independent method to quantify bona fide hormone-producing cells. Due to the lower sensitivity of this assay compared with qRT-PCR analysis, the percentages of hormone-positive cells were found to be lower but with the same relative proportions of the various hormone-producing cells.

We demonstrate that exposure to FGF8 and BMP2 can bias the dorsal-ventral composition of hormone-producing cells from ACTH^+^ cells to FSH^+^ or LH^+^ cells. It should be noted that in vivo, cell types expressing different hormones are not necessarily located in specific areas but are scattered throughout the mature gland (Ericson et al., 1998, Olson et al., 2006). In addition, some reports argue for a less important role of extrinsic signaling compared with intrinsic factors in pituitary cell specification (Davis et al., 2011). Our human in vitro system provides a valuable tool to answer such questions. The current differentiation protocols only achieve partial enrichment of specific hormonal lineages. Therefore, lineage selection via cell sorting or the development of more sophisticated patterning strategies will be required to obtain purified populations such as GH^+^ cells suitable for treating patients with selective GH deficiency (Bianchi et al., 2008). Our cell-surface epitope screen presents candidate markers that may be suitable for isolating specific hormone lineages. One cell type lacking in the current in vitro culture system are TSH^+^ cells. Studies in sheep suggest that proper hypothalamic input is required for the development of thyrotrophs (Szarek et al., 2008). Therefore, it is conceivable that hypothalamic lineage cells may still be required for generating TSH^+^ fates. Alternatively, TSH^+^ cells may simply require further optimization of our defined induction conditions or longer in vitro differentiation periods.

The in vivo studies show clear evidence of survival and hormone release up to 7 weeks after transplantation. Whether the lower percentage of hormone-producing cells in vivo is due to limited cell survival or increased proliferation of immature pituitary progenitors remains to be determined. Future studies also need to define the optimal transplantation time point for in vivo yield and hormone subtype and demonstrate the long-term survival of hPSC-derived pituitary cells. One key question is whether the functional integration of human cells will eventually require orthotopic placement of the graft into the pituitary gland or hypothalamic region. Preliminary experiments with orthotopic (median eminence) placement of the cells into lesioned animals resulted in low cell survival, suggesting the need for further optimization of such a transplantation paradigm. Ectopic placement in accessible locations does offer advantages from a translational perspective, namely the ability to access and remove the graft, should adverse side effects develop. However, hypothalamic release factors are the main triggers for hormonal release in the anterior pituitary gland (Smith and Vale, 2006). These release hormones have a short half-life and do not persist in the circulation. They are released into the hypophyseal portal venous plexus, which ensures immediate local delivery to the gland. Thus it is likely that the ectopically placed hPSC-derived grafts did not gain significant exposure to hypothalamic input. Our data therefore suggest a degree of autonomous hormonal release by the grafted cells, although a more comprehensive assessment of integration into homeostatic, endocrine mechanisms will be necessary. Based on existing literature on grafting primary cells (Maxwell et al., 1998, Naik et al., 1997), it is likely that proper regulation of at least some of the pituitary hormones will require orthotopic placement. Direct access to the skull base and pituitary gland in the mouse requires aggressive surgical procedures, such as transaural approaches or parapharyngeal neck dissection, complicating such studies. This is in marked contrast to human patients in whom surgical access to the pituitary gland can be safely performed through minimally invasive endoscopic transnasal routes (Tabar, 2011).

In conclusion, our study offers systematic access to human pituitary cell development and represents a powerful tool to obtain diverse hormone-producing cell types on demand and at scale. The platform also presents an important proof of concept in establishing functional and engraftable hormone-producing cells for regenerative medicine under conditions suitable for preclinical and clinical development.

## Experimental Procedures

### Human Pluripotent Stem Cell Culture and Differentiation

The four hPSC lines (three hESC, one hiPSC) used in this study were maintained under feeder-free conditions using Essential8 and VTN-N. Cells were passaged twice a week using EDTA. All the differentiations carried out in this study are described in detail in Supplemental Experimental Procedures.

### Phenotypic Characterization of Differentiated Cells

hPSC-derived cell types were characterized using immunofluorescence, qRT-PCR, flow cytometry, high content surface marker phenotyping (BD Lyoplate), and single hormone as well as multiplex ELISA. Details, including all antibodies used in this study, are described in Supplemental Experimental Procedures.

### Single-Cell qRT-PCR

FACS-sorted single cells were captured using the C1 System (Fluidigm). Single-cell qRT-PCR was performed using wet-lab tested DELTAgene Assays (Fluidigm) in combination with EvaGreen chemistry using a BioMark System (Fluidigm). A complete list of primers used for this study is provided in Supplemental Experimental Procedures.

### Animal Maintenance

The Animal Care and Use Committee at Memorial Sloan Kettering Cancer Center approved all animal protocols. All procedures were performed in accordance with NIH guidelines.

### Statistical Analysis

All data are presented as mean ± SEM. The number of independent biological experiments performed for each analysis is indicated in the respective figure legend. Unpaired t test or one-way ANOVA with Bonferroni multiple-comparison post hoc test was used to assess significance levels. Significance levels were set at ^∗^p < 0.05, ^∗∗^p < 0.01, ^∗∗∗^p < 0.001, and ^∗∗∗^p < 0.0001.

## Author Contributions

B.Z.: Conception and study design, hESC manipulation, differentiation and characterization, in vitro and in vivo analyses, data interpretation, and writing of manuscript. J.P.: transplantation studies. K.R.: technical support with single-cell PCR. M.J.T.: technical advice. V.T.: study design and data interpretation. L.S.: conception and study design, data analysis and interpretation, and writing of manuscript.

## Supplemental Information

Document S1. Supplemental Experimental Procedures, Figures S1–S7, and Tables S1 and S2

Document S2. Article plus Supplemental Information

## References

[bib1] Bailey A.P., Bhattacharyya S., Bronner-Fraser M., Streit A. (2006). Lens specification is the ground state of all sensory placodes, from which FGF promotes olfactory identity. Dev. Cell.

[bib2] Bellin M., Marchetto M.C., Gage F.H., Mummery C.L. (2012). Induced pluripotent stem cells: the new patient?. Nat. Rev. Mol. Cell Biol..

[bib3] Bianchi A., Giampietro A., Pontecorvi A., De Marinis L. (2008). Isolated growth hormone deficiency: clinical entity?. J. Endocrinol. Invest..

[bib4] Chambers S.M., Qi Y., Mica Y., Lee G., Zhang X.J., Niu L., Bilsland J., Cao L., Stevens E., Whiting P. (2012). Combined small-molecule inhibition accelerates developmental timing and converts human pluripotent stem cells into nociceptors. Nat. Biotechnol..

[bib5] Chemaitilly W., Sklar C.A. (2010). Endocrine complications in long-term survivors of childhood cancers. Endocr. Relat. Cancer.

[bib6] Davis S.W., Mortensen A.H., Camper S.A. (2011). Birthdating studies reshape models for pituitary gland cell specification. Dev. Biol..

[bib7] Dincer Z., Piao J., Niu L., Ganat Y., Kriks S., Zimmer B., Shi S.H., Tabar V., Studer L. (2013). Specification of functional cranial placode derivatives from human pluripotent stem cells. Cell Rep..

[bib8] Dreser N., Zimmer B., Dietz C., Sugis E., Pallocca G., Nyffeler J., Meisig J., Bluthgen N., Berthold M.R., Waldmann T. (2015). Grouping of histone deacetylase inhibitors and other toxicants disturbing neural crest migration by transcriptional profiling. Neurotoxicology.

[bib9] Ericson J., Norlin S., Jessell T.M., Edlund T. (1998). Integrated FGF and BMP signaling controls the progression of progenitor cell differentiation and the emergence of pattern in the embryonic anterior pituitary. Development.

[bib10] Funato K., Major T., Lewis P.W., Allis C.D., Tabar V. (2014). Use of human embryonic stem cells to model pediatric gliomas with H3.3K27M histone mutation. Science.

[bib11] Lamolet B., Pulichino A.M., Lamonerie T., Gauthier Y., Brue T., Enjalbert A., Drouin J. (2001). A pituitary cell-restricted T box factor, Tpit, activates POMC transcription in cooperation with Pitx homeoproteins. Cell.

[bib12] Lee G., Ramirez C.N., Kim H., Zeltner N., Liu B., Radu C., Bhinder B., Kim Y.J., Choi I.Y., Mukherjee-Clavin B. (2012). Large-scale screening using familial dysautonomia induced pluripotent stem cells identifies compounds that rescue IKBKAP expression. Nat. Biotechnol..

[bib13] Leung A.W., Kent Morest D., Li J.Y. (2013). Differential BMP signaling controls formation and differentiation of multipotent preplacodal ectoderm progenitors from human embryonic stem cells. Dev. Biol..

[bib14] Maroof A.M., Keros S., Tyson J.A., Ying S.W., Ganat Y.M., Merkle F.T., Liu B., Goulburn A., Stanley E.G., Elefanty A.G. (2013). Directed differentiation and functional maturation of cortical interneurons from human embryonic stem cells. Cell Stem Cell.

[bib15] Maxwell M., Allegra C., MacGillivray J., Hsu D.W., Hedley-Whyte E.T., Riskind P., Madsen J.R., Black P.M. (1998). Functional transplantation of the rat pituitary gland. Neurosurgery.

[bib16] McDavid A., Finak G., Chattopadyay P.K., Dominguez M., Lamoreaux L., Ma S.S., Roederer M., Gottardo R. (2013). Data exploration, quality control and testing in single-cell qPCR-based gene expression experiments. Bioinformatics.

[bib17] Merkle F.T., Eggan K. (2013). Modeling human disease with pluripotent stem cells: from genome association to function. Cell Stem Cell.

[bib18] Merkle F.T., Maroof A., Wataya T., Sasai Y., Studer L., Eggan K., Schier A.F. (2015). Generation of neuropeptidergic hypothalamic neurons from human pluripotent stem cells. Development.

[bib19] Mica Y., Lee G., Chambers S.M., Tomishima M.J., Studer L. (2013). Modeling neural crest induction, melanocyte specification, and disease-related pigmentation defects in hESCs and patient-specific iPSCs. Cell Rep..

[bib20] Naik D.R., Das S., Patnaik L., Samantaray H. (1997). A novel and simple technique for ectopic transplantation of the pituitary gland. Gen. Comp. Endocrinol..

[bib21] Nunez L., Villalobos C., Senovilla L., Garcia-Sancho J. (2003). Multifunctional cells of mouse anterior pituitary reveal a striking sexual dimorphism. J. Physiol..

[bib22] Olson L.E., Tollkuhn J., Scafoglio C., Krones A., Zhang J., Ohgi K.A., Wu W., Taketo M.M., Kemler R., Grosschedl R. (2006). Homeodomain-mediated beta-catenin-dependent switching events dictate cell-lineage determination. Cell.

[bib23] Ozone C., Suga H., Eiraku M., Kadoshima T., Yonemura S., Takata N., Oiso Y., Tsuji T., Sasai Y. (2016). Functional anterior pituitary generated in self-organizing culture of human embryonic stem cells. Nat. Commun..

[bib24] Pagliuca F.W., Millman J.R., Gurtler M., Segel M., Van Dervort A., Ryu J.H., Peterson Q.P., Greiner D., Melton D.A. (2014). Generation of functional human pancreatic beta cells in vitro. Cell.

[bib25] Rao M. (2008). Scalable human ES culture for therapeutic use: propagation, differentiation, genetic modification and regulatory issues. Gene Ther..

[bib26] Regal M., Paramo C., Sierra S.M., Garcia-Mayor R.V. (2001). Prevalence and incidence of hypopituitarism in an adult Caucasian population in northwestern Spain. Clin. Endocrinol..

[bib27] Rezania A., Bruin J.E., Arora P., Rubin A., Batushansky I., Asadi A., O'Dwyer S., Quiskamp N., Mojibian M., Albrecht T. (2014). Reversal of diabetes with insulin-producing cells derived in vitro from human pluripotent stem cells. Nat. Biotechnol..

[bib28] Rivera J.A. (2006). Lymphocytic hypophysitis: disease spectrum and approach to diagnosis and therapy. Pituitary.

[bib29] Rosenfeld M.G., Briata P., Dasen J., Gleiberman A.S., Kioussi C., Lin C., O'Connell S.M., Ryan A., Szeto D.P., Treier M. (2000). Multistep signaling and transcriptional requirements for pituitary organogenesis in vivo. Recent Prog. Horm. Res..

[bib30] Scheithauer B.W., Horvath E., Kovacs K., Laws E.R., Randall R.V., Ryan N. (1986). Plurihormonal pituitary adenomas. Semin. Diagn. Pathol..

[bib31] Schwartz P.H., Brick D.J., Nethercott H.E., Stover A.E. (2011). Traditional human embryonic stem cell culture. Methods Mol. Biol..

[bib32] Schwartz S.D., Regillo C.D., Lam B.L., Eliott D., Rosenfeld P.J., Gregori N.Z., Hubschman J.P., Davis J.L., Heilwell G., Spirn M. (2015). Human embryonic stem cell-derived retinal pigment epithelium in patients with age-related macular degeneration and Stargardt's macular dystrophy: follow-up of two open-label phase 1/2 studies. Lancet.

[bib33] Sklar C.A., Constine L.S. (1995). Chronic neuroendocrinological sequelae of radiation therapy. Int. J. Radiat. Oncol. Biol. Phys..

[bib34] Smith J.C. (2004). Hormone replacement therapy in hypopituitarism. Expert Opin. Pharmacother..

[bib35] Smith S.M., Vale W.W. (2006). The role of the hypothalamic-pituitary-adrenal axis in neuroendocrine responses to stress. Dialogues Clin. Neurosci..

[bib36] Suga H., Kadoshima T., Minaguchi M., Ohgushi M., Soen M., Nakano T., Takata N., Wataya T., Muguruma K., Miyoshi H. (2011). Self-formation of functional adenohypophysis in three-dimensional culture. Nature.

[bib37] Szarek E., Farrand K., McMillen I.C., Young I.R., Houghton D., Schwartz J. (2008). Hypothalamic input is required for development of normal numbers of thyrotrophs and gonadotrophs, but not other anterior pituitary cells in late gestation sheep. J. Physiol..

[bib38] Tabar V. (2011). Making a pituitary gland in a dish. Cell Stem Cell.

[bib39] van Gelderen H.H., van der Hoog C.E. (1981). Familial isolated growth hormone deficiency. Clin. Genet..

[bib40] Villalobos C., Nunez L., Garcia-Sancho J. (2004). Phenotypic characterization of multi-functional somatotropes, mammotropes and gonadotropes of the mouse anterior pituitary. Pflugers Archiv..

[bib41] Wand G. (2008). The influence of stress on the transition from drug use to addiction. Alcohol Res. Health.

[bib42] Webster J.I., Sternberg E.M. (2004). Role of the hypothalamic-pituitary-adrenal axis, glucocorticoids and glucocorticoid receptors in toxic sequelae of exposure to bacterial and viral products. J. Endocrinol..

[bib43] Zhu Z., Huangfu D. (2013). Human pluripotent stem cells: an emerging model in developmental biology. Development.

[bib44] Zhu X., Gleiberman A.S., Rosenfeld M.G. (2007). Molecular physiology of pituitary development: signaling and transcriptional networks. Physiol. Rev..

[bib45] Zimmer B., Lee G., Balmer N.V., Meganathan K., Sachinidis A., Studer L., Leist M. (2012). Evaluation of developmental toxicants and signaling pathways in a functional test based on the migration of human neural crest cells. Environ. Health Perspect..

