# Supplementary material for: Derivation of Diverse Hormone-Releasing Pituitary Cells from Human Pluripotent Stem Cells
Source: Stem Cell Reports. 2016 Jun 14;6(6):858–72. doi: 10.1016/j.stemcr.2016.05.005 (PMC4912387; doi:10.1016/j.stemcr.2016.05.005)
Supplement: Document S1. Supplemental Experimental Procedures, Figures S1–S7, and Tables S1 and S2 [file mmc1.pdf]

Stem Cell Reports, Volume *6*

Supplemental Information

Derivation of Diverse Hormone-Releasing Pituitary Cells from Human Pluripotent Stem Cells

Bastian Zimmer, Jinghua Piao, Kiran Ramnarine, Mark J. Tomishima, Viviane Tabar, and Lorenz Studer

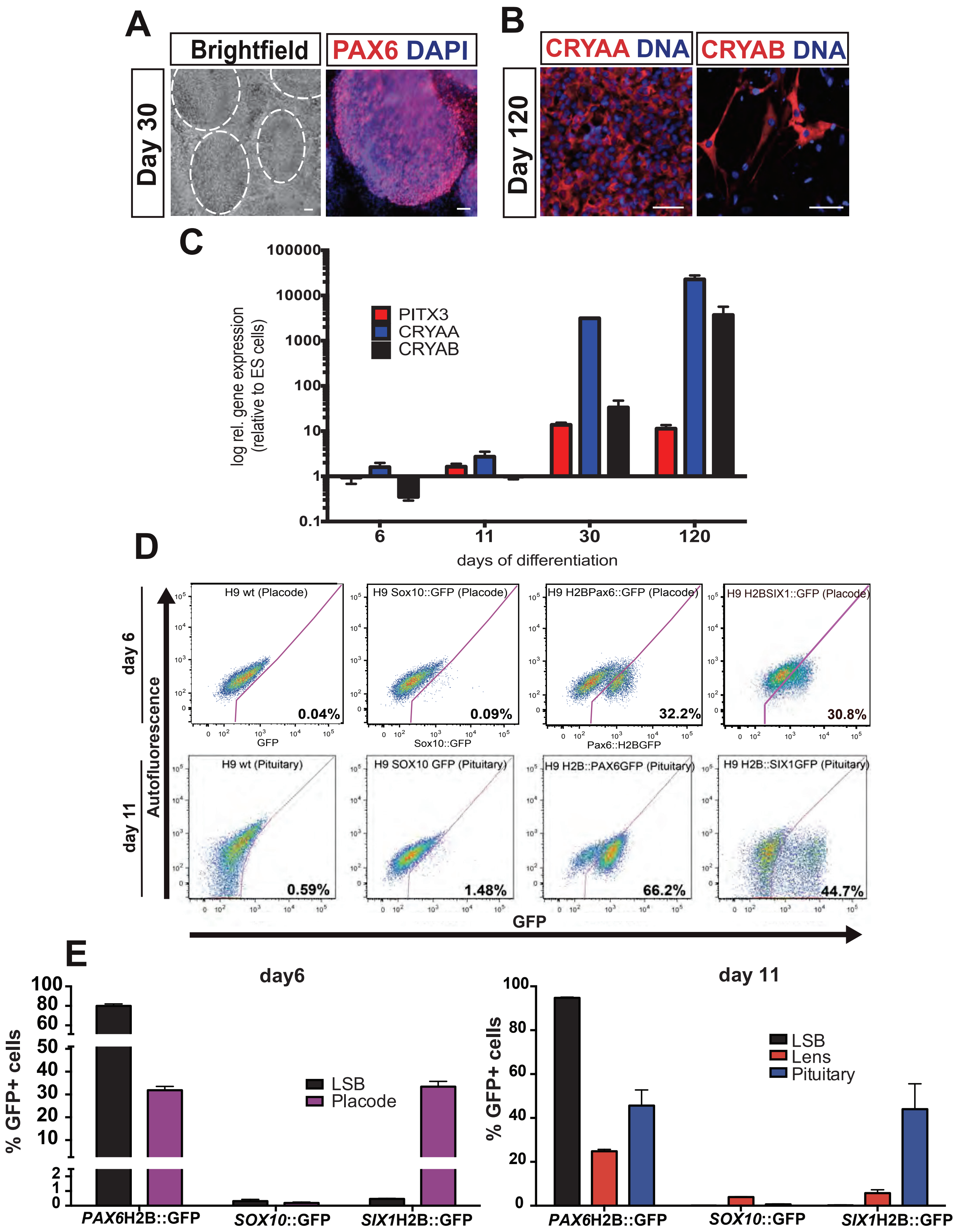

A
Brightfield
PAX6 DAPI
Day 30
B
CRYAA DNA
CRYAB DNA
Day 120
C
log rel. gene expression (relative to ES cells)
PITX3
CRYAA
CRYAB
days of differentiation
D
Autofluorescence
day 6
day 11
H9 wt (Placode)
H9 Sox10::GFP (Placode)
H9 H2BPax6::GFP (Placode)
H9 H2BSIX1::GFP (Placode)
0.04%
0.09%
32.2%
30.8%
H9 wt (Pituitary)
H9 SOX10 GFP (Pituitary)
H9 H2B::PAX6GFP (Pituitary)
H9 H2B::SIX1GFP (Pituitary)
0.59%
1.48%
66.2%
44.7%
GFP
E
day6
day 11
% GFP+ cells
LSB
Placode
LSB
Lens
Pituitary
PAX6H2B::GFP
SOX10::GFP
SIX1H2B::GFP

Supplemental Figure 1: "Default" conditions in chemically defined media result in lens placode specification and quantification of ectodermal subtypes within the pituitary differentiation using reporter cell lines, related to Figure 1.

(A) After 30 days of differentiation under "default" conditions (E6 only) lentoid bodies (circled structures in brightfield image) staining positive for the lens marker PAX6 are clearly identifiable. Scale bars: 50 µm. (B) After an additional 90 days of differentiation (day 120) the majority of the cells is expressing crystalline, the predominant structural proteins in the lens. Scale bars: 50 µm. (C) qRT-PCR gene expression time course during lens differentiation. Cells differentiated for 120 days express the lens characteristic transcripts PITX3, CRYAA and CRYAB. Values have been normalized to GAPDH and expression in undifferentiated ES cells and are plotted as means +/- SEM of 4 independent consecutive experiments. (D) Cells (different reporter cell lines) differentiated for 6 and 11 days under either default placode or pituitary conditions were analyzed using Flow Cytometry for SOX10, PAX6 or SIX1 expression. Representative Flow Cytometry plots with percentages are shown. (E) Quantification of data form A) reveals very few contaminating neural crest cells (SOX10+) while confirming the anterior cranial placode character of the cells (SIX1+, PAX6+). Data is plotted as mean ± SEM of 2-8 independent experiments.

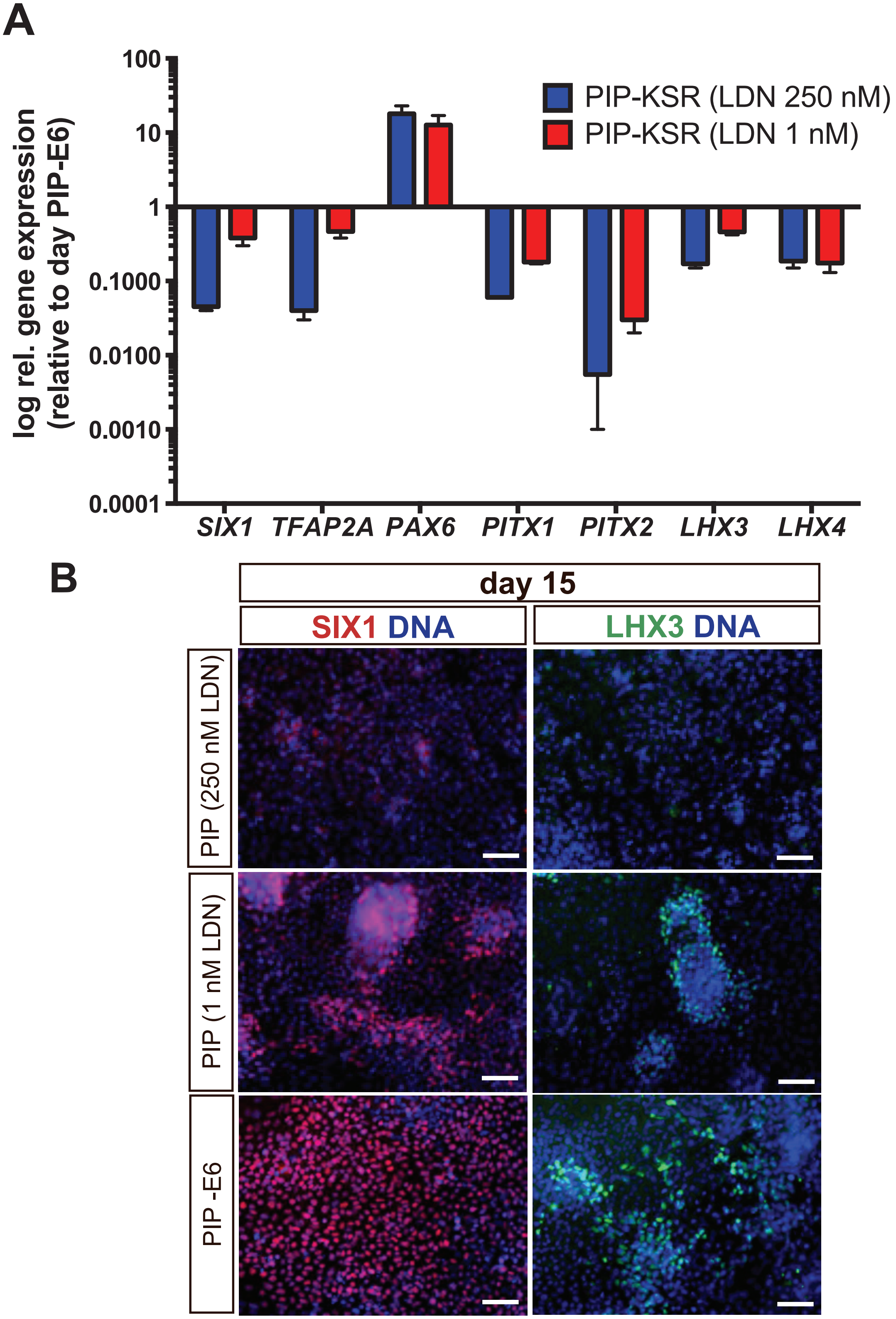

Supplemental Figure 2: Comparison of the traditional KSR-based pituitary induction with the new cGMP-ready induction, related to Figure 2.

Cells grown on feeders in KSR-based medium were differentiated using the old Dincer et. al. protocol (PIP-KSR). To compensate for KSR lot-to-lot variation 2 concentrations of LDN-193189 were used. Cells grown under feeder-free Essential8 conditions using the PIP-E6 protocol were differentiated in parallel. (A) qRT-PCR analysis of day 15 cells differentiated under PIP-KSR and PIP-E6 condition probing for SIX1, TFAP2A, PAX6, PITX1, PITX2, PITX3 and PITX4. Values have been normalized to GAPDH and expression in day 15 PIP-E6 cells and are plotted as means +/- SEM of 2 independent experiments. (B) Immunofluorescence comparison of cells differentiated for 15 days under either PIP-KSR or PIP-E6 condition. Cells were stained for either SIX1 (pan placode) or LHX3 (pan pituitary). Scale bars: 50 µm

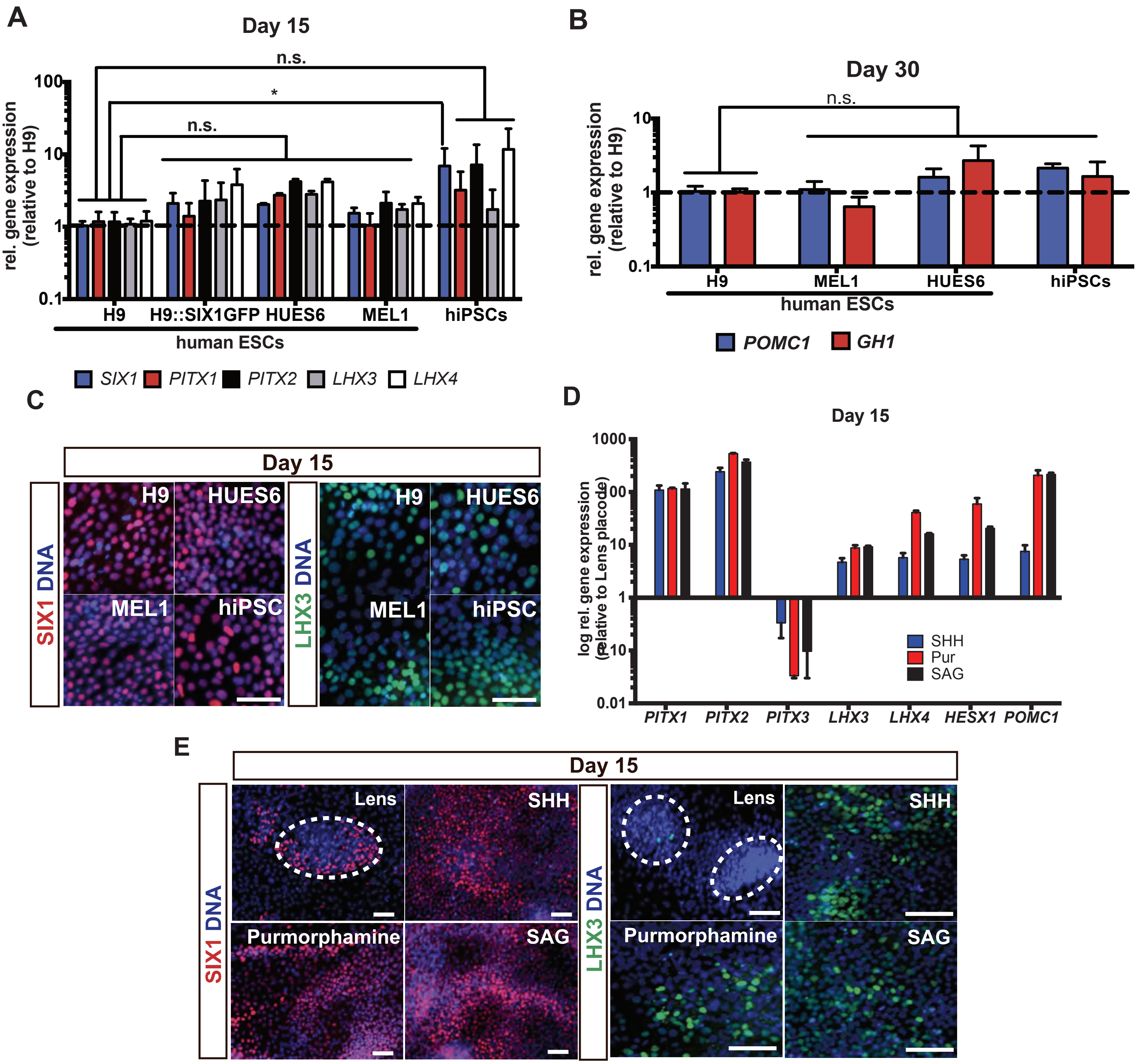

Supplemental Figure 3: Cell line comparison of pituitary induction protocol in E8/E6 and replacing recombinant SHH with small molecule smoothened agonists, related to Figure 2.

Four different hESC lines (including the H9 SIX1::H2B-GFP clone #6) and 1 hiPSC cell line were differentiated in parallel using the cGMP-ready pituitary induction protocol. (A) qRT-PCR analysis of day 15 cells differentiated under pituitary condition probing for the pan placodal marker SIX1 as well as the pan anterior pituitary genes PITX1, PITX2, LHX3 and LHX4. Values have been normalized to GAPDH and expression in day 30 wt H9 cells and are plotted as means +/- SEM of 2-4 independent experiments. (B) qRT-PCR analysis of day 30 cells differentiated under pituitary condition without sorting on day 15 probing for 2 anterior pituitary hormone transcripts POMC and GH1 Values have been normalized to GAPDH and expression in day 30 wt H9 cells and are plotted as means +/- SEM of 3 independent experiments. (C) Immunofluorescence analysis comparing protein expression on day 15 of pituitary placode induction across different hPSC lines. Scale bars: 50 µm. To investigate whether SHH can be replaced by small molecules, cells were differentiated using the cGMP-ready pituitary placode induction protocol using either recombinant SHH or one of the small molecule agonists purmorphamine or SAG in combination with FGF8 and FGF10. Lens placode differentiation was performed in parallel and served as a negative control. (D) qRT-PCR analysis of day 15 cells, differentiated under pituitary conditions using either SHH, purmorphamine or SAG from day 4 on, probing for the pan pituitary genes PITX1, PITX2, LHX3, LHX4, HESX1 as well as the hormone transcript POMC1 as well as the lens marker PITX3. Values have been normalized to GAPDH and expression in day 15 lens placode and are plotted as means +/- SEM of 3 independent experiments. (E) Immunofluorescence analysis comparing protein expression on day 15 of pituitary placode and lens induction using SHH and the small molecule alternatives purmorphamine and SAG. Early lentoid bodies (circled structures) start to downregulate expression of the pan placodal marker SIX1 while pituitary placode retains high SIX1 expression in combination with expression of LHX3. Scale bars: 50 µm

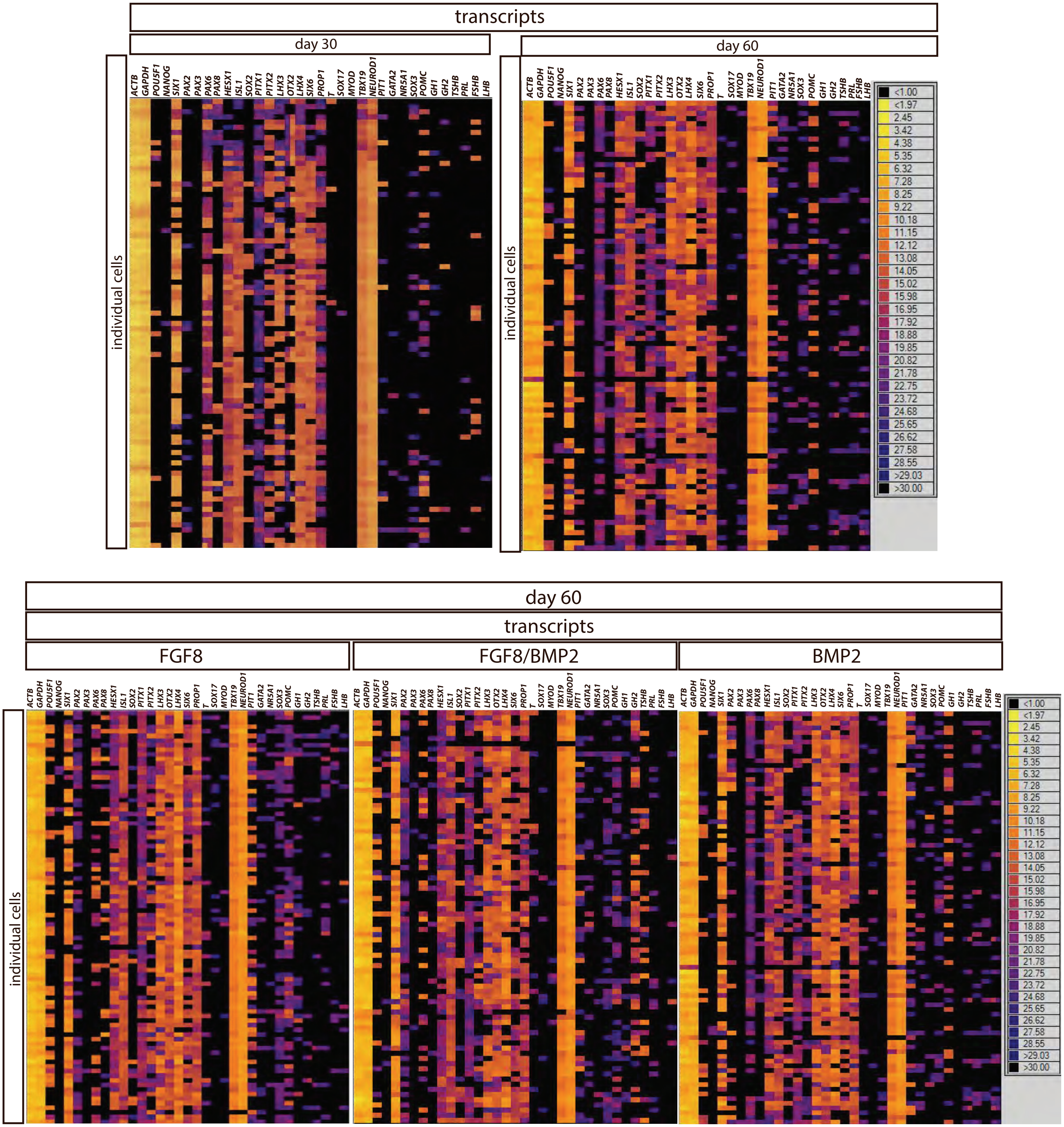

Supplemental Figure 4: Heatmaps of raw ct values for each cell and gene obtained by single cell q-RT PCR, related to Figure 5 and 6.

Raw ct values for every cell and gene obtained for every single cell PCR run are displayed as unprocessed heat maps.

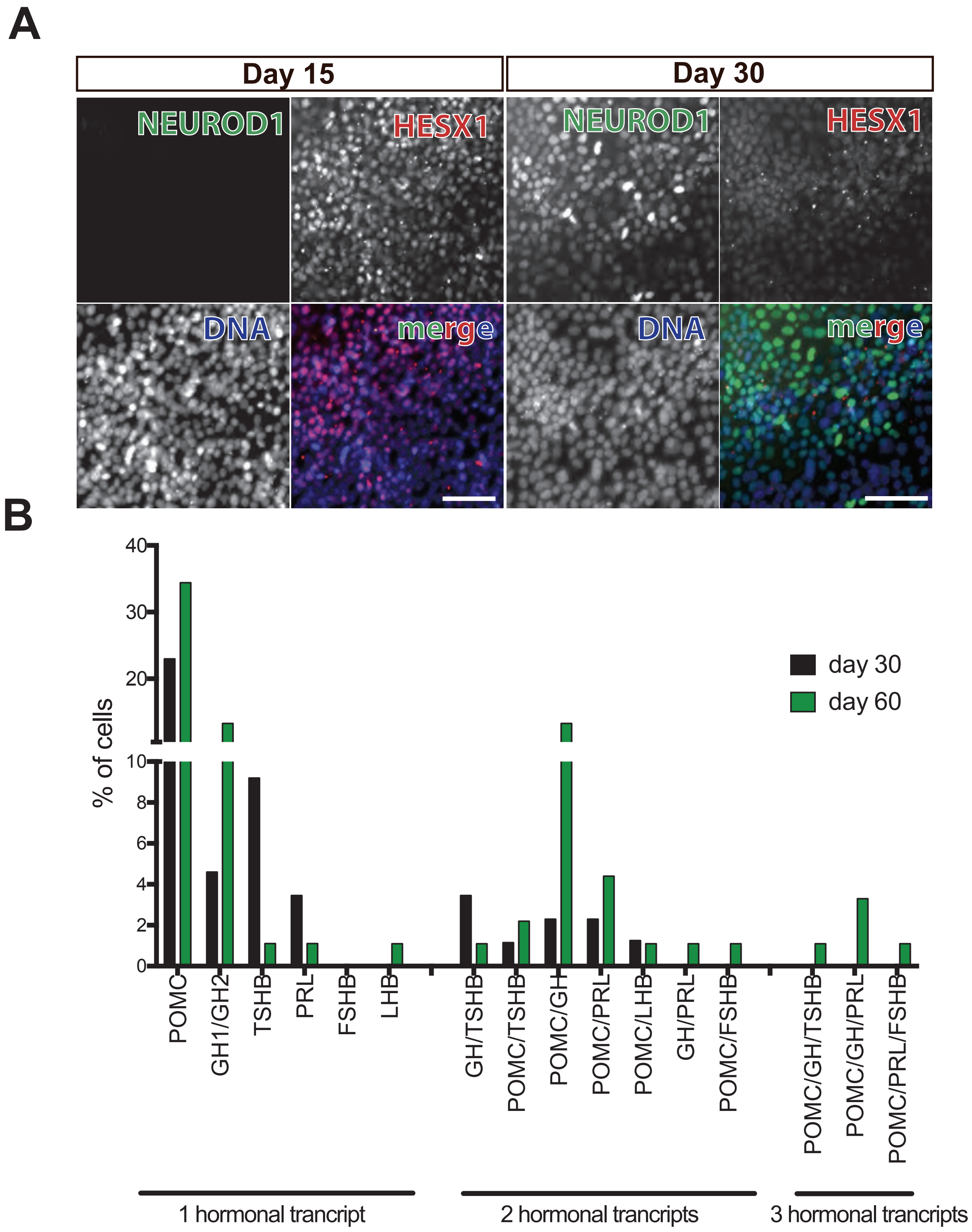

Supplemental Figure 5: Immunofluorescence validation of single cell q-RT PCR results and quantification of hormonal transcripts in single cells using single cell qRT-PCR, related to Figure 5.

(A) Immunofluorescence analysis of day 15 and day 30 cells differentiated under pituitary conditions. Cells were co-stained for the progenitor marker HESX1 and the transient cortiocotroph marker NEUROD1. Scale bars: 50 µm. (B) Single cell PCR data from day 30 and day 60 of the "default" pituitary differentiation protocol were mined for cells expressing at least one hormonal transcript. Data is plotted as percentage of cells expressing the respective transcript(s) (ct < 35 cycles in combination with a proper melting curve).

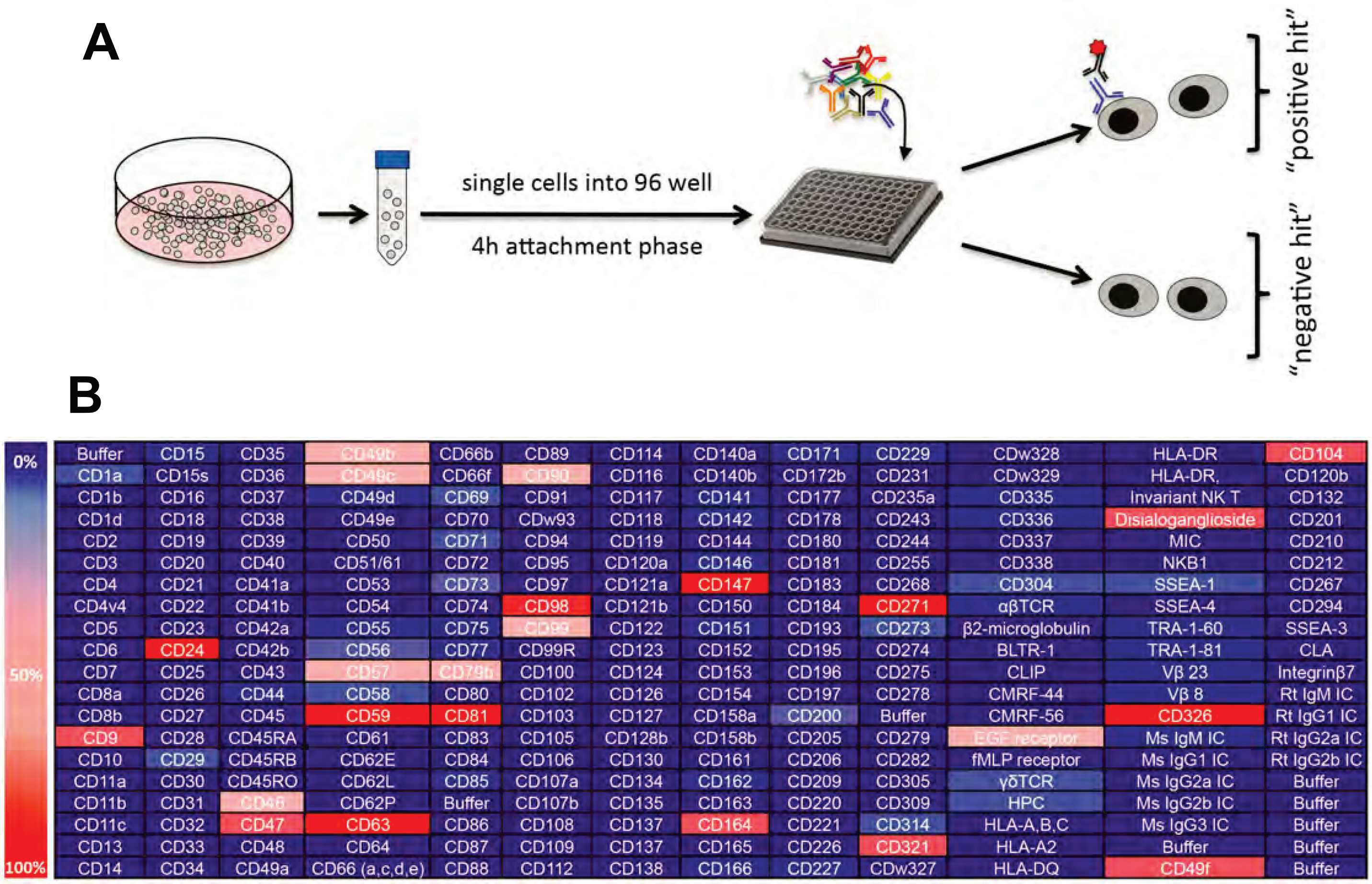

Supplemental Figure 6: Surface marker screen to identify hormonal subclass specific markers.

(A) Schematic representation of experimental procedure. (B) Heatmap of screen results. Percentage indicates cells staining positive for respective marker.

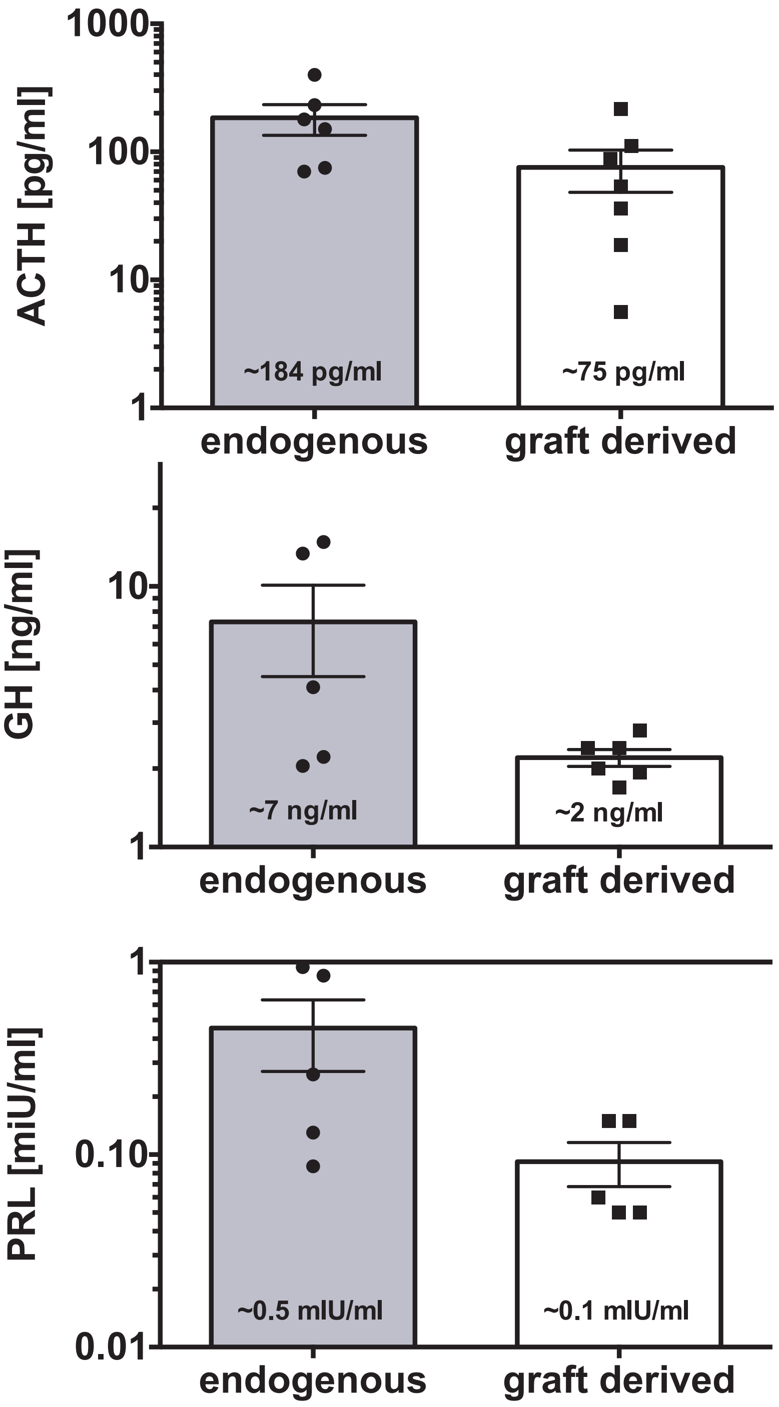

Supplemental Figure 7: Comparison of endogenous rat hormones in unlesioned animals and graft derived human hormones in lesioned animals, related to Figure 7.

Graft-derived hormone levels already presented in Main Figure 7 (week 5) are compared to endogenous hormone levels in unlesioned rats.

Supplementary Table S1: List of primers used in single cell qRT-PCR experiments

<table>
<tr><th>Target</th><th>FP</th><th>RP</th><th>Design RefSeq</th><th>Blast Hits</th><th>Gene Symbol</th><th>Gene Aliases</th></tr>
<tr><td>ACTB</td><td>CCAACCGCGAGAAGATGAC</td><td>TAGCACAGCCTGGATAGCAA</td><td>NM_001101.3</td><td>NM_001101</td><td>ACTB</td><td></td></tr>
<tr><td>FSHB</td><td>GTCGTTTCTGCATAAGCATCA</td><td>CTGGCTGGGTCCTTATACAC</td><td>NM_000510.2</td><td>NM_001018080.1|NM_000510.2</td><td>FSHB</td><td></td></tr>
<tr><td>GAPDH</td><td>GAACGGGAAGCTTGTCATCAA</td><td>ATCGCCCCACTTGATTTTGG</td><td>NM_002046.4</td><td>NM_002046|NM_001256799</td><td>GAPDH</td><td></td></tr>
<tr><td>GATA2</td><td>GCCTGTGGCCTCTACTACAA</td><td>GTCTGGATCCCTTCCTTCTTCA</td><td>NM_001145661.1</td><td>NM_032638|NM_001145661|NM_001145662</td><td>GATA2</td><td></td></tr>
<tr><td>GH1</td><td>ACTGGGCAGATCTTCAAGCA</td><td>GCAGCCCGTAGTTCTTGAGTA</td><td>NM_000515.3</td><td>NM_022560|NM_000515|NM_022559|NM_022562|NM_022561</td><td>GH1</td><td></td></tr>
<tr><td>GH2</td><td>CCCGTCGCCTGTACCA</td><td>TGTTGGAATAGACTCTGAGAAGCA</td><td>NM_022557.3</td><td>NM_022558|NM_002059|NM_022557</td><td>GH2</td><td></td></tr>
<tr><td>HESX1</td><td>GTTGGTATAGAGGCCGAAGACC</td><td>CCAGGATAGCAGTTTACTCTAAAGACA</td><td>NM_003865.2</td><td>NM_003865.2</td><td>HESX1</td><td></td></tr>
<tr><td>ISL1</td><td>TCGCCTTGCAGAGTGACATA</td><td>CCCGGTCCTCCTTCTGAAAA</td><td>NM_002202.2</td><td>NM_002202.2</td><td>ISL1</td><td></td></tr>
<tr><td>LHB</td><td>GCACCAAGGATGGAGATGC</td><td>GCTCCCTGGATGCCCAT</td><td>NM_000894.2</td><td>NM_000894</td><td>LHB</td><td>CGB4|hLHB|LSH-B</td></tr>
<tr><td>LHX3</td><td>TCGGACAAGGACAGCGTTCA</td><td>GCCCATTTCCGCCAAGGAA</td><td>NM_178138.3</td><td>NM_014564.2|NM_178138.3</td><td>LHX3</td><td>LIM3|CPHD3|M2-LHX3</td></tr>
<tr><td>LHX4</td><td>GGACTGTGGGGTTAGTGACA</td><td>CCCCACGTTGCCATAAATCC</td><td>NM_033343.2</td><td>NM_033343.2</td><td>LHX4</td><td>CPHD4</td></tr>
<tr><td>MYOD1</td><td>ACGAAGGCGCCTACTACA</td><td>GCAGTCTAGGCTCGACAC</td><td>NM_002478.4</td><td>NM_002478.4</td><td>MYOD1</td><td></td></tr>
<tr><td>NANOG</td><td>TGCCTTGCTTTGAAGCATCC</td><td>TTTCTTCAGGCCCACAAATCAC</td><td>NM_024865.2</td><td>NM_024865</td><td>NANOG</td><td></td></tr>
<tr><td>NEUROD1</td><td>GGCCCCAGGGTTATGAGACTA</td><td>ATCAGCCCACTCTCGCTGTA</td><td>NM_002500.4</td><td>NM_002500</td><td>NEUROD1</td><td></td></tr>
<tr><td>OTX2</td><td>AGGAGGTGGCACTGAAAATCA</td><td>CTGTTGTTGGCGGCACTTA</td><td>NM_021728.2</td><td>NM_172337.1|NM_021728.2</td><td>OTX2</td><td></td></tr>
<tr><td>PAX2</td><td>CGGCTGTGTCAGCAAAATCC</td><td>GCTTGGAGCCACCGATCA</td><td>NM_000278.3</td><td>NM_003989.3|NM_000278.3|NM_003987.3|NM_003988.3|NM_003990.3</td><td>PAX2</td><td></td></tr>
<tr><td>PAX3</td><td>GCGGTCTGTGATCGAAACA</td><td>TCCTCCTCTTCACCTTTCCC</td><td>NM_181461.3</td><td>NM_181459.3|NM_001127366.2|NM_000438.5|NM_181458.3|NM_181457.3|NM_013942.4|NM_181460.3|NM_181461.3</td><td>PAX3</td><td></td></tr>
<tr><td>PAX6</td><td>CCCCACATATGCAGACACACA</td><td>GAACTGACACACCAGGGGAAA</td><td>NM_001604.5</td><td>NM_001258465|NM_001258464|NM_001258463|NM_000280|NM_001604|NM_001127612|NM_001258462</td><td>PAX6</td><td></td></tr>
<tr><td>PAX8</td><td>GCCCAGTGTCAGCTCCATTA</td><td>GCTGTCCATAGGGAGGTTGAA</td><td>NM_003466.3</td><td>NM_013992.3|NM_013953.3|NM_013952.3|NM_003466.3</td><td>PAX8</td><td></td></tr>
<tr><td>PITX1</td><td>CCGTGTGGACCAACCTCA</td><td>GGTTACGCTCGCGCTTAC</td><td>NM_002653.4</td><td>NM_002653.4</td><td>PITX1</td><td>BFT|CCF|POTX|PTX1|LBNBG</td></tr>
<tr><td>PITX2</td><td>GCTGTGTGGACCAACCTTAC</td><td>CCCGAAGCCATTCTTGCATA</td><td>NM_000325.5</td><td>NM_000325.5|NM_153426.1|NM_153427.1</td><td>PITX2</td><td></td></tr>
<tr><td>POMC</td><td>CTCACCACGGAAAGCAACC</td><td>CTGCTCGTCGCCATTTCC</td><td>NM_000939.2</td><td>NM_000939.2|NM_001035256.1</td><td>POMC</td><td>LPH|MSH|NPP|POC|ACTH|CLIP</td></tr>
<tr><td>POU1F1</td><td>AGGAACTCAGGCGGAAAAGTA</td><td>CAGGGCCTCCCCAACA</td><td>NM_000306.2</td><td>NM_000306|NM_001122757</td><td>POU1F1</td><td>PIT1|CPHD1|GHF-1|Pit-1|POU1F1a</td></tr>
<tr><td>POU5F1</td><td>GGTATTCAGCCAAACGACCAT</td><td>CCGCAGCTTACACATGTTCT</td><td>NM_002701.4</td><td>NM_001173531|NM_203289|NM_002701</td><td>POU5F1</td><td></td></tr>
<tr><td>PRL</td><td>GGGCATGGAGCTGATAGTCA</td><td>TCCCGACCAGACAGGGTA</td><td>NM_000948.4</td><td>NM_000948.4|NM_001163558.1</td><td>PRL</td><td></td></tr>
<tr><td>PROP1</td><td>AGTCAGCCTTTGGGAGGAAC</td><td>GTGAGCGCTCTTGCTTCC</td><td>NM_006261.4</td><td>NM_006261</td><td>PROP1</td><td>CPHD2|PROP-1</td></tr>
<tr><td>SIX1</td><td>AGAACCGGAGGCAAAGAGAC</td><td>CTGCTTGTTGGAGGAGGAGTTA</td><td>NM_005982.3</td><td>NM_005982</td><td>SIX1</td><td></td></tr>
<tr><td>SIX6</td><td>AGGTGGGCAACTGGTTCA</td><td>CCCGGAACCCTGTGAC</td><td>NM_007374.2</td><td>NM_007374</td><td>SIX6</td><td>Six9|OPTX2|MCOPCT2</td></tr>
<tr><td>SOX17</td><td>CACAACGCCGAGTTGAGCAA</td><td>GCTCTGCCTCCTCCACGAA</td><td>NM_022454.3</td><td>NM_022454.3</td><td>SOX17</td><td></td></tr>
<tr><td>SOX2</td><td>CATGAAGGAGCACCCGGATTA</td><td>CGGGCAGCGTGTACTTATCC</td><td>NM_003106.2</td><td>NM_003106.2</td><td>SOX2</td><td></td></tr>
<tr><td>SOX3</td><td>GTGTGAAACGGCCCATGAAC</td><td>GTGCATCTTGGGGTTCTCCA</td><td>NM_005634.2</td><td>NM_005634</td><td>SOX3</td><td>PHP|GHDX|MRGH|PHPX|SOXB</td></tr>
<tr><td>T</td><td>CGCTTCAAGGAGCTCACCAA</td><td>GCCAGACACGTTCACCTTCA</td><td>NM_003181.2</td><td>NM_003181.2</td><td>T</td><td></td></tr>
<tr><td>TBX19</td><td>ACCCAGTTCATAGCCGTGAC</td><td>AGGCTTTGGCAAAAGGATTGTAC</td><td>NM_005149.2</td><td>NM_005149.2</td><td>TBX19</td><td>TPIT|TBS19|dJ747L4.1</td></tr>
<tr><td>TSHB</td><td>AATACCAGGATGCCCACTCC</td><td>GTATTGCACTTGCCACACTTACA</td><td>NM_000549.4</td><td>NM_001277991|NM_000549</td><td>TSHB</td><td>TSH-B|TSH-BETA</td></tr>
</table>

Supplementary Table S2. Antibodies used in this study, related to Figure 1, 2, 3, 4, 6, 7, S1, S2, S3 and S5

Antigen	Company	Catalogue #	dilution
ACTH	NIDDK	hACTH-IC	1:100
CRYAA	OriGene	CF505577	1:100
CRYAB	OriGene	CF500680	1:100
DLX3	Santa Cruz	Sc-98522	1:100
ECAD	BD Pharmingen	560062	1:200
EYA1	Avia Systems Biology	ARP32434_P050	1:200
FOXG1	StemCulture	NCFAB	1:1000
FSH	NIDDK	hBetaFSH-IC-3	1:100
GFP	Abcam	Ab13970	1:1000
GH	NIDDK	hGH-IC-2	1:100
HESX1	Sigma	HPA024187	1:500
hNA	Millipore	MAB1281	1:100
Ki67	DAKO	M7240	1:100
LH	NIDDK	hBetaLH-IC-3	1:100
LHX3 (rabbit)	Millipore	AB3202	1:300
LHX3 (mouse)	DSHB	67.4E12	1:100
NKX2.1 (TTF1)	Thermo Scientific	MS-699	1:200
NEUROD1	BD Pharmingen	563000	1:300
OCT4	Cell Signaling	2840	1:400
PAX3	DSHB	Pax3	1:100
PAX6	Covance	PRB-278P	1:800
PITX1	Sigma	HPA008743	1:500
PRL	NIDDK	hPRL-IC-5	1:100
SIX1	Sigma	HPA001893	1:500 -1:1000
SIX3/6	Santa Cruz	sc-9126 X	1:300
SOX10	Santa Cruz	sc-17342	1:100
SOX2	Cell Signaling	3579	1:400
TFAP2A	DSHB	3B5	1:100
TSH	NIDDK	hBetaTSH-IC-2	1:100

Supplemental experimental procedures

ESCs and culture conditions

The human pluripotent stem cells H9 (WA-09, XX, passage 35-50), MEL-1 (XY, passage 20-40), HUES-6 (XX, passage 24-40), hiPSCs (in-house generated hiPSCs derived from the fetal fibroblast cell line MRC5 (ATCC CCL-171) (Chambers et al., 2009), XY, passage 15-30) and modified reporter cell lines (all H9 background, passage 40-75) were maintained on VTN-N (Fisher Scientific) using Essential8 medium (E8) (Fisher Scientific) (Chen et al., 2011) and passaged twice a week using EDTA (Chen, 2008). Cells were tested for mycoplasma contamination once a month.

ESC differentiation

Differentiation into neural ectoderm was performed as previously described (Chambers et al., 2009) with slight modifications. Briefly, cells were plated at 250 000 cells/cm^2 on VTN-N coated dishes in E8 + Y-27632 (Tocris). After 24h (day0) medium was changed to Essential6 (E6) (Fisher Scientific) supplemented with 10 µM SB431542 (Tocris Biosciences), 500 nM LDN193189 (Stem Cell Technologies) and 1µM XAV939 (Tocris Biosciences) (until day 5). From day 5 on XAV939 was removed from the medium. Medium was changed every day until day 11.

Hypothalamic ectoderm differentiation was performed as described earlier (Maroof et al., 2013; Merkle et al., 2015) with slight modifications. Briefly, cells were plated at 250,000 cells/cm^2 on VTN-N coated dishes in E8 + Y-27632. After 24h (day0) medium was changed to E6 supplemented with 10 µM SB431542 for 2 days. From day 2 on E6 medium was supplemented with high concentrations of SHH (1 µg/ml) until day 11. For conditioned medium preparation cells were cultured for 24h in E6 only and washed twice afterwards to remove potential SHH from the induction medium. On day 13 E6 only was added to the cells and conditioned for 24h. Prior to using it, the conditioned medium was sterile filtered to eliminate debris and dead cells.

For lens differentiation cells were plated at 250 000 cells/cm^2 on VTN-N coated dishes in E8 + 10 µM Y-27632. After 24h (day0) medium was changed to E6 supplemented with 10 µM SB431542 and 5 ng/ml BMP4 (R&D Systems). Medium was changed every day. On day 3 BMP4 was removed from the medium and cells were cultured in E6 + 10 µM SB431542 until day 15. From day 15 on cells were maintained in E6 only for up to 120 days. From day 30 on, medium was supplemented with VTN-N (1:100) once a week during feeding to prevent cells from peeling of the plate.

For pituitary differentiation cells were plated at 250 000 cells/cm^2 on VTN-N coated dishes (differentiation works best in 24 well plates) in E8 + 10 µM Y-27632. After 24h (day0) medium was changed to E6 supplemented with 10 µM SB431542 and 5 ng/ml BMP4. Medium was changed every day. On day 3 BMP4 was removed from the medium and cells were cultured for 1 day in E6 + 10 µM SB431542. For the standard differentiation conditions, E6 was supplemented with 10 µM SB431542, 200 ng/ml SHH (R&D Systems, C25II), 100 ng/ml FGF8b (R&D Systems) and 50 ng/ml FGF10 (Peprotech Inc.) on day 4. For some experiments SHH was replaced by 1 µM purmorphamine (Stemgent) or 1 µM SAG (Stemcell Technologies) From now on medium volume was doubled and cells were feed every other day until day 15. On day 15 of differentiation *SIX1::H2B-GFP*$^+$ cells were sorted using a BDFACS Aria III cell sorter. Purified cells were then plated as droplets (50,000 cells/10 µl drop) in E6 supplemented with 10 µM Y-27632, 200 ng/ml SHH, 100 ng/ml FGF8b and 50 ng/ml FGF10 on poly-ornithine/laminin/fibronectin-coated plates. After 24h medium was changed to E6 containing SHH, FGF8 and FGF10 until day 30. Medium was changed every other day. For some experiments pituitary induction was started slightly later (day 6) or cells were differentiated in medium conditioned by hypothalamic neuroectoderm from either day 4 or day 6 on. For the co-culture experiment *SIX1::H2B-GFP* positive cells were sorted on day 6 and 50,000 cells/cm^2 were plated directly on hypothalamic neuroectodermal cells in E6 only supplemented with 10 µM SB431542.

Pituitary cell maturation and subtype specification

Unless otherwise stated in the text, standard pituitary maturation medium on day 30 was changed to "E6 only" for additional 30 days. For patterning experiments (indicated in the text) E6 medium was supplemented with high concentrations of either FGF8 (100 ng/ml, dorsalize) or BMP2 (20 ng/ml, ventralize) or intermediate concentrations of both (FGF8 50 ng/ml, BMP2 10 ng/ml).

RNA extraction and traditional quantitative Real-time PCR
Total RNA was extracted from at least 3 independent experiments using the TRIzol (Fisher Scientific) reagent in combination with Phase-lock tubes (5Prime) according to the manufactures protocol. 1 μg of total RNA was reverse transcribed into cDNA using iScript (BioRad). For quantitative RT-PCR we used the SSoFast EvaGreen Mix (BioRad) in combination with QuantiTect primer assays (Qiagen) on a BioRad CFX96 Thermal Cycler. All reactions were run according to the manufacturer's protocol. Gene expression was normalized to glyceraldehyde 3-phosphate dehydrogenase (*GAPDH*) and a control cell type (indicated in the Figures). Results are calculated using the ΔΔCt method (Livak and Schmittgen, 2001).

Single Cell quantitative RT-PCR
For single cell PCR analysis cells were detached using Accutase. After filtering through a 40 μm cell strainer DAPI negative cells were sorted on a BDFACS Aria III machine to select for live cells. Sorted cell suspension was adjusted to a concentration of 400,000 cells/ml. Single cells were captured using the Fluidigm C1 system according to manufacturer's protocol. Capture rate of for each C1 chip (C1 Single-cell Auto Prep Array IFC (10 -17 μm)) was confirmed microscopically using a standard tissue culture brightfield microscope. Capture rates were as follows: day 30: 91% (87/96) day 60: 94% (90/96) day 60 FGF8: 93% (89/96) day 60 FGF8/BMP2: 89% (85/96) day 60 BMP2: 93% (89/96). Cells were lysed, RNA was extracted and transcribed into cDNA and pre-amplified using the C1 in combination with wet-lab tested Fluidigm DELTAgene assays following the manufacturer's protocol. The resulting cDNA was diluted 1:5 and subjected to single-cell PCR amplification on a Fluidigm 96.96 Dynamic Array using the Fluidigm BioMark system in combination with EvaGreen chemistry according to the manufacturer's manual ("Fast Gene Expression Analysis Using EvaGreen on the BioMark or BioMark HD System"). Each primer pair was run in technical duplicates on the chip. Only single cells with consistent amplification results between the technical primer replicates were considered positive to minimize false positive calls. Overall discrepancy rate was low (< 3% per primer pair). Expression data was analyzed using the Fluidigm Real-Time PCR analysis software in combination with the Fluidigm SINGuLAR Analysis Toolset for R (Version 3.0.2 (2013-09-25) "Frisbee Sailing").

Microscopy, Antibodies and Flow Cytometry
After washing the cells once with PBS, cells were fixed with 4% (v/v) paraformaldehyde for 20 min, washed twice with PBS, permeabilized using 0.1% (v/v) Triton X-100 in PBS, Cells were blocked with 10% (v/v) FCS in PBS for 1-5h at room temperature. Cells were incubated with primary antibodies diluted in 2% FCS (v/v) in PBS at 4°C overnight. A list of the primary antibodies used in this study is provided as **Table S2**. After primary antibody incubation cells were washed twice with PBS followed by incubation with appropriate AlexaFluor-conjugated secondary antibodies diluted in PBS at room temperature for 1h (1:1000; Molecular Probes, Thermo Fisher). After washing twice with PBS, nuclei were stained using DAPI. After an additional 2 washing steps, fluorescence images of the cells were acquired using an Olympus IX71 inverted microscope equipped with a Hamamatsu ORCA CCD camera. For immunohistochemical analysis, the animals were perfused with PBS and then 4% paraformaldehyde. Matrigel plugs were post-fixed in 4% paraformaldehyde and subsequently immersed in 30% sucrose. Matrigel plugs were cryosectioned at 30 μm for immunohistochemical analysis. The sections were pretreated with Antigen Retrieval Reagent-Universal solution (R&D systems). The sections were washed with PBS and then blocked with blocking solution (1%BSA-0.3%Triton-PBS) for 1 hour at room temperature. The sections were stained with hNA, Ki67, ACTH, GH, TSH, PRL, FSH and LH and subsequently with an Alexa-568 conjugated secondary antibody. The images were acquired using an Olympus BX51 Microscope equipped with a Hamamatsu camera. Stereological quantification of the number of ACTH cells in the whole matrigel plug was conducted using the optical fractionator probe, and the graft volume was analyzed using the Cavalieri estimator method. (Stereo Investigator Software, Microbrightfield Bioscience). For flow analysis cells (different reporter cell lines) were detached from cell culture plastic using TrypLE (Fisher Scientific). After washing once with PBS cells were resuspended in 2% FCS, 1 mM EDTA in PBS and DAPI. Cells were filtered using a 40 μm cell strainer and analyzed on BD LSRFortessa Flow Cytometer. Only single (doublet exclusion) live (DAPI-) cells were analyzed. Data was further processed using FlowJo Version 9.7.6 (FLOWJO LLC).

Cell Surface Marker Screen
For the BD Lyoplate™ cell surface marker screen day 30 cells were replated at a density of 100 000 cells/cm^2 into 96 well imaging plates using Accutase. After a 4 hour attachment phase cells were stained according to the user's manual for bioimaging. Cells were analyzed on an Operetta High Content Imaging System (Perkin Elmer). Images were processed and analyzed using the Harmony Software package (Perkin Elmer).

Stimulation of hormone release
To stimulate hormone release *in vitro*, cells were differentiated in 24 well plates as described above. On day 30 of differentiation cells were washed once with PBS and 250 µl of fresh medium containing either the solvent or the stimulant were added to each well. After 12 h the supernatant was removed and centrifuged for 5 min at 2000g to pellet debris. Supernatant was transferred into fresh reaction tubes, flash frozen and stored at -80ºC until ELISA measurements. Stimulants used were: CRF (Tocris, 1 µM), Stressin I (Tocris, 2 µM), Ghrelin (Tocris, 1 µM), Somatocrinin (Accurate Chemical, 1 µg/ml), Nafarelin (Tocris, 1 µM) and Urocortin (Tocris, 500 nM).

ELISA measurements
Hormone concentration in the supernatant of cells or in animal plasma was analyzed using ELISA measurements. Hormone concentration in the cell culture supernatant was assessed using traditional single hormone ELISA Kits according to the manufacturer's manual (ACTH (Calbiotech, detects rat and human ACTH)), hGH (R&D Systems, human specific), FSH (Calbiotech, FSH (lumELISA, human specific) and corticosterone (Abcam)). Plates were read using an EnSpire Multimode plate reader (PerkinElmer). Hormone concentration for *in vivo* samples was analyzed using either traditional ELISA (for ACTH only, serum diluted 1:2) or species specific (human or rat) Milliplex multiplex ELISA using Luminex technology (Millipore). Magnetic bead-based sandwich immunoassay was performed according to the manufacturer's manual. 25 µl of undiluted serum samples in duplicate wells were analyzed by Luminex FlexMap 3D (Luminex Corp, Austin, TX). Cytokine concentrations were determined by Luminex Xponent 4.1 and EMD-Millipore Milliplex Analyst v5.1 using 5-p log analysis.

Animal maintenance
Male Athymic nude rats (RNU rat Crl:NIH-Foxn1rnu, Charles River Laboratories) were hypophysectomized using the parapharyngeal approach at the age of 8 weeks by Charles Rivers Laboratories. Plasma ACTH was measured 1 week after hypophysectomy to confirm hypopituitarism. The hypophysectomized rats were randomized into two groups: Sham control (n=4), Human ES derived pituitary cells subcutaneously grafted group (n=7). Two million human ES-derived pituitary cells were suspended in matrigel (BD Biosciences) and injected subcutaneously. Blood was taken by retro orbital bleeding before graft, 1 week, 3 weeks, 5 weeks and 7 weeks after the transplantation under isoflurane anesthesia at 8 a.m. Blood was collected with K2 EDTA-treated BD Microtainer MAP (BD Biosciences) and plasma was isolated and stored at -80ºC. All animal procedures were done in accordance with protocols approved by Animal Care and Use Committee at MSKCC and following NIH guidelines.

Statistical analysis
Data are presented as sample means ± SEM, as indicated in each figure legend. Means represent the data of independent experiments (number indicated in the corresponding figure legend). Differences between groups were analyzed by unpaired t tests or one-way ANOVA with Bonferroni multiple-comparison post hoc test. $p < 0.05$ = *, $p < 0.01$ = **, $p < 0.001$ = ***, $p < 0.0001$ = ****

Supplemental References

Chambers, S.M., Fasano, C.A., Papapetrou, E.P., Tomishima, M., Sadelain, M., and Studer, L. (2009). Highly efficient neural conversion of human ES and iPS cells by dual inhibition of SMAD signaling. Nature biotechnology *27*, 275-280.
Chen, G. (2008). Splitting hESC/hiPSC lines with EDTA in feeder free conditions. In StemBook (Cambridge (MA)).

Chen, G., Gulbranson, D.R., Hou, Z., Bolin, J.M., Ruotti, V., Probasco, M.D., Smuga-Otto, K., Howden, S.E., Diol, N.R., Propson, N.E., *et al.* (2011). Chemically defined conditions for human iPSC derivation and culture. Nat Methods *8*, 424-429.

Livak, K.J., and Schmittgen, T.D. (2001). Analysis of relative gene expression data using real-time quantitative PCR and the 2(-Delta Delta C(T)) Method. Methods *25*, 402-408.

Maroof, A.M., Keros, S., Tyson, J.A., Ying, S.W., Ganat, Y.M., Merkle, F.T., Liu, B., Goulburn, A., Stanley, E.G., Elefanty, A.G., *et al.* (2013). Directed differentiation and functional maturation of cortical interneurons from human embryonic stem cells. Cell stem cell *12*, 559-572.

Merkle, F.T., Maroof, A., Wataya, T., Sasai, Y., Studer, L., Eggan, K., and Schier, A.F. (2015). Generation of neuropeptidergic hypothalamic neurons from human pluripotent stem cells. Development *142*, 633-643.
