# Supplementary material for: Derivation of Diverse Hormone-Releasing Pituitary Cells from Human Pluripotent Stem Cells
Source: Stem Cell Reports. 2016 Jun 14;6(6):858–72. doi: 10.1016/j.stemcr.2016.05.005 (PMC4912387; doi:10.1016/j.stemcr.2016.05.005)
Supplement: Document S2. Article plus Supplemental Information [file mmc2.pdf]

Stem Cell Reports
Article
OPEN ACCESS

Derivation of Diverse Hormone-Releasing Pituitary Cells from Human Pluripotent Stem Cells

Bastian Zimmer,[1,2] Jinghua Piao,[1,3] Kiran Ramnarine,[1,4] Mark J. Tomishima,[1,4] Viviane Tabar,[1,3] and Lorenz Studer[1,2,3,*]
[1]The Center for Stem Cell Biology
[2]Developmental Biology Program
Sloan Kettering Institute for Cancer Research, 1275 York Avenue, New York, NY 10065, USA
[3]Department of Neurosurgery, Memorial Sloan Kettering Cancer Center, 1275 York Avenue, New York, NY 10065, USA
[4]SKI Stem Cell Research Facility, 1275 York Avenue, New York, NY 10065, USA
*Correspondence: studerl@mskcc.org
http://dx.doi.org/10.1016/j.stemcr.2016.05.005

SUMMARY

Human pluripotent stem cells (hPSCs) provide an unlimited cell source for regenerative medicine. Hormone-producing cells are particularly suitable for cell therapy, and hypopituitarism, a defect in pituitary gland function, represents a promising therapeutic target. Previous studies have derived pituitary lineages from mouse and human ESCs using 3D organoid cultures that mimic the complex events underlying pituitary gland development in vivo. Instead of relying on unknown cellular signals, we present a simple and efficient strategy to derive human pituitary lineages from hPSCs using monolayer culture conditions suitable for cell manufacturing. We demonstrate that purified placode cells can be directed into pituitary fates using defined signals. hPSC-derived pituitary cells show basal and stimulus-induced hormone release in vitro and engraftment and hormone release in vivo after transplantation into a murine model of hypopituitarism. This work lays the foundation for future cell therapy applications in patients with hypopituitarism.

INTRODUCTION

Human pluripotent stem cells (hPSCs) provide a unique resource for basic as well as translational research. Both human embryonic stem cells (hESCs) and human induced pluripotent stem cells (hiPSCs) are widely used to study early human development (Zhu and Huangfu, 2013), assess the toxic effects of chemicals (Dreser et al., 2015; Zimmer et al., 2012), model human diseases or cancer (Bellin et al., 2012; Funato et al., 2014; Merkle and Eggan, 2013), and discover novel potential drugs (Lee et al., 2012). Furthermore, access to greatly improved protocols for lineage-specific differentiation has led to the first experimental applications of hPSC-derived lineages in regenerative medicine such as in patients with macular degeneration (Schwartz et al., 2015). Other hPSC-based applications that are being pursued intensely include the replacement of hormone-producing cells such as in type 1 diabetes (Pagliuca et al., 2014; Rezania et al., 2014). Replacing hormone-producing cells is a particularly attractive approach for cell therapy, especially if restoration of feedback mechanisms with subsequent dynamic release of hormones can be achieved by the grafted cells.

The pituitary gland is considered the master gland of hormone function. Hypopituitarism is a disease condition with insufficient or absent function of the pituitary gland. Pituitary tumors are the most common cause but many other triggers can induce pituitary dysfunction including inborn genetic defects, brain trauma, immune and infectious diseases, or radiation therapy. The prevalence of hypopituitarism has been estimated at 46 per 100,000 (Regal et al., 2001), but this is likely an underestimation. The consequences of pituitary dysfunction are particularly serious in children where they can lead to severe learning disabilities, growth and skeletal problems, as well as effects on puberty and sexual function (Chemaitilly and Sklar, 2010). Chronic hypopituitarism requires lifelong complex hormone replacement therapies that are very costly and compromise quality of life. Furthermore, static delivery of hormones can only poorly mimic the dynamic secretion of the intact pituitary gland, which reacts to feedback mechanisms such as the hypothalamic-pituitary-adrenal (HPA) axis or the circadian clock. Therefore, there is a considerable clinical need to direct current treatment paradigms toward a more physiological and complete hormone replacement therapy (Smith, 2004).

It is conceivable that replacing the damaged cells via cell transplantation can restore pituitary function and permanently cure chronic hypopituitarism. Previous work in mouse ESCs has shown that anterior pituitary cells, capable of hormone secretion, can be generated in 3D cultures by recapitulating some of the complex morphogenetic interaction between the developing hypothalamic and oral ectoderm tissues in vitro (Suga et al., 2011). Our laboratory has recently reported a first attempt at generating functional adenohypophyseal cells from human PSCs (Dincer et al., 2013), and very recently pituitary cells have been generated from hPSCs using a 3D organoid approach (Ozone et al., 2016). While these studies represent a promising proof of concept, current protocols remain

This is an open access article under the CC BY license (http://creativecommons.org/licenses/by/4.0/).

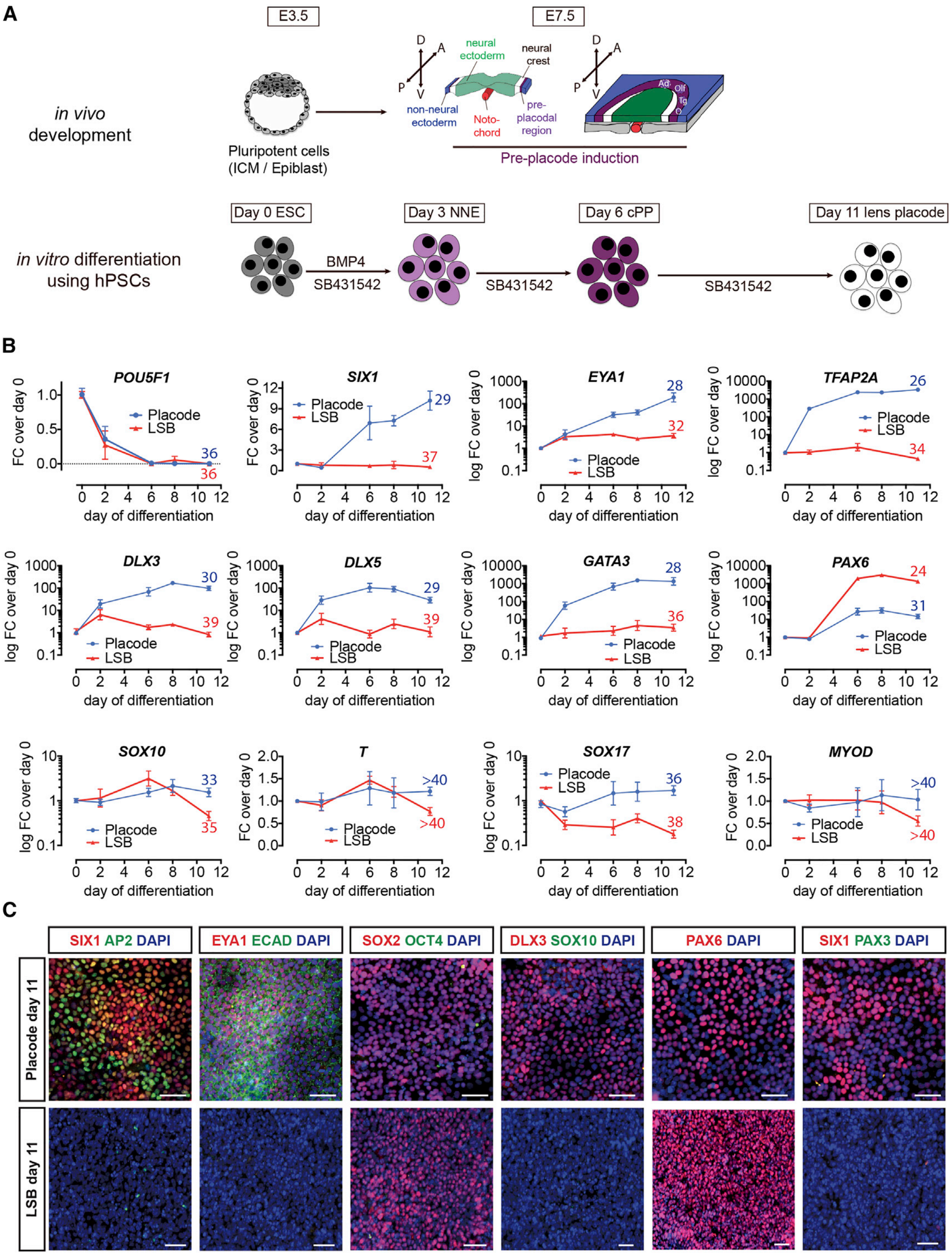

Figure 1. Differentiation of hPSCs into Cranial Placode Using Chemically Defined Conditions

(A) Schematic representation of cranial placode in vivo development and protocol for directed differentiation of hPSCs. ICM, inner cell mass.

(B) Real-time PCR gene expression time course of key cranial placode (*SIX1*, *EYA1*) and non-neural ectoderm (*TFAP2A*, *DLX3/5*, *GATA3*) genes as well as genes probing for potential contaminates (*SOX10*, *T*, *SOX17*, *MYOD*). Values are normalized to *GAPDH* and expression on

(legend continued on next page)

inefficient, poorly defined, and unsuitable for developing current good manufacturing practice (cGMP)-compatible culture conditions that will be eventually required for human therapeutic use.

Here, we report the efficient derivation of anterior pituitary cells from hPSCs in clinically compatible and scalable culture conditions. We further characterize the diversity of anterior pituitary subtypes achieved in vitro using single-cell mRNA expression analysis. The resulting hPSC-derived pituitary cells are functional in vitro, respond to appropriate stimuli, and are capable of secreting hormones in an animal model of hypopituitarism in vivo. Importantly, our data indicate that pituitary cell fate can be induced independent of mimicking the complex 3D organization of the developing gland. We demonstrate that by providing appropriate signals to purified placode precursor cells, pituitary identity can be specified at high efficiency, and that further manipulations of morphogen gradients enable controlled changes in the relative composition of hormonal cell types. In conclusion, we provide a robust differentiation platform to access diverse hormone-producing cell types suitable for further development toward a cell-based treatment of hypopituitarism.

RESULTS

Derivation of Cranial Placode from hPSCs under Fully Defined Conditions

The anterior pituitary gland is derived from cranial placode cells that form from the oral ectoderm. Therefore, the first step in establishing a defined protocol is the efficient induction of cranial placode cells competent in generating anterior pituitary lineages. The cranial placode induction protocol (PIP) presented here relies on serum-free monolayer-based induction conditions, uses fully defined cGMP-ready components, and eliminates ill-defined factors such as knockout serum replacement (KSR), Matrigel, or mouse embryonic fibroblast feeders that were part of previous protocols (Dincer et al., 2013). The specific factors used to trigger placode induction are based on signals shown to specify placode development in vivo (Figure 1A). We observed that exposure to moderate concentrations of bone morphogenetic protein 4 (BMP4) efficiently induced cranial placode cells that adopted by "default" a lens fate (Figures S1A–S1C). These results are in agreement with previous efforts of inducing placode fate from hPSCs and with data in the developing chick embryo reporting a lens default in the absence of fibroblast growth factor (FGF) signals (Bailey et al., 2006; Leung et al., 2013). Temporal expression analysis revealed the rapid loss of pluripotency markers and induction of key placode genes such as *SIX1*, *EYA1*, and *DLX3/5* after 6 days of differentiation at both mRNA (Figure 1B) and protein (Figure 1C) levels. Transcripts of contaminating cell types such as *SOX10* (neural crest), *SOX17* (endoderm), *T/Brachyury* (mesoderm), or *MYOD* (myogenic lineages) were not induced under these conditions (Figure 1B). Our previous study reported PAX3$^+$ trigeminal placode as the "default" identity of hPSC-derived placode cells (Dincer et al., 2013). Our current method, using the defined placode induction conditions, shows PAX6 rather than PAX3 expression (Figure 1C). To further quantify the yield and selectivity of placode induction and PAX6 expression, we used hESC genetic reporter lines for *SIX1*::*H2B-GFP*, *PAX6*::*H2B-GFP*, *SOX10*::*GFP* (Chambers et al., 2012; Mica et al., 2013). Flow analysis confirmed robust induction of PAX6 and SIX1 without SOX10, consistent with anterior cranial placode in the absence of contaminating neural crest cells (Figures S1D and S1E).

Patterning of Anterior Pituitary from hPSC-Derived Cranial Placode

The complex morphogenetic development of the pituitary gland occurs during early embryonic stages (~embryonic day 10 [E10] in mouse and between embryonic weeks 4 and 8 in human). Both the anterior and intermediate lobes of the pituitary gland are derived from oral ectoderm, which corresponds to the pituitary placode while the posterior pituitary gland develops from the neural ectoderm (Figure 2A). Inductive tissue interactions as well as various defined signaling pathways including FGFs, BMPs, and Sonic hedgehog (SHH) are thought to be important for proper gland development and hormonal subtype specifications in vivo (Zhu et al., 2007) (Figure 2A, upper panel). Here we assessed the ability of these developmental pathways to induce pituitary identity (Figure 2A, lower panel). Our data show that timed exposure to SHH, FGF8, and FGF10 robustly induces gene expression associated with anterior pituitary development including *PITX1/2*, *LHX3/4*, *HESX1*, and *SIX6* (Figure 2B). Where possible, we confirmed expression at the protein level using antibodies against PITX1, LHX3, HESX1, and SIX6 (Figure 2C). We compared our cGMP-ready E8/E6-based induction protocol with our published KSR-based PIP (Dincer et al., 2013). To

day 0 of differentiation (directly before switch to differentiation medium) and plotted as mean ± SEM from four independent differentiations. Numbers above day-11 data point indicate average raw ct values for better comparison.
(C) Immunofluorescence analysis comparing protein expression on day 11 of cranial placode induction protocol and LSB (LDN-193189/SB-431542; neuroectoderm). Scale bars, 50 µm.
See also Figure S1.

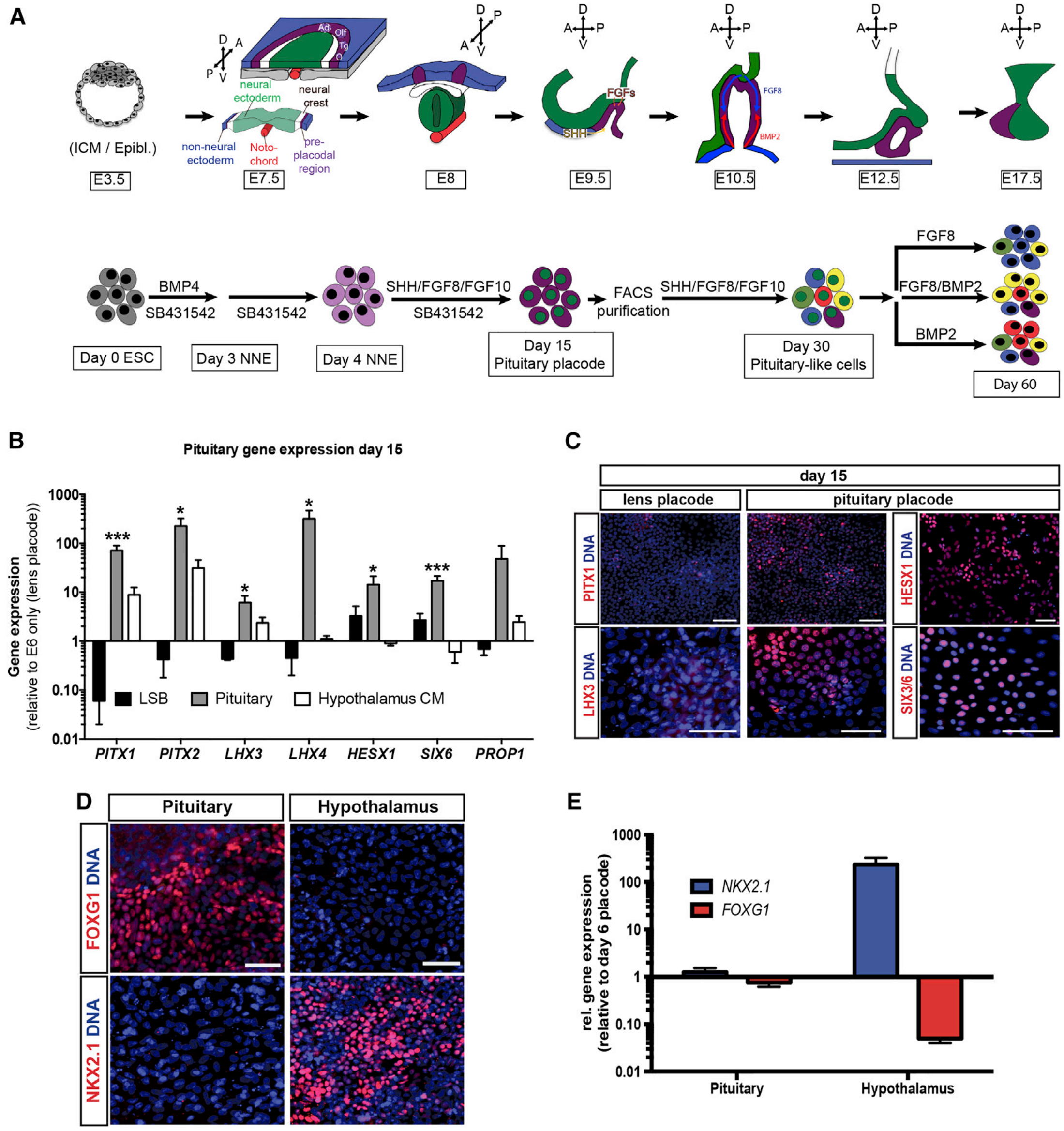

Figure 2. Pituitary Specification of Anterior Cranial Placode-Derived hPSCs

(A) Schematic representation of pituitary gland in vivo development and protocol for directed differentiation of hPSCs into anterior pituitary-like cells. ICM/Epibl., inner cell mass/epiblast; NNE, non-neural ectoderm.

(B) Real-time PCR analysis comparing expression of key genes involved in pituitary development in LSB, pituitary condition, and medium conditioned by hypothalamic neuroectoderm (Hypothalamus CM) after 15 days of differentiation in the respective medium. Values are normalized to *GAPDH* and gene expression on day 15 of lens differentiation (E6 only) and plotted as mean ± SEM of at least four independent experiments. $*p < 0.05$, $***p < 0.001$ compared with E6 only condition on day 15.

(C) Immunofluorescence analysis comparing expression of PITX1 and LHX3 after 15 days of differentiation under lens or pituitary conditions as well as expression of HESX1 and SIX3/6 on day 15 of pituitary differentiation. Scale bars, 50 μm.

(D) Immunofluorescence comparison of cells differentiated for 15 days under either pituitary or hypothalamus condition. Cells were stained for either FOXG1 (Pituitary) or NKX2.1 (Hypothalamus). Scale bars, 50 μm.

(E) qRT-PCR analysis of day 0-15 cells differentiated under pituitary or hypothalamic ectoderm condition probing for *NKX2.1* and *FOXG1*. Values have been normalized to *GAPDH* and expression in day-6 placode cells and are plotted as means ± SEM of two to four independent experiments.

See also Figures S2 and S3.

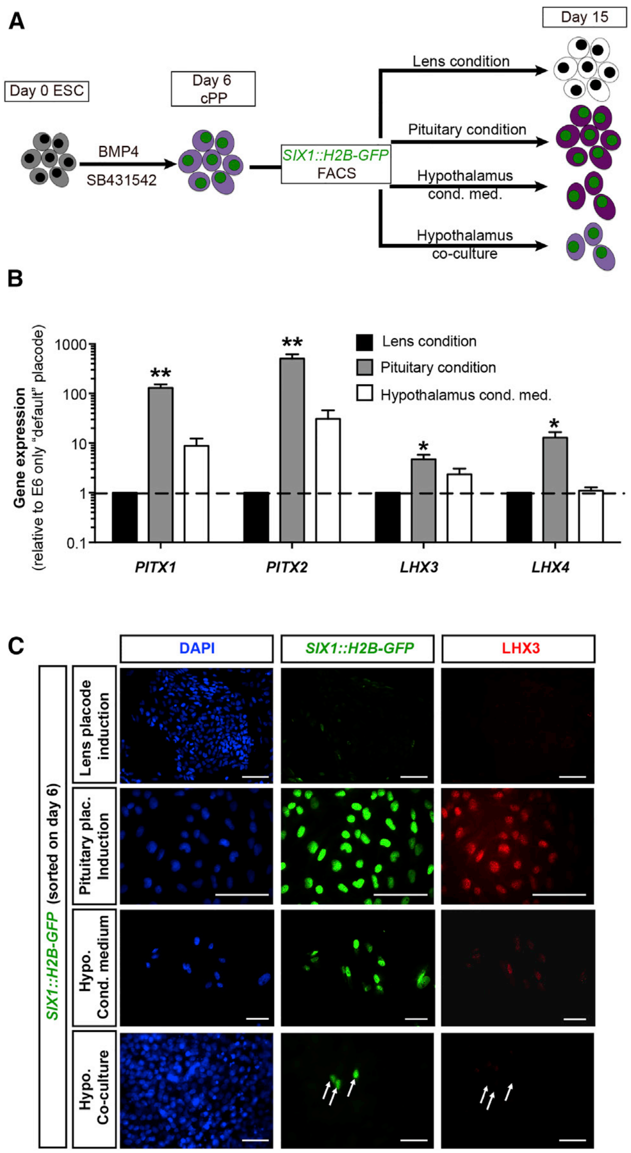

Figure 3. Pituitary Placode Induction from Purified SIX1:GFP-H2B Cells

(A) Schematic representation of the experimental outline. hESC were differentiated under default conditions for 6 days. Unpatterned SIX1$^+$ cells were FACS purified and cultured for an additional 9 days in various conditions. Cells were analyzed on day 15.

(B) Gene-expression analysis of key pituitary genes in cells grown in three conditions described in (A). Values are normalized to *GAPDH* and gene expression on day 15 of lens differentiation (E6 only) and plotted as mean ± SEM of at least four independent experiments. *$p < 0.05$, **$p < 0.01$ compared with E6-only condition on day 15.

(C) Immunofluorescence analysis of SIX1 sorted cells after 9 days of differentiation in respective medium condition. Arrows indicate absence of LHX3 expression in SIX1$^+$ cells in co-culture condition. Scale bars, 50 μm.

compensate for KSR lot-to-lot variability, which can dramatically affect differentiation efficiency, we performed PIP using two distinct concentrations of the BMP inhibitor LDN-193189. After 15 days of differentiation, the cells were analyzed using qRT-PCR probing for pan-placodal markers such as *SIX1* as well as the pan-pituitary markers *PITX1*, *PITX2*, *LHX3*, and *LHX4*. In addition, the neuroectoderm marker *PAX6* and the non-neural ectoderm transcription factor *TFAP2A* were included in the analysis (Figure S2A). Cell identity was further confirmed at the protein level using immunofluorescence staining for SIX1 and LHX3 (Figure S2B). The KSR lot used for these experiments failed to effectively induce pituitary or placode identity as shown by the low expression of *SIX1* and *TFAP2A* and high expression of *PAX6*. Lowering the LDN-193189 concentration was able to partially but not fully rescue that effect compared with our new E8/E6-based protocol. The cGMP-ready protocol presented here works reliably and with comparable efficiency across various hESC and hiPSC lines (Figures S3A–S3C). Furthermore, we confirmed that the recombinant protein SHH can be replaced by small-molecule smoothened agonists such as purmorphamine and SAG (Figures S3D and S3E). However, despite robust induction of anterior pituitary-lineage markers, we observed an increase in cell death when using the small-molecule-based induction conditions, which prompted us to use recombinant SHH for subsequent studies.

Interestingly, medium conditioned by hPSC-derived hypothalamic anlage (Maroof et al., 2013; Merkle et al., 2015) (Figures 2D and 2E) was not sufficient to robustly induce pituitary marker expression (such as *LHX4*, *HESX1*, and *SIX6*; Figure 2B). *LHX4* and *SIX6* have been implicated in pituitary progenitor expansion, which might explain the reduced cell yield using hypothalamic-conditioned medium. Despite compelling evidence to the contrary (Suga et al., 2011), our data suggest that defined extrinsic cues might be sufficient to induce pituitary placode identity. To directly assess the role of hypothalamic tissue during pituitary placode induction and differentiation, we made use of the *SIX1*::*H2B-GFP* reporter cell line. Early placode cells differentiated under default conditions were sorted at day 6 of differentiation for *SIX1*::*H2B-GFP* expression followed by further differentiation under either lens conditions (default), in the presence of SHH, FGF8, and FGF10 (pituitary conditions) or in the presence of medium conditioned by hPSC-derived hypothalamic neuroectoderm (Figure 3A). Gene-expression analysis revealed that even purified *SIX1*::*H2B-GFP*$^+$ cells, devoid of any hypothalamic lineage cells, are capable of expressing all key pituitary markers in response to defined cues. In contrast, hypothalamic conditioned medium failed to induce *LHX4* expression above levels observed under default lens conditions (Figure 3B). The levels of induction, especially *LHX4*,

were slightly lower in the *SIX1*::*H2B-GFP* purified cells compared with our standard pituitary PIP. This is possibly due to the relatively late pituitary induction, which started on day 6 (versus day 4) to exclude patterning of the pre-placode tissue. Alternatively, the sorting process or the associated cell dissociation may have also contributed to the slight decrease in induction efficiency.

In a separate set of experiments, we co-cultured day-6 sorted, *SIX1*::*H2B-GFP*$^+$ cells in direct contact with hPSC-derived hypothalamic anlage and stained the cells for the pan-placodal marker SIX1 and the pituitary marker LHX3 after 9 days of additional co-culture (day 15). As additional controls, we included the "default" lens conditions as well as our standard pituitary conditions and hypothalamic-conditioned medium (Figure 3A). While the lens condition formed lentoid-like clusters and downregulated SIX1 expression, the pituitary condition resulted in SIX1/LHX3 double-positive cells. The conditioned medium maintained SIX1 while inducing only low levels of LHX3. In the co-culture, SIX1 expression was maintained but no LHX3 expression was detected (Figure 3C). These findings support the idea that exposure to defined extrinsic cues is sufficient to direct hPSC into pituitary lineages cells. While we cannot rule out that further optimization of co-culture conditions may yield pituitary-lineage cells, we demonstrate that defined signals present a robust alternative that during cell manufacturing should greatly reduce variability inherent to complex co-culture systems.

hPSC-Derived Adenohypophyseal Cells Are Functional

The main function of the anterior pituitary (adenohypophysis) is to secrete six specific hormones controlling key events in the human body including stress response (adrenocorticotropic hormone [ACTH]), skeletal growth (growth hormone [GH]), metabolism (thyroid-stimulating hormone [TSH]), and reproductive functions (prolactin [PRL], follicle-stimulating hormone [FSH], luteinizing hormone [LH]). We therefore assessed the presence of hormonal subtypes in our culture. We were able to detect ACTH, GH, and PRL as well as FSH- and LH-expressing cells in our culture at day 30 of differentiation (Figure 4A). ELISA measurements of cell-culture supernatant confirmed basal secretion rates for ACTH, GH, and FSH (Figure 4B). Hormone release in the anterior pituitary gland is tightly regulated by several feedback mechanisms from the various target organs as well as from upstream hypothalamic signals delivered via the hypophyseal portal venous plexus. Therefore, the functional response of pituitary cells must integrate multiple, distinct regulatory stimuli. hPSC-derived pituitary cells at day 30 of differentiation showed induction of ACTH release in response to stimulation with corticotropin-releasing factor (CRF), stressin, or urocortin. In contrast, exposure to inappropriate stimuli such as ghrelin or somatocrinin did not trigger ACTH release (Figure 4C). On the other hand, somatocrinin but not CRF exposure triggered a significant increase in GH release (Figure 4D). Finally, the cultures also induced FSH release upon exposure to nafarelin (Figure 4E).

Single-Cell Gene-Expression Analysis Reveals Diversity of Hormonal Lineages

Differentiation of the hormonal progenitor lineages within the adenohypophysis (Tabar, 2011) is a tightly regulated spatial and temporal process involving various patterning events (Figure 2A). To address the diversity of progenitor fates in our hPSC-based culture system, we performed single-cell qRT-PCR analyses. We probed for 34 genes spanning pituitary development from undifferentiated PSCs to mature hormone-producing cells. We also included primers to survey for potential mesodermal or endodermal contaminants such as *T*/*Brachyury*, *MYOD*, and *SOX17*. Principal component analysis (PCA) of cells at day 30 and day 60 of differentiation showed a clear time-dependent change in expression with only few cells moving ahead of schedule (i.e., day-30 cells showing a day-60 profile) or being delayed (i.e., day-60 cells retaining a day-30 signature) (Figure 5A). The scree plot (Figure 5B) defined the PCA components that explain most of the variability of the data. Hierarchical clustering confirmed the separation of cells largely along the time axis, resulting in two main clusters interspersed with several smaller subclusters (Figure 5C). In addition, heatmaps based on the raw ct values are provided in Figure S4. We further validated our single-cell data by immunofluorescence staining in day-30 cultures for the progenitor marker HESX1, and for NEUROD1, a more mature marker transiently expressed in corticotrophs. Immunofluorescence analysis at day 15 of differentiation served as negative control for NEUROD1 (Figure S5A). We confirmed co-labeling of HESX1 and NEUROD1 in the same cell at day 30 of differentiation. However, the levels of HESX1 expression were much lower at day 30 compared with day 15.

Our analysis revealed that day-30 cultures contain an already high percentage of pituitary-like cells with ~70% of cells co-expressing pituitary transcripts such as *PITX1* and *LHX3*. Only four cells (~5% of all cells analyzed) expressed *T*, *SOX17*, or *MYOD*, suggesting a low percentage of contaminating cells. Most cells expressed *TBX19* (*TPIT*) a transcription factor shown to be crucial for the development of the *POMC* lineage (Lamolet et al., 2001). Furthermore, most cells expressed the pan-placodal marker *SIX1* and co-expressed *PAX6* compatible with pituitary placode fate. However, we also observed expression of other placode fates including *PAX2* (epibranchial), *PAX3* (trigeminal), or *PAX8* (otic) that together were detected in about 20% of the *SIX1*$^+$ population.

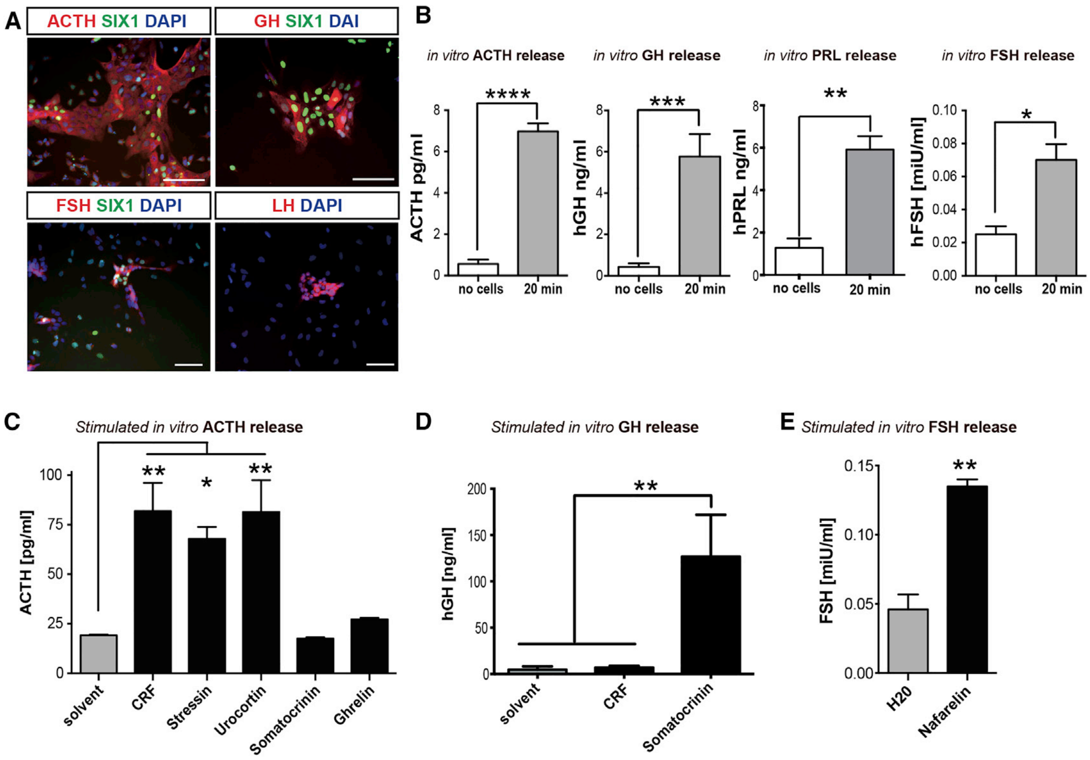

Figure 4. Functional Characterization of Anterior Pituitary Cells

(A) Immunofluorescence analysis of anterior pituitary cells after 30 days of differentiation. On day 30 the culture contains corticotrophs (ACTH), somatotrophs (GH), and gonadotrophs (FSH, LH). Scale bar, 50 μm.

(B) In vitro basal hormone release on day 30 of differentiation as assessed by ELISA. Data are plotted as mean ± SEM of three independent experiments. *$p < 0.05$, **$p < 0.01$, ***$p < 0.001$, ****$p < 0.0001$ compared with no cells (differentiation medium only).

(C–E) Quantification of hormone levels after 24 hr of in vitro stimulation using compounds triggering hormone release. ACTH release was specifically induced by CRF, stressin, or urocortin and not by somatocrinin or ghrelin (C), GH release was induced by somatocrinin but not CRF (D), and FSH release was induced by nafarelin (E). Data are plotted as mean ± SEM of three independent experiments. *$p < 0.05$, **$p < 0.01$ compared with the solvent control.

The ultimate functional units of the anterior pituitary are cells that secrete specific hormones. Our single-cell analysis showed that at day 30 of differentiation approximately 50% of the cells expressed at least one hormonal mRNA species. This percentage increased to about 80% by day 60, indicating further in vitro maturation (Figure 5D). This increase in the percentage of hormone-positive cells was one of the main factors responsible for the overall difference observed between day-30 and day-60 cells. There have been reports suggesting that both the developing and adult rodent pituitary gland contain cells that express more than a single hormone (Nunez et al., 2003; Villalobos et al., 2004). Indeed, in our hPSC-derived cultures we could detect expression of more than one hormonal transcript (hereafter termed "multiple hormone transcripts") in 10% of the cells by day 30 of differentiation. By day 60 of differentiation, this percentage increased to ~30% of the total cell population (Figure 5D). We found that the majority of multiple hormone transcript-expressing cells by day 60 expressed both *POMC* and *GH* (~10%). Cells expressing more than two hormone transcript types were only detected by day 60 and always included *POMC* expression (Figure S5B).

The most frequent hormonal transcript expressed in hPSC-derived pituitary cells at day 30 of differentiation was *POMC* (30% of total cells), thought to emerge from the dorsal pituitary anlage. The more ventral cell types such as *GH* or *TSH* made up about 20% of the total cell population by day 30 of differentiation. *PRL* was expressed in an even smaller subset of cells. Finally, *FSH* and *LH*, the two most ventral cell types, which appear only at later stages of development, were not detected by day 30 (Figure 5E). At day 60 of differentiation the number of *POMC*- and *GH*-expressing cells increased to 55% and 30%, respectively. Only few cells expressed *FSH* (~4%) and *LH* (~3%), even at day 60 of differentiation (Figure 5E). In addition to the single-cell PCR we characterized the cell-surface marker expression of the day-30 culture using

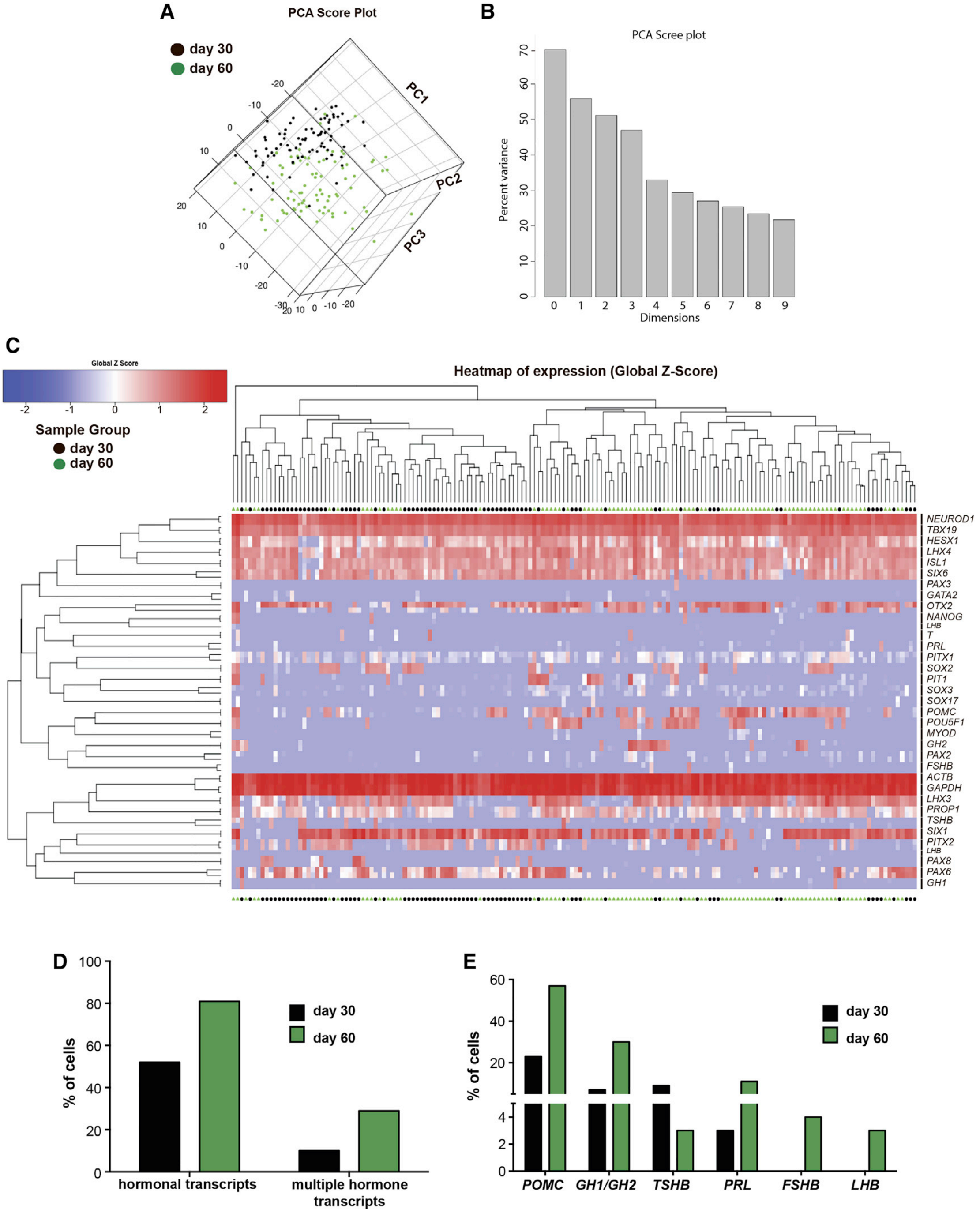

Figure 5. Temporal Single-Cell qRT-PCR Analysis of Anterior Pituitary Development In Vitro

(A and B) Principal component analysis of single cells on day 30 (black) and day 60 (green) of differentiation reveals two distinct populations of cells.

(C) Unsupervised hierarchical clustering of day-30 and day-60 cells using 34 different primer pairs identifies two clusters of cells with very few leading cells (day-30 cells resembling day-60 cells) and cells lagging behind (day-60 cells still more closely resembling day-30 cells).

(D) Quantification of hormone-expressing cells on day 30 and day 60 as well as percentage of cells expressing more than one hormonal transcript per cell.

(E) Expression of individual hormonal transcripts per single cell on day 30 and day 60, respectively.

See also Figures S4 and S5.

the commercially available BD Lyoplate screening kit (Figure S6).

Dorsal-Ventral Patterning of Anterior Pituitary Cells In Vitro Using Patterning Factors

Hypopituitarism is a very diverse and complex disease. Depending on the cause of pituitary dysfunction, the type of hormones affected can vary. For example, GH deficits are commonly observed in patients with inborn genetic disease (van Gelderen and van der Hoog, 1981) but can also occur in patients following radiation treatment (Sklar and Constine, 1995). In contrast, lymphocytic hypophysitis, an autoimmune disease of the pituitary gland, affects primarily ACTH (Rivera, 2006). Therefore, for the broad application of hPSC-derived pituitary cells in the future, cell replacement therapy may need to be customized to the specific needs of a given patient population. Since our standard conditions mostly yield dorsal, ACTH^{+} cells, we asked whether additional signals can be used to enhance the production of more ventral cell types. It has been shown that FGF8 and BMP2 signaling gradients play an important role in dorsal-ventral patterning of the mouse pituitary gland (Rosenfeld et al., 2000) (Figure 2A). We therefore treated pituitary-lineage cells with high concentrations of either FGF8 (dorsalizing) or BMP2 (ventralizing), or with a mixture of the two patterning factors at intermediate concentrations to mimic morphogen gradients occurring in vivo. Gene-expression studies for key transcription factors of pituitary precursor lineage and hormonal subtypes confirmed the need for BMP2 to generate the most ventral cell types. *FSHB* and *LHB* were significantly upregulated in the presence of BMP2 while FGF8 exerted a negative effect on *FSHB* yield (Figure 6A).

We next performed single-cell qRT-PCR analysis to increase the resolution of our analysis. Unsupervised hierarchical clustering of single cells at day 60, treated with FGF8, BMP2, or a combination of both factors, revealed three larger clusters of cells (Figure 6B). In addition, heatmaps based on the raw ct values are provided in Figure S4. Cells corresponding to each of the three treatments were observed in every cluster. However, 49% of all the total cells in cluster 3 were from the BMP2-treated group while 56% of all the cells in cluster 2 were derived from the FGF8 group. Cluster 1 represented cells from all three treatments in roughly equal proportions. By analyzing single-cell expression for each of the anterior pituitary hormones, we confirmed findings predicted from mouse in vivo studies (Rosenfeld et al., 2000) in our hPSC-based culture system. High concentrations of FGF8 led to increased numbers of *POMC*$^{+}$ cells compared with high concentrations of BMP2 (75% versus 40%, respectively) (Figure 6C). Furthermore, intermediate concentrations of both signaling molecules resulted in an increase of cells expressing *GH* and *TSHB* compared with either FGF8 or BMP2 alone (Figure 6C). Finally, high concentrations of BMP2 decreased the number of *POMC*$^{+}$ dorsal cell types and increased the number of ventral *FSHB* and *LHB* cell types.

Immunofluorescence confirmed the single-cell qRT-PCR data for four hormones under the various dorsal-ventral patterning conditions (FGF8, FGF8/BMP2, BMP2; Figure 6D). Quantification showed a bias toward dorsal ACTH (*POMC*)-expressing cells in the FGF8 treated culture. PRL and GH were the most abundant hormones observed upon patterning with FGF8/BMP2 while FSH was the most abundant cell type in BMP2-treated cultures (Figure 6E). POMC is the precursor polypeptide of ACTH and 44 amino acids are removed during translation, which ultimately gives rise to the hormone ACTH.

Grafted Human ESC-Derived Anterior Pituitary Cells Function in a Model of Hypopituitarism

To assess the ability of hPSC-derived pituitary cells to survive and function in vivo, we transplanted day-30 cells into hypophysectomized rats. After surgical removal of the pituitary gland (Figure 7A) using a parapharyngeal approach, hypopituitarism in the rats was confirmed by measuring ACTH levels in the plasma via ELISA. Rats that were successfully hypophysectomized were divided into two experimental groups, the sham group (n = 4) receiving Matrigel-only injections and the grafting group (n = 7) receiving Matrigel containing hESC-derived pituitary cells (day 30, standard conditions without BMP2 treatment). Following transplantation of 2×10^{6} cells subcutaneously, both treatment and control groups were followed for 7 weeks post-transplantation and pituitary hormone levels in the plasma were monitored (Figures 7B–7D). At 3 weeks after transplantation, hormone levels in the grafted group increased compared with the 1-week time point while the levels in the sham group remained largely unchanged. ACTH levels remained at higher levels in the grafted versus control group for the 7-week period of the experiment (Figure 7B). Increases in the level of two other hormones, namely GH and LH, were more variable and did not reach significance at all the time points tested. However, significant increases in GH levels were detected at 3 and 7 weeks after transplantation (Figure 7C). No significant increase in LH levels was observed (Figure 7D). Hormone levels in grafted animals were compared with levels in intact age-matched rats and found to be ~40% for ACTH, ~28% for GH, and ~20% for PRL (Figure S7). In a final step to evaluate the function of the transplanted cells, we performed measurements of the target hormones affected by pituitary hormone secretion. The normal HPA-axis response involves an increase in glucocorticoids secreted by the adrenal glands upon release of ACTH (Webster and Sternberg, 2004). In humans, ACTH triggers the release of cortisol,

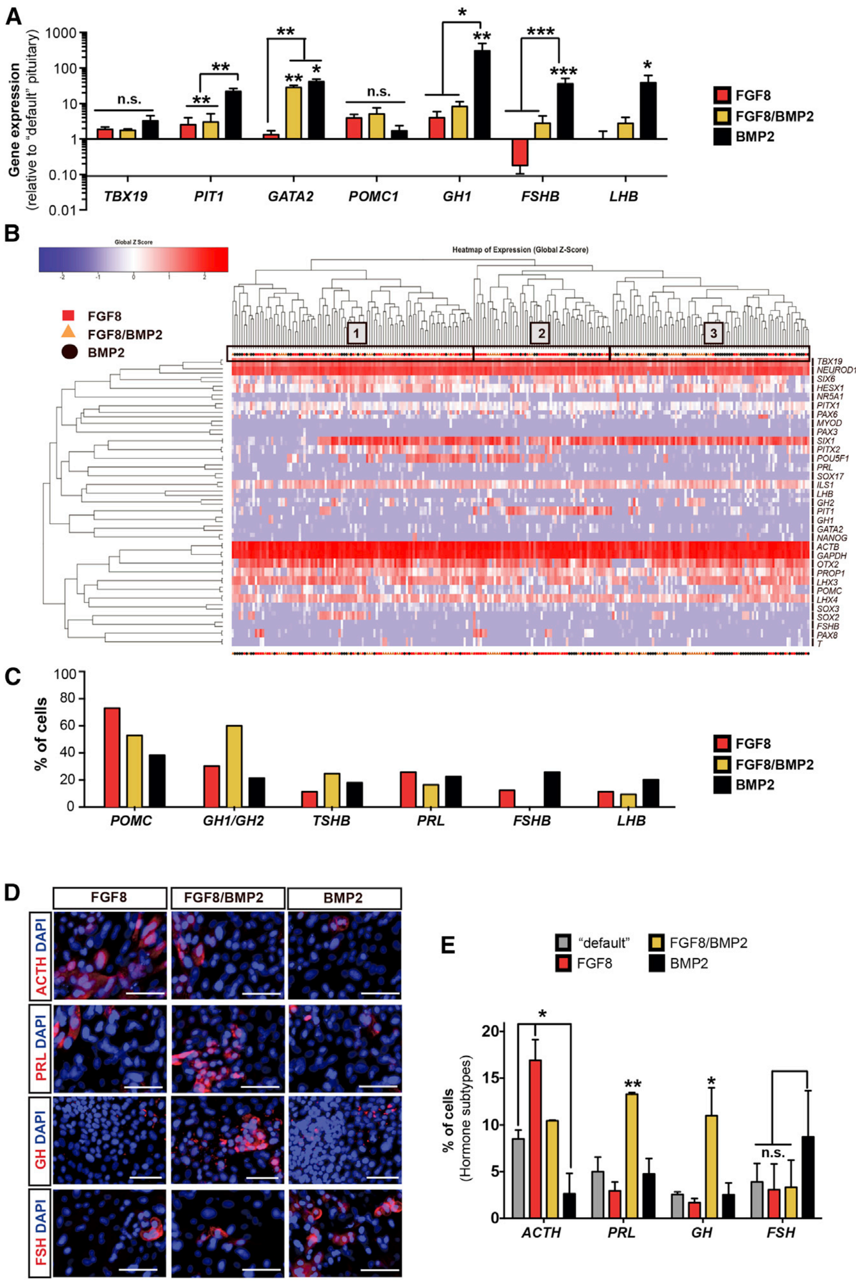

Figure 6. Specification of Hormonal Cells of the Pituitary In Vitro

(A) Bulk qRT-PCR analysis of day-60 cells patterned with FGF8, FGF8/BMP2, or BMP2 for 30 days. Patterning with BMP2 induced a more ventral cell identity (*PIT1*, *GATA2*, *GH1*, *FSHB*, and *LHB*) while FGF8 suppressed ventral cell types (FSHB). Data are plotted as mean ± SEM of two to four independent experiments. $^{*}p < 0.05$, $^{**}p < 0.01$, $^{***}p < 0.001$ (n.s., not significant) compared with the "default" pituitary differentiation on day 60.

(B) Unsupervised hierarchical clustering of FGF8, FGF8/BMP2, and BMP2 patterned cells using 34 primer pairs identified three larger clusters of cells, with cluster 2 mainly comprising cells patterned by FGF8 (or FGF8/BMP2) and cluster 3 mainly comprising cells patterned by BMP2 (or FGF8/BMP2).

(C) Quantification of hormonal transcripts per cell in different patterning conditions. Data are plotted as percentage of cells expressing the respective transcript (ct < 35 cycles in combination with a proper melting curve).

(D) Immunofluorescence analysis (representative images) of hormone expression in cells patterned with FGF8, FGF8/BMP2, or BMP2 on day 60 of differentiation. Scale bars, 50 μm.

(E) Quantification of hormone-expressing cells (per subtype) in different patterning conditions on day 60 of differentiation. High levels of FGF8 induced dorsal fate (ACTH) while intermediate levels of FGF8 and BMP2 induced dorsal/ventral fates (PRL and GH) compared with the default condition (E6 only). Data are plotted as mean ± SEM of two independent experiments. $^{*}p < 0.05$, $^{**}p < 0.01$ (n.s., not significant) compared with the "default" (E6 only) pituitary differentiation on day 60.
See also Figure S4.

whereas the main glucocorticoid in rodents is corticosterone (Wand, 2008). We therefore measured corticosterone levels in both experimental groups (Figure 7E). The grafted group showed consistently higher levels of corticosterone than sham-grafted animals resulting in a statistically significant difference by 7 weeks after transplantation. These data indicate an appropriate response by the host adrenal glands to human ACTH released by grafted hPSC-derived cells. At 7 weeks after transplantation, the animals were euthanized and the graft was analyzed histologically. We were able to detect cells expressing each of the six anterior pituitary hormones within the grafts (Figures 7F and 7G), confirming cell survival and suggesting, at least in part, further in vivo differentiation and maturation of the cells. Stereological quantification of the grafts showed an average of $3.08 \times 10^6 \pm 0.42 \times 10^6$ (average ± SD; n = 3) human cells per graft, with the majority (>95%) having a placode identity, as determined by co-expression of SIX1 and human nuclear antigen (hNA) by immunohistochemistry. The proportion of Ki67-positive proliferating cells in the graft was 9.6% ± 0.6% (average ± SD; n = 3) of the hNA^{+} population. The entire graft stained negative for

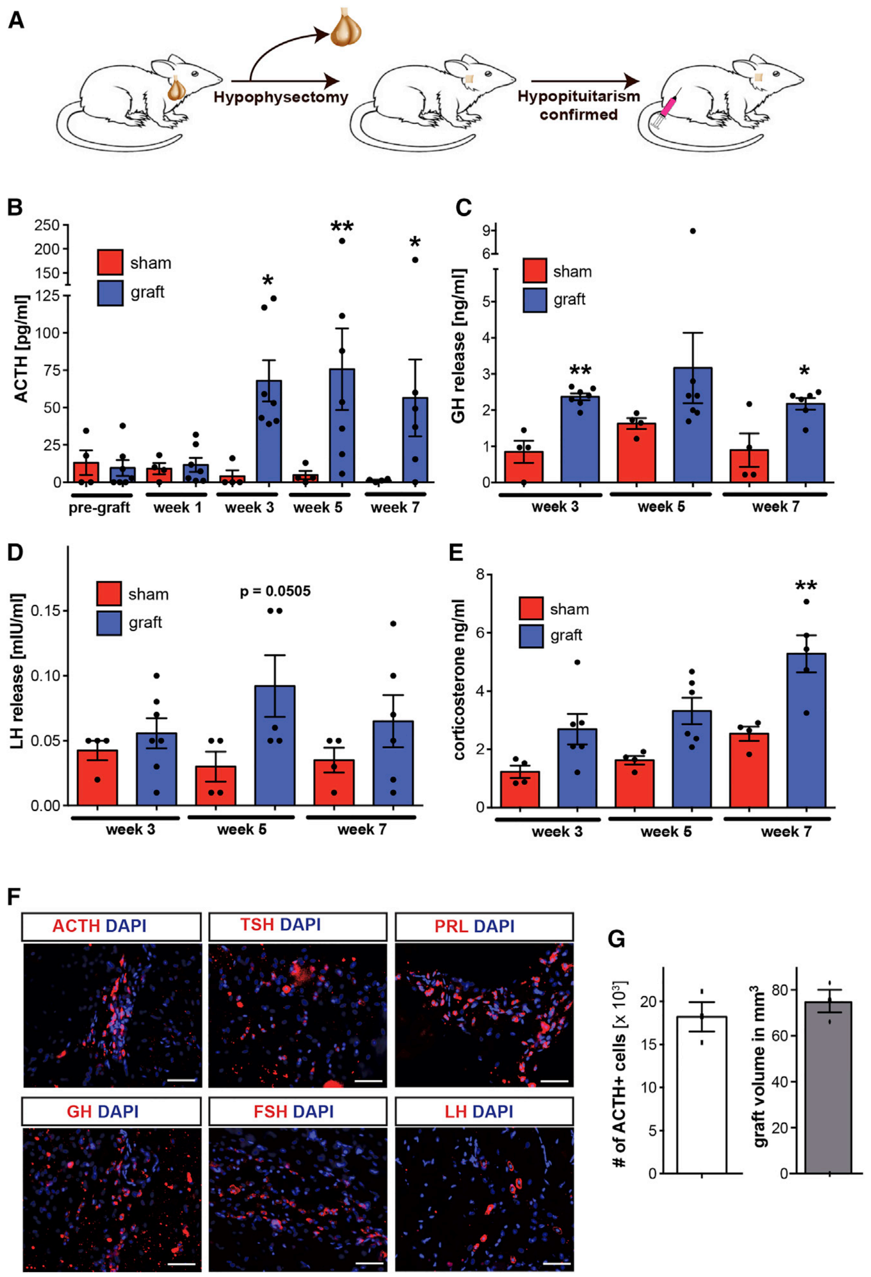

Figure 7. In Vivo Survival and Function of hPSC-Derived Anterior Pituitary Cells

(A) Schematic representation of experimental layout. After surgical removal of the pituitary gland and confirmation of hypopituitarism, cells embedded in Matrigel were transplanted subcutaneously.

(B–E) ACTH (B), GH (C), LH (D), and corticosterone (E) levels were quantified in plasma for up to 7 weeks after transplantation of the cells using ELISA. Data are plotted as mean ± SEM with each dot representing an individual animal. $^{*}p < 0.05$, $^{**}p < 0.01$ compared with the corresponding sham control.

(F) Immunohistological analysis of grafts 7 weeks after transplantation. Cells of each of the six hormonal lineages of the anterior pituitary gland were detectable within the graft. Scale bars, 50 μm.

(G) Quantification of cells expressing ACTH and the corresponding graft volume 7 weeks after transplantation. Data are plotted as mean ± SEM with each dot representing an individual animal (three animals in total).

See also Figure S7.

the pluripotency-associated surface markers SSEA-4 and Tra1-60. No signs of tumors were detected up to 7 weeks after transplantation. Stereological analyses showed an average of 18,212 ± 2,969 $ACTH^{+}$ cells per animal (optical fractionator method), and a graft volume of 81 ± 10 mm^3 (Cavalieri estimator; average ± SD, n = 3).

DISCUSSION

A goal of our study was the development of a defined and highly efficient protocol to generate anterior pituitary lineages that obviates the need for co-culture or complex media formulations and that should be suitable for clinical-grade cell manufacturing. Using fluorescence-activated cell sorting (FACS)-purified *SIX1::H2B-GFP*$^{+}$ placode cells, we demonstrate that pituitary fate can be induced at the expense of the default lens fate upon exposure to SHH, FGF8, and FGF10. Under these conditions, induction occurs in the absence of any hypothalamic cells previously thought to be critical for pituitary specification. While the signals essential for pituitary cell-fate induction from hPSCs do not require the presence of hypothalamic lineage

cells, the interaction with the hypothalamic anlage is likely important for the complex tissue movements characteristic of pituitary gland development such as the formation of the Rathke's pouch. In future studies it may be interesting to combine directed differentiation and organoid culture techniques to study developmental tissue interactions and to retain 3D cytoarchitecture. However, our current strategy offers obvious advantages for cell manufacturing of highly defined, therapeutically relevant cell types at scale. The cGMP-ready PIP presented here has several major advantages over the previously published protocol (Dincer et al., 2013). It is well established that KSR can exhibit considerable lot-to-lot variability (Rao, 2008; Schwartz et al., 2011). The protocol presented here overcomes these challenges and yields improved overall differentiation efficiencies under conditions of minimal lot-to-lot variability based on testing >10 different lots of Essential8 for hiPSC maintenance and >5 different lots of Essential6 for differentiation.

Our study made use of single-cell transcriptional profiling to define the heterogeneity of hormone-producing cells derived from hPSCs. The technology is a powerful tool that allowed us to determine the percentage of various hormone-producing cells and shifts in this distribution upon treatment with patterning factors or comparing different time points in vitro. However, one of the challenges of single-cell profiling is the largely binary readout of the expression data (McDavid et al., 2013). The HESX1/NEUROD1 validation experiment exemplifies this drawback, as the single-cell RT-PCR was not able to capture the decrease in HESX1 expression over time as observed by immunocytochemistry. Therefore, our binary approach of identifying expressed genes leads to the possibility that lowly expressed genes may be read as positive despite their expression levels not being high enough to achieve meaningful translation into protein. For example, we observed some instances of "inappropriate" expression patterns such as the co-expression of *SIX1* with *PAX6* and *PAX3* (though at different expression levels), which would indicate a mixed regional cranial placode identity. Similarly, the presence of putative plurihormonal cells was based purely on the expression of multiple hormonal transcripts. The presence of plurihormonal cells in the pituitary gland has been reported previously (Nunez et al., 2003; Villalobos et al., 2004) and is a well-known feature in pituitary adenomas (Scheithauer et al., 1986). However, it will be important to further assess in hPSC-derived pituitary cells whether transcripts for multiple hormones indeed result in the expression and secretion of multiple hormones from individual cells. To resolve some of the limitations of the single-cell gene-expression study, it will be important in the future to obtain appropriate positive and negative controls in order to define tissue-relevant thresholds of expression for each transcript. Unfortunately, developmentally matched human tissues are not readily available. In the present study we used immunofluorescence analysis as an independent method to quantify bona fide hormone-producing cells. Due to the lower sensitivity of this assay compared with qRT-PCR analysis, the percentages of hormone-positive cells were found to be lower but with the same relative proportions of the various hormone-producing cells.

We demonstrate that exposure to FGF8 and BMP2 can bias the dorsal-ventral composition of hormone-producing cells from $ACTH^+$ cells to FSH^+ or LH^+ cells. It should be noted that in vivo, cell types expressing different hormones are not necessarily located in specific areas but are scattered throughout the mature gland (Ericson et al., 1998; Olson et al., 2006). In addition, some reports argue for a less important role of extrinsic signaling compared with intrinsic factors in pituitary cell specification (Davis et al., 2011). Our human in vitro system provides a valuable tool to answer such questions. The current differentiation protocols only achieve partial enrichment of specific hormonal lineages. Therefore, lineage selection via cell sorting or the development of more sophisticated patterning strategies will be required to obtain purified populations such as GH^+ cells suitable for treating patients with selective GH deficiency (Bianchi et al., 2008). Our cell-surface epitope screen presents candidate markers that may be suitable for isolating specific hormone lineages. One cell type lacking in the current in vitro culture system are TSH^+ cells. Studies in sheep suggest that proper hypothalamic input is required for the development of thyrotrophs (Szarek et al., 2008). Therefore, it is conceivable that hypothalamic lineage cells may still be required for generating TSH^+ fates. Alternatively, TSH^+ cells may simply require further optimization of our defined induction conditions or longer in vitro differentiation periods.

The in vivo studies show clear evidence of survival and hormone release up to 7 weeks after transplantation. Whether the lower percentage of hormone-producing cells in vivo is due to limited cell survival or increased proliferation of immature pituitary progenitors remains to be determined. Future studies also need to define the optimal transplantation time point for in vivo yield and hormone subtype and demonstrate the long-term survival of hPSC-derived pituitary cells. One key question is whether the functional integration of human cells will eventually require orthotopic placement of the graft into the pituitary gland or hypothalamic region. Preliminary experiments with orthotopic (median eminence) placement of the cells into lesioned animals resulted in low cell survival, suggesting the need for further optimization of such a transplantation paradigm. Ectopic placement in accessible locations does offer advantages from a translational perspective, namely the ability to access and remove the graft, should

adverse side effects develop. However, hypothalamic release factors are the main triggers for hormonal release in the anterior pituitary gland (Smith and Vale, 2006). These release hormones have a short half-life and do not persist in the circulation. They are released into the hypophyseal portal venous plexus, which ensures immediate local delivery to the gland. Thus it is likely that the ectopically placed hPSC-derived grafts did not gain significant exposure to hypothalamic input. Our data therefore suggest a degree of autonomous hormonal release by the grafted cells, although a more comprehensive assessment of integration into homeostatic, endocrine mechanisms will be necessary. Based on existing literature on grafting primary cells (Maxwell et al., 1998; Naik et al., 1997), it is likely that proper regulation of at least some of the pituitary hormones will require orthotopic placement. Direct access to the skull base and pituitary gland in the mouse requires aggressive surgical procedures, such as transaural approaches or parapharyngeal neck dissection, complicating such studies. This is in marked contrast to human patients in whom surgical access to the pituitary gland can be safely performed through minimally invasive endoscopic transnasal routes (Tabar, 2011).

In conclusion, our study offers systematic access to human pituitary cell development and represents a powerful tool to obtain diverse hormone-producing cell types on demand and at scale. The platform also presents an important proof of concept in establishing functional and engraftable hormone-producing cells for regenerative medicine under conditions suitable for preclinical and clinical development.

EXPERIMENTAL PROCEDURES

Human Pluripotent Stem Cell Culture and Differentiation

The four hPSC lines (three hESC, one hiPSC) used in this study were maintained under feeder-free conditions using Essential8 and VTN-N. Cells were passaged twice a week using EDTA. All the differentiations carried out in this study are described in detail in Supplemental Experimental Procedures.

Phenotypic Characterization of Differentiated Cells

hPSC-derived cell types were characterized using immunofluorescence, qRT-PCR, flow cytometry, high content surface marker phenotyping (BD Lyoplate), and single hormone as well as multiplex ELISA. Details, including all antibodies used in this study, are described in Supplemental Experimental Procedures.

Single-Cell qRT-PCR

FACS-sorted single cells were captured using the C1 System (Fluidigm). Single-cell qRT-PCR was performed using wet-lab tested DELTAgene Assays (Fluidigm) in combination with EvaGreen chemistry using a BioMark System (Fluidigm). A complete list of primers used for this study is provided in Supplemental Experimental Procedures.

Animal Maintenance

The Animal Care and Use Committee at Memorial Sloan Kettering Cancer Center approved all animal protocols. All procedures were performed in accordance with NIH guidelines.

Statistical Analysis

All data are presented as mean ± SEM. The number of independent biological experiments performed for each analysis is indicated in the respective figure legend. Unpaired t test or one-way ANOVA with Bonferroni multiple-comparison post hoc test was used to assess significance levels. Significance levels were set at $^{*}p < 0.05$, $^{**}p < 0.01$, $^{***}p < 0.001$, and $^{***}p < 0.0001$.

SUPPLEMENTAL INFORMATION

Supplemental Information includes Supplemental Experimental Procedures, seven figures, and two tables and can be found with this article online at http://dx.doi.org/10.1016/j.stemcr.2016.05.005.

AUTHOR CONTRIBUTIONS

B.Z.: Conception and study design, hESC manipulation, differentiation and characterization, in vitro and in vivo analyses, data interpretation, and writing of manuscript. J.P.: transplantation studies. K.R.: technical support with single-cell PCR. M.J.T.: technical advice. V.T.: study design and data interpretation. L.S.: conception and study design, data analysis and interpretation, and writing of manuscript.

ACKNOWLEDGMENTS

B.Z. was supported by an NYSTEM post-doctoral training fellowship, The SKI Stem Cell Research Facility is supported by NYSTEM grants C029153 and C024175 and The Starr Foundation. The work was further supported in part by NIH/NCI grants R21CA176700-02 and P30CA008748. The authors thank M. Navare for technical support and A.F. Parlow and the National Hormone & Peptide program for the antisera against pituitary hormones, The PAX3 antibody developed by C.P. Ordahl, the TFAP2A antibody (3B5) developed by T.J. Williams, and the LHX3 antibody (Lim3, 67.4E12) developed by T.M. Jessell and S. Brenner-Morton were obtained from the Developmental Studies Hybridoma Bank, created by the NICHD of the NIH and maintained at the Department of Biology, University of Iowa, Iowa City.

Received: May 3, 2016
Revised: May 7, 2016
Accepted: May 10, 2016
Published: June 14, 2016

REFERENCES

Bailey, A.P., Bhattacharyya, S., Bronner-Fraser, M., and Streit, A. (2006). Lens specification is the ground state of all sensory

placodes, from which FGF promotes olfactory identity. Dev. Cell *11*, 505–517.

Bellin, M., Marchetto, M.C., Gage, F.H., and Mummery, C.L. (2012). Induced pluripotent stem cells: the new patient? Nat. Rev. Mol. Cell Biol. *13*, 713–726.

Bianchi, A., Giampietro, A., Pontecorvi, A., and De Marinis, L. (2008). Isolated growth hormone deficiency: clinical entity? J. Endocrinol. Invest. *31*, 12–15.

Chambers, S.M., Qi, Y., Mica, Y., Lee, G., Zhang, X.J., Niu, L., Bilsland, J., Cao, L., Stevens, E., Whiting, P., et al. (2012). Combined small-molecule inhibition accelerates developmental timing and converts human pluripotent stem cells into nociceptors. Nat. Biotechnol. *30*, 715–720.

Chemaitilly, W., and Sklar, C.A. (2010). Endocrine complications in long-term survivors of childhood cancers. Endocr. Relat. Cancer *17*, R141–R159.

Davis, S.W., Mortensen, A.H., and Camper, S.A. (2011). Birthdating studies reshape models for pituitary gland cell specification. Dev. Biol. *352*, 215–227.

Dincer, Z., Piao, J., Niu, L., Ganat, Y., Kriks, S., Zimmer, B., Shi, S.H., Tabar, V., and Studer, L. (2013). Specification of functional cranial placode derivatives from human pluripotent stem cells. Cell Rep. *5*, 1387–1402.

Dreser, N., Zimmer, B., Dietz, C., Sugis, E., Pallocca, G., Nyffeler, J., Meisig, J., Bluthgen, N., Berthold, M.R., Waldmann, T., et al. (2015). Grouping of histone deacetylase inhibitors and other toxicants disturbing neural crest migration by transcriptional profiling. Neurotoxicology *50*, 56–70.

Ericson, J., Norlin, S., Jessell, T.M., and Edlund, T. (1998). Integrated FGF and BMP signaling controls the progression of progenitor cell differentiation and the emergence of pattern in the embryonic anterior pituitary. Development *125*, 1005–1015.

Funato, K., Major, T., Lewis, P.W., Allis, C.D., and Tabar, V. (2014). Use of human embryonic stem cells to model pediatric gliomas with H3.3K27M histone mutation. Science *346*, 1529–1533.

Lamolet, B., Pulichino, A.M., Lamonerie, T., Gauthier, Y., Brue, T., Enjalbert, A., and Drouin, J. (2001). A pituitary cell-restricted T box factor, Tpit, activates POMC transcription in cooperation with Pitx homeoproteins. Cell *104*, 849–859.

Lee, G., Ramirez, C.N., Kim, H., Zeltner, N., Liu, B., Radu, C., Bhinder, B., Kim, Y.J., Choi, I.Y., Mukherjee-Clavin, B., et al. (2012). Large-scale screening using familial dysautonomia induced pluripotent stem cells identifies compounds that rescue IKBKAP expression. Nat. Biotechnol. *30*, 1244–1248.

Leung, A.W., Kent Morest, D., and Li, J.Y. (2013). Differential BMP signaling controls formation and differentiation of multipotent preplacodal ectoderm progenitors from human embryonic stem cells. Dev. Biol. *379*, 208–220.

Maroof, A.M., Keros, S., Tyson, J.A., Ying, S.W., Ganat, Y.M., Merkle, F.T., Liu, B., Goulburn, A., Stanley, E.G., Elefanty, A.G., et al. (2013). Directed differentiation and functional maturation of cortical interneurons from human embryonic stem cells. Cell Stem Cell *12*, 559–572.

Maxwell, M., Allegra, C., MacGillivray, J., Hsu, D.W., Hedley-Whyte, E.T., Riskind, P., Madsen, J.R., and Black, P.M. (1998). Functional transplantation of the rat pituitary gland. Neurosurgery *43*, 1157–1163.

McDavid, A., Finak, G., Chattopadyay, P.K., Dominguez, M., Lamoreaux, L., Ma, S.S., Roederer, M., and Gottardo, R. (2013). Data exploration, quality control and testing in single-cell qPCR-based gene expression experiments. Bioinformatics *29*, 461–467.

Merkle, F.T., and Eggan, K. (2013). Modeling human disease with pluripotent stem cells: from genome association to function. Cell Stem Cell *12*, 656–668.

Merkle, F.T., Maroof, A., Wataya, T., Sasai, Y., Studer, L., Eggan, K., and Schier, A.F. (2015). Generation of neuropeptidergic hypothalamic neurons from human pluripotent stem cells. Development *142*, 633–643.

Mica, Y., Lee, G., Chambers, S.M., Tomishima, M.J., and Studer, L. (2013). Modeling neural crest induction, melanocyte specification, and disease-related pigmentation defects in hESCs and patient-specific iPSCs. Cell Rep. *3*, 1140–1152.

Naik, D.R., Das, S., Patnaik, L., and Samantaray, H. (1997). A novel and simple technique for ectopic transplantation of the pituitary gland. Gen. Comp. Endocrinol. *107*, 304–310.

Nunez, L., Villalobos, C., Senovilla, L., and Garcia-Sancho, J. (2003). Multifunctional cells of mouse anterior pituitary reveal a striking sexual dimorphism. J. Physiol. *549*, 835–843.

Olson, L.E., Tollkuhn, J., Scafoglio, C., Krones, A., Zhang, J., Ohgi, K.A., Wu, W., Taketo, M.M., Kemler, R., Grosschedl, R., et al. (2006). Homeodomain-mediated beta-catenin-dependent switching events dictate cell-lineage determination. Cell *125*, 593–605.

Ozone, C., Suga, H., Eiraku, M., Kadoshima, T., Yonemura, S., Takata, N., Oiso, Y., Tsuji, T., and Sasai, Y. (2016). Functional anterior pituitary generated in self-organizing culture of human embryonic stem cells. Nat. Commun. *7*, 10351.

Pagliuca, F.W., Millman, J.R., Gurtler, M., Segel, M., Van Dervort, A., Ryu, J.H., Peterson, Q.P., Greiner, D., and Melton, D.A. (2014). Generation of functional human pancreatic beta cells in vitro. Cell *159*, 428–439.

Rao, M. (2008). Scalable human ES culture for therapeutic use: propagation, differentiation, genetic modification and regulatory issues. Gene Ther. *15*, 82–88.

Regal, M., Paramo, C., Sierra, S.M., and Garcia-Mayor, R.V. (2001). Prevalence and incidence of hypopituitarism in an adult Caucasian population in northwestern Spain. Clin. Endocrinol. *55*, 735–740.

Rezania, A., Bruin, J.E., Arora, P., Rubin, A., Batushansky, I., Asadi, A., O'Dwyer, S., Quiskamp, N., Mojibian, M., Albrecht, T., et al. (2014). Reversal of diabetes with insulin-producing cells derived in vitro from human pluripotent stem cells. Nat. Biotechnol. *32*, 1121–1133.

Rivera, J.A. (2006). Lymphocytic hypophysitis: disease spectrum and approach to diagnosis and therapy. Pituitary *9*, 35–45.

Rosenfeld, M.G., Briata, P., Dasen, J., Gleiberman, A.S., Kioussi, C., Lin, C., O'Connell, S.M., Ryan, A., Szeto, D.P., and Treier, M. (2000). Multistep signaling and transcriptional requirements for pituitary organogenesis in vivo. Recent Prog. Horm. Res. *55*, 1–13, [discussion: 13–4].

Scheithauer, B.W., Horvath, E., Kovacs, K., Laws, E.R., Jr., Randall, R.V., and Ryan, N. (1986). Plurihormonal pituitary adenomas. Semin. Diagn. Pathol. *3*, 69–82.

Schwartz, P.H., Brick, D.J., Nethercott, H.E., and Stover, A.E. (2011). Traditional human embryonic stem cell culture. Methods Mol. Biol. *767*, 107–123.

Schwartz, S.D., Regillo, C.D., Lam, B.L., Eliott, D., Rosenfeld, P.J., Gregori, N.Z., Hubschman, J.P., Davis, J.L., Heilwell, G., Spirn, M., et al. (2015). Human embryonic stem cell-derived retinal pigment epithelium in patients with age-related macular degeneration and Stargardt's macular dystrophy: follow-up of two open-label phase 1/2 studies. Lancet *385*, 509–516.

Sklar, C.A., and Constine, L.S. (1995). Chronic neuroendocrinological sequelae of radiation therapy. Int. J. Radiat. Oncol. Biol. Phys. *31*, 1113–1121.

Smith, J.C. (2004). Hormone replacement therapy in hypopituitarism. Expert Opin. Pharmacother. *5*, 1023–1031.

Smith, S.M., and Vale, W.W. (2006). The role of the hypothalamic-pituitary-adrenal axis in neuroendocrine responses to stress. Dialogues Clin. Neurosci. *8*, 383–395.

Suga, H., Kadoshima, T., Minaguchi, M., Ohgushi, M., Soen, M., Nakano, T., Takata, N., Wataya, T., Muguruma, K., Miyoshi, H., et al. (2011). Self-formation of functional adenohypophysis in three-dimensional culture. Nature *480*, 57–62.

Szarek, E., Farrand, K., McMillen, I.C., Young, I.R., Houghton, D., and Schwartz, J. (2008). Hypothalamic input is required for development of normal numbers of thyrotrophs and gonadotrophs, but not other anterior pituitary cells in late gestation sheep. J. Physiol. *586*, 1185–1194.

Tabar, V. (2011). Making a pituitary gland in a dish. Cell Stem Cell *9*, 490–491.

van Gelderen, H.H., and van der Hoog, C.E. (1981). Familial isolated growth hormone deficiency. Clin. Genet. *20*, 173–175.

Villalobos, C., Nunez, L., and Garcia-Sancho, J. (2004). Phenotypic characterization of multi-functional somatotropes, mammotropes and gonadotropes of the mouse anterior pituitary. Pflugers Archiv. *449*, 257–264.

Wand, G. (2008). The influence of stress on the transition from drug use to addiction. Alcohol Res. Health *31*, 119–136.

Webster, J.I., and Sternberg, E.M. (2004). Role of the hypothalamic-pituitary-adrenal axis, glucocorticoids and glucocorticoid receptors in toxic sequelae of exposure to bacterial and viral products. J. Endocrinol. *181*, 207–221.

Zhu, Z., and Huangfu, D. (2013). Human pluripotent stem cells: an emerging model in developmental biology. Development *140*, 705–717.

Zhu, X., Gleiberman, A.S., and Rosenfeld, M.G. (2007). Molecular physiology of pituitary development: signaling and transcriptional networks. Physiol. Rev. *87*, 933–963.

Zimmer, B., Lee, G., Balmer, N.V., Meganathan, K., Sachinidis, A., Studer, L., and Leist, M. (2012). Evaluation of developmental toxicants and signaling pathways in a functional test based on the migration of human neural crest cells. Environ. Health Perspect. *120*, 1116–1122.

Stem Cell Reports, Volume *6*

Supplemental Information

Derivation of Diverse Hormone-Releasing Pituitary Cells from Human Pluripotent Stem Cells

Bastian Zimmer, Jinghua Piao, Kiran Ramnarine, Mark J. Tomishima, Viviane Tabar, and Lorenz Studer

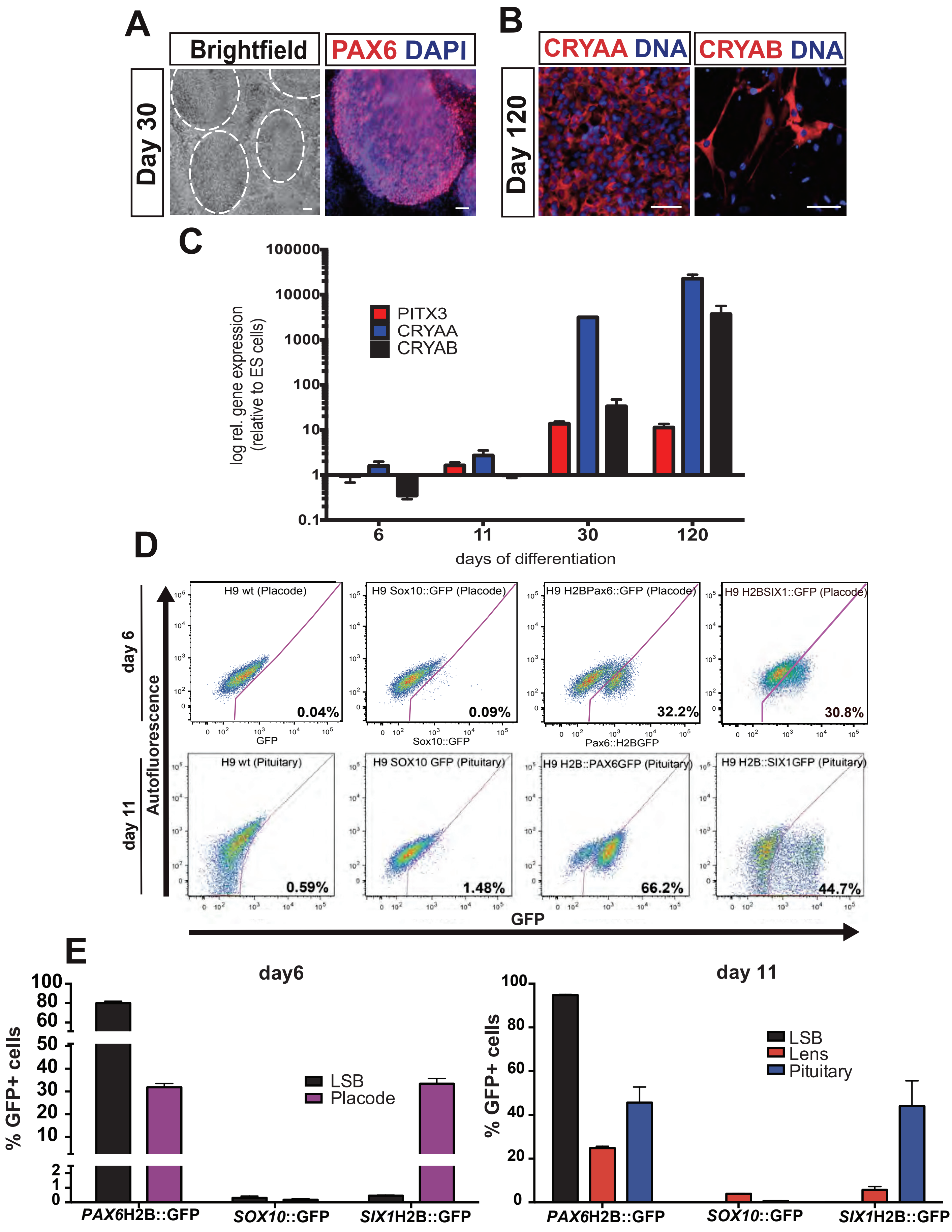
A
Brightfield
PAX6 DAPI
Day 30
B
CRYAA DNA
CRYAB DNA
Day 120
C
log rel. gene expression
(relative to ES cells)
100000
10000
1000
100
10
1
0.1
PITX3
CRYAA
CRYAB
6
11
30
120
days of differentiation
D
day 6
day 11
Autofluorescence
H9 wt (Placode)
0.04%
GFP
H9 Sox10::GFP (Placode)
0.09%
Sox10::GFP
H9 H2BPax6::GFP (Placode)
32.2%
Pax6::H2BGFP
H9 H2BSIX1::GFP (Placode)
30.8%
H9 wt (Pituitary)
0.59%
H9 SOX10 GFP (Pituitary)
1.48%
H9 H2B::PAX6GFP (Pituitary)
66.2%
H9 H2B::SIX1GFP (Pituitary)
44.7%
GFP
E
day6
% GFP+ cells
100
80
60
50
40
30
20
10
2
1
0
LSB
Placode
PAX6H2B::GFP
SOX10::GFP
SIX1H2B::GFP
day 11
% GFP+ cells
100
80
60
40
20
0
LSB
Lens
Pituitary
PAX6H2B::GFP
SOX10::GFP
SIX1H2B::GFP

Supplemental Figure 1: "Default" conditions in chemically defined media result in lens placode specification and quantification of ectodermal subtypes within the pituitary differentiation using reporter cell lines, related to Figure 1.

(A) After 30 days of differentiation under "default" conditions (E6 only) lentoid bodies (circled structures in brightfield image) staining positive for the lens marker PAX6 are clearly identifiable. Scale bars: 50 µm. (B) After an additional 90 days of differentiation (day 120) the majority of the cells is expressing crystalline, the predominant structural proteins in the lens. Scale bars: 50 µm. (C) qRT-PCR gene expression time course during lens differentiation. Cells differentiated for 120 days express the lens characteristic transcripts PITX3, CRYAA and CRYAB. Values have been normalized to GAPDH and expression in undifferentiated ES cells and are plotted as means +/- SEM of 4 independent consecutive experiments. (D) Cells (different reporter cell lines) differentiated for 6 and 11 days under either default placode or pituitary conditions were analyzed using Flow Cytometry for SOX10, PAX6 or SIX1 expression. Representative Flow Cytometry plots with percentages are shown. (E) Quantification of data form A) reveals very few contaminating neural crest cells (SOX10+) while confirming the anterior cranial placode character of the cells (SIX1+, PAX6+). Data is plotted as mean ± SEM of 2-8 independent experiments.

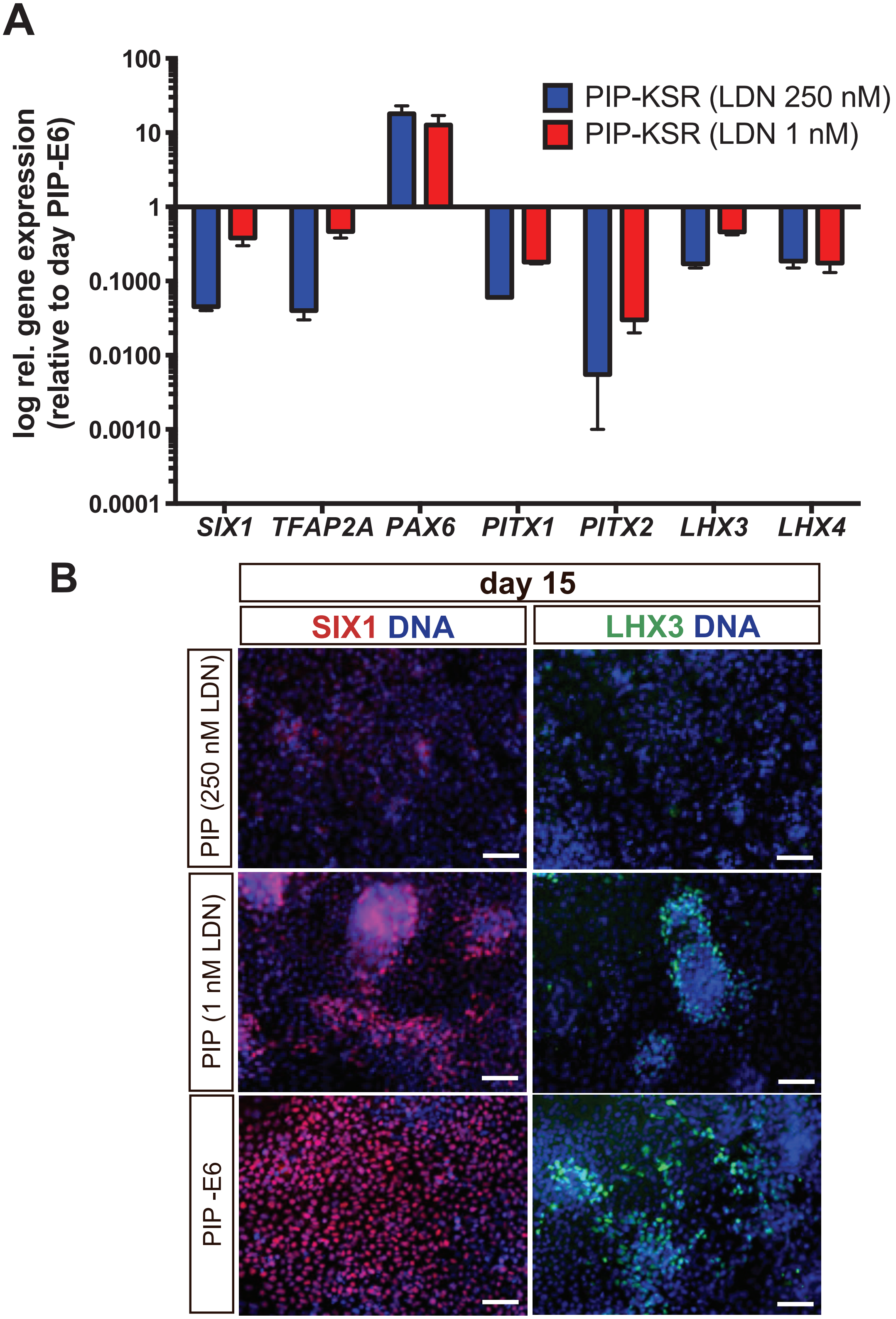

Supplemental Figure 2: Comparison of the traditional KSR-based pituitary induction with the new cGMP-ready induction, related to Figure 2.

Cells grown on feeders in KSR-based medium were differentiated using the old Dincer et. al. protocol (PIP-KSR). To compensate for KSR lot-to-lot variation 2 concentrations of LDN-193189 were used. Cells grown under feeder-free Essential8 conditions using the PIP-E6 protocol were differentiated in parallel. (A) qRT-PCR analysis of day 15 cells differentiated under PIP-KSR and PIP-E6 condition probing for SIX1, TFAP2A, PAX6, PITX1, PITX2, PITX3 and PITX4. Values have been normalized to GAPDH and expression in day 15 PIP-E6 cells and are plotted as means +/- SEM of 2 independent experiments. (B) Immunofluorescence comparison of cells differentiated for 15 days under either PIP-KSR or PIP-E6 condition. Cells were stained for either SIX1 (pan placode) or LHX3 (pan pituitary). Scale bars: 50 µm

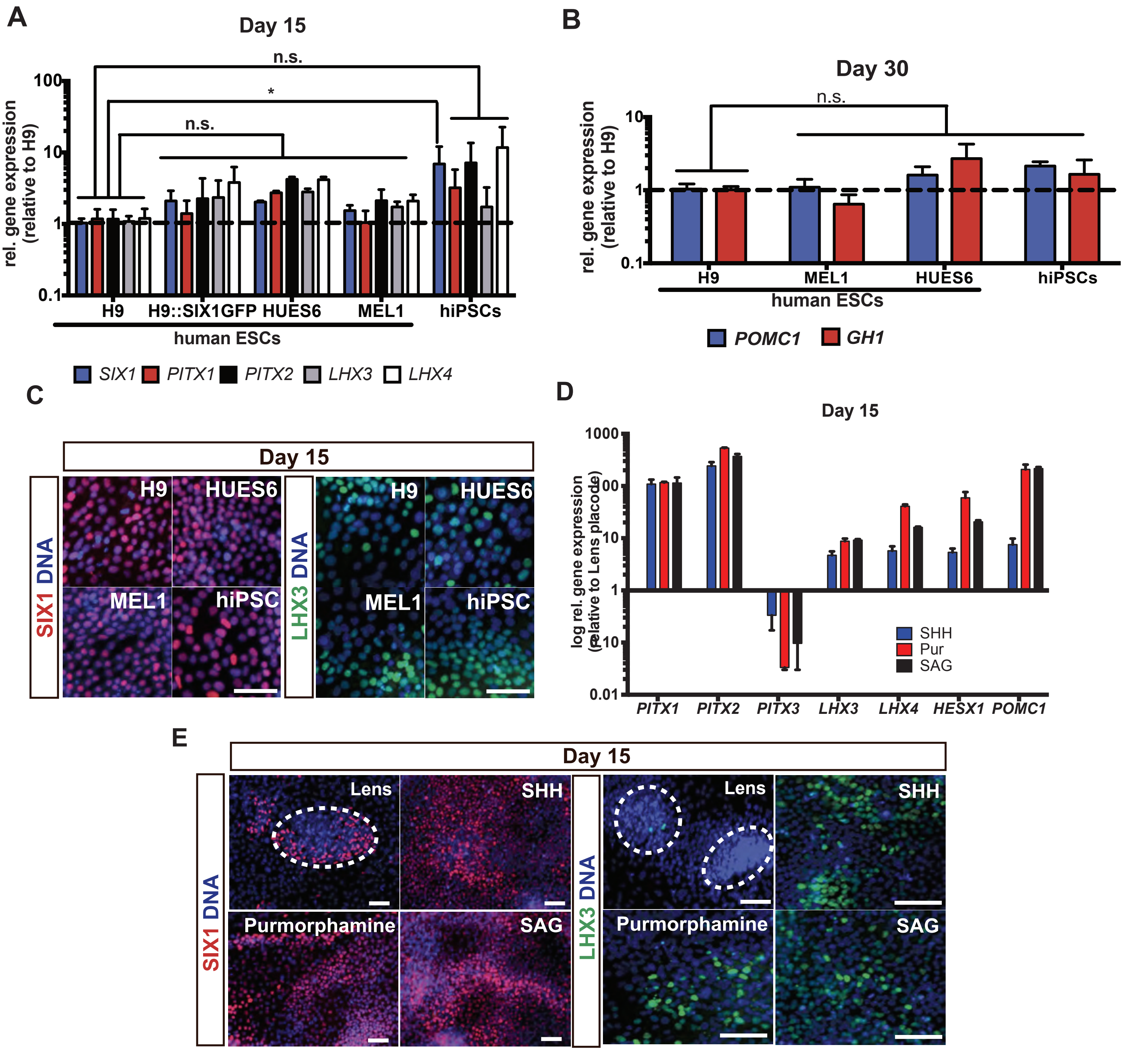

Supplemental Figure 3: Cell line comparison of pituitary induction protocol in E8/E6 and replacing recombinant SHH with small molecule smoothened agonists, related to Figure 2.

Four different hESC lines (including the H9 SIX1::H2B-GFP clone #6) and 1 hiPSC cell line were differentiated in parallel using the cGMP-ready pituitary induction protocol. (A) qRT-PCR analysis of day 15 cells differentiated under pituitary condition probing for the pan placodal marker SIX1 as well as the pan anterior pituitary genes PITX1, PITX2, LHX3 and LHX4. Values have been normalized to GAPDH and expression in day 30 wt H9 cells and are plotted as means +/- SEM of 2-4 independent experiments. (B) qRT-PCR analysis of day 30 cells differentiated under pituitary condition without sorting on day 15 probing for 2 anterior pituitary hormone transcripts POMC and GH1 Values have been normalized to GAPDH and expression in day 30 wt H9 cells and are plotted as means +/- SEM of 3 independent experiments. (C) Immunofluorescence analysis comparing protein expression on day 15 of pituitary placode induction across different hPSC lines. Scale bars: 50 µm. To investigate whether SHH can be replaced by small molecules, cells were differentiated using the cGMP-ready pituitary placode induction protocol using either recombinant SHH or one of the small molecule agonists purmorphamine or SAG in combination with FGF8 and FGF10. Lens placode differentiation was performed in parallel and served as a negative control. (D) qRT-PCR analysis of day 15 cells, differentiated under pituitary conditions using either SHH, purmorphamine or SAG from day 4 on, probing for the pan pituitary genes PITX1, PITX2, LHX3, LHX4, HESX1 as well as the hormone transcript POMC1 as well as the lens marker PITX3. Values have been normalized to GAPDH and expression in day 15 lens placode and are plotted as means +/- SEM of 3 independent experiments. (E) Immunofluorescence analysis comparing protein expression on day 15 of pituitary placode and lens induction using SHH and the small molecule alternatives purmorphamine and SAG. Early lentoid bodies (circled structures) start to downregulate expression of the pan placodal marker SIX1 while pituitary placode retains high SIX1 expression in combination with expression of LHX3. Scale bars: 50 µm

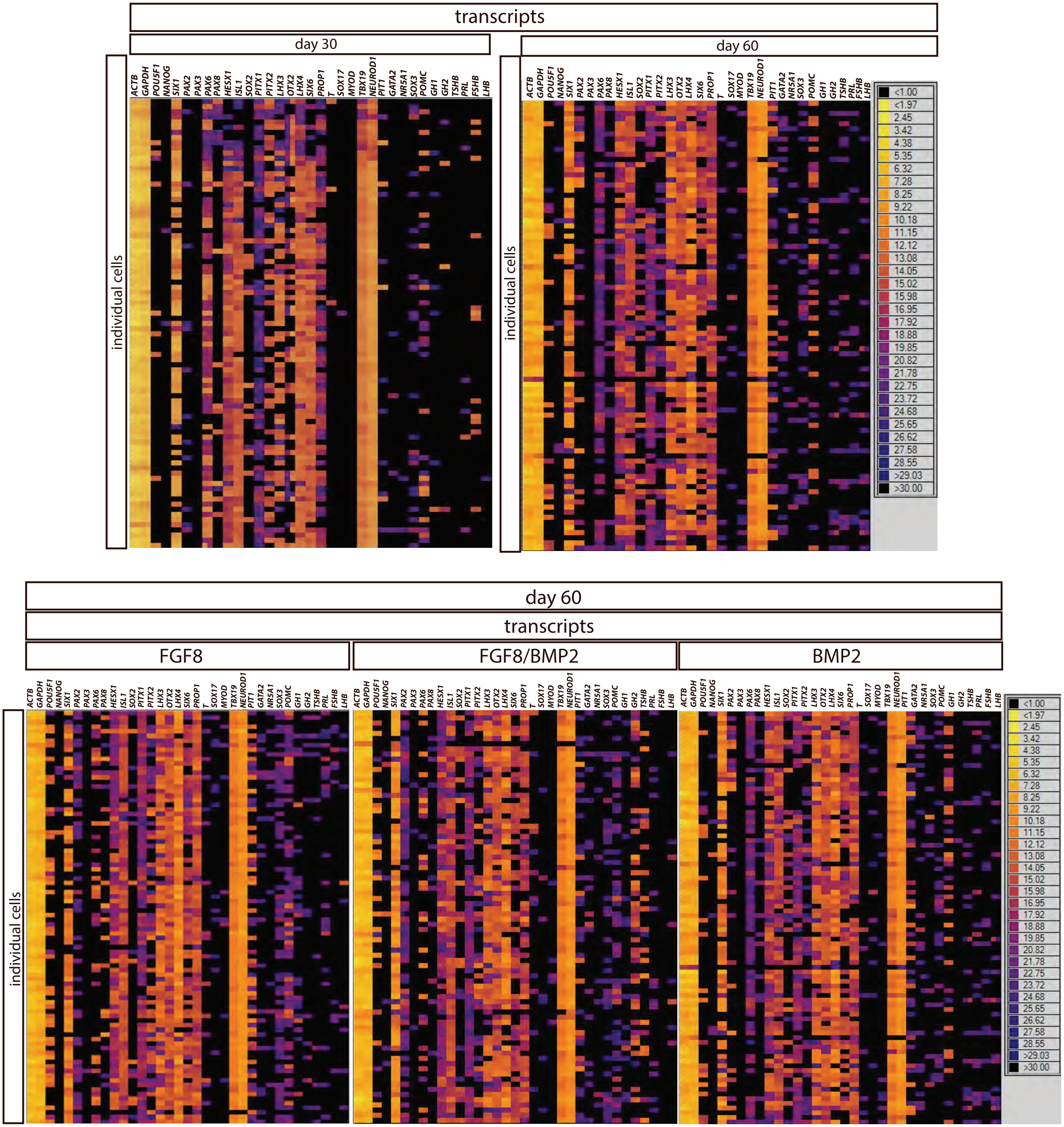

Supplemental Figure 4: Heatmaps of raw ct values for each cell and gene obtained by single cell q-RT PCR, related to Figure 5 and 6.

Raw ct values for every cell and gene obtained for every single cell PCR run are displayed as unprocessed heat maps.

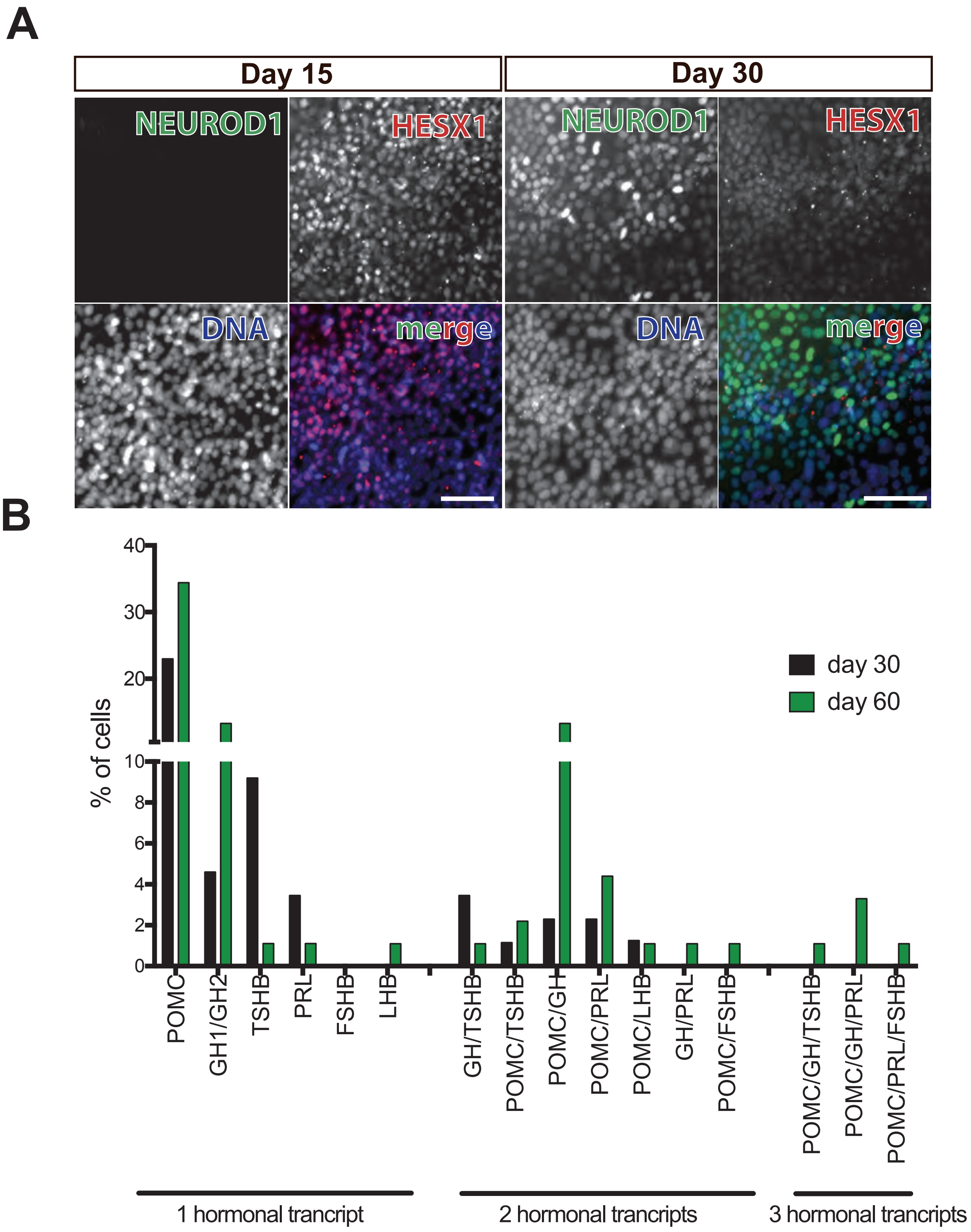

Supplemental Figure 5: Immunofluorescence validation of single cell q-RT PCR results and quantification of hormonal transcripts in single cells using single cell qRT-PCR, related to Figure 5.

(A) Immunofluorescence analysis of day 15 and day 30 cells differentiated under pituitary conditions. Cells were co-stained for the progenitor marker HESX1 and the transient cortiocotroph marker NEUROD1. Scale bars: 50 μm. (B) Single cell PCR data from day 30 and day 60 of the "default" pituitary differentiation protocol were mined for cells expressing at least one hormonal transcript. Data is plotted as percentage of cells expressing the respective transcript(s) (ct < 35 cycles in combination with a proper melting curve).

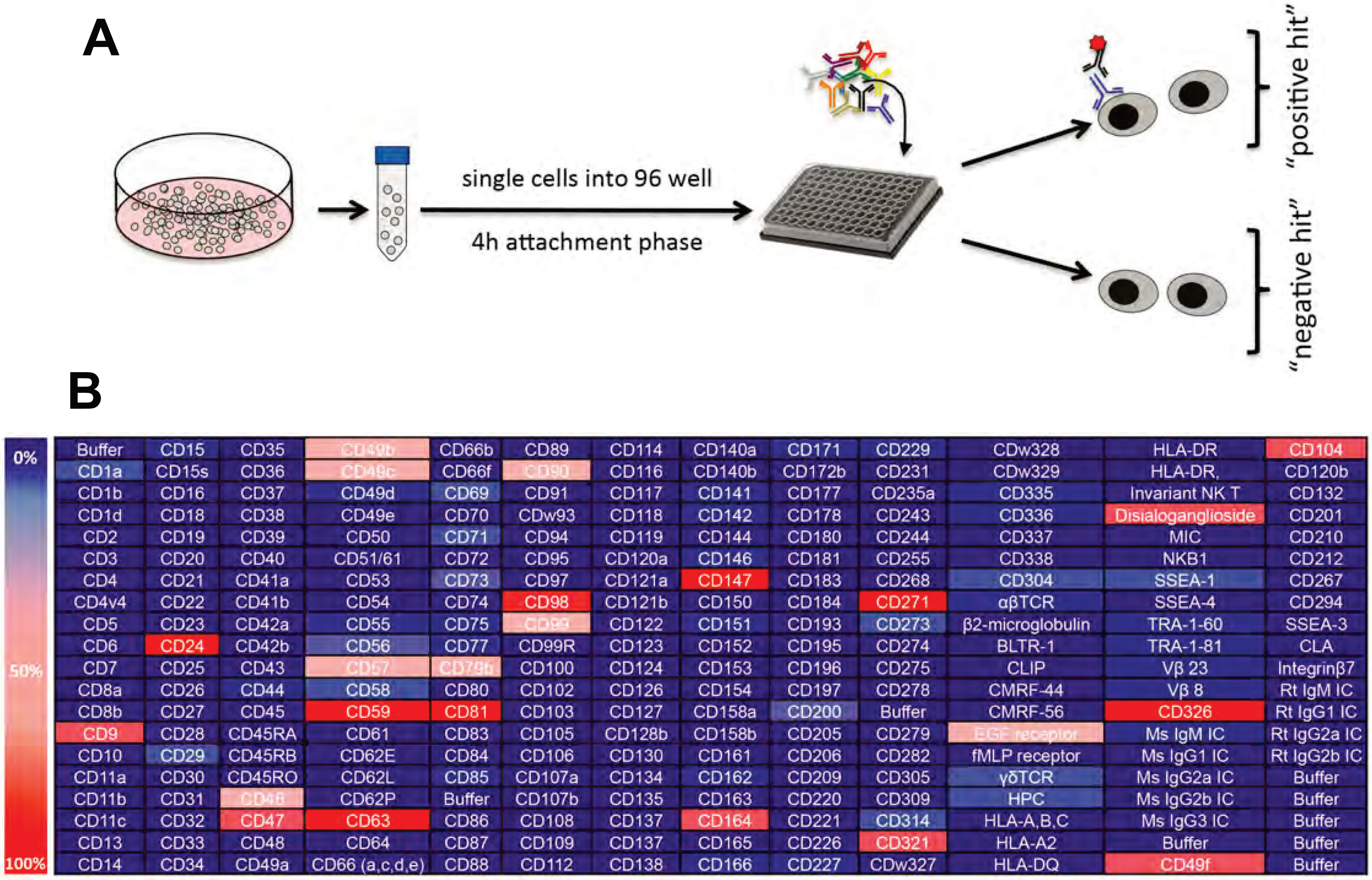

Supplemental Figure 6: Surface marker screen to identify hormonal subclass specific markers.

(A) Schematic representation of experimental procedure. (B) Heatmap of screen results. Percentage indicates cells staining positive for respective marker.

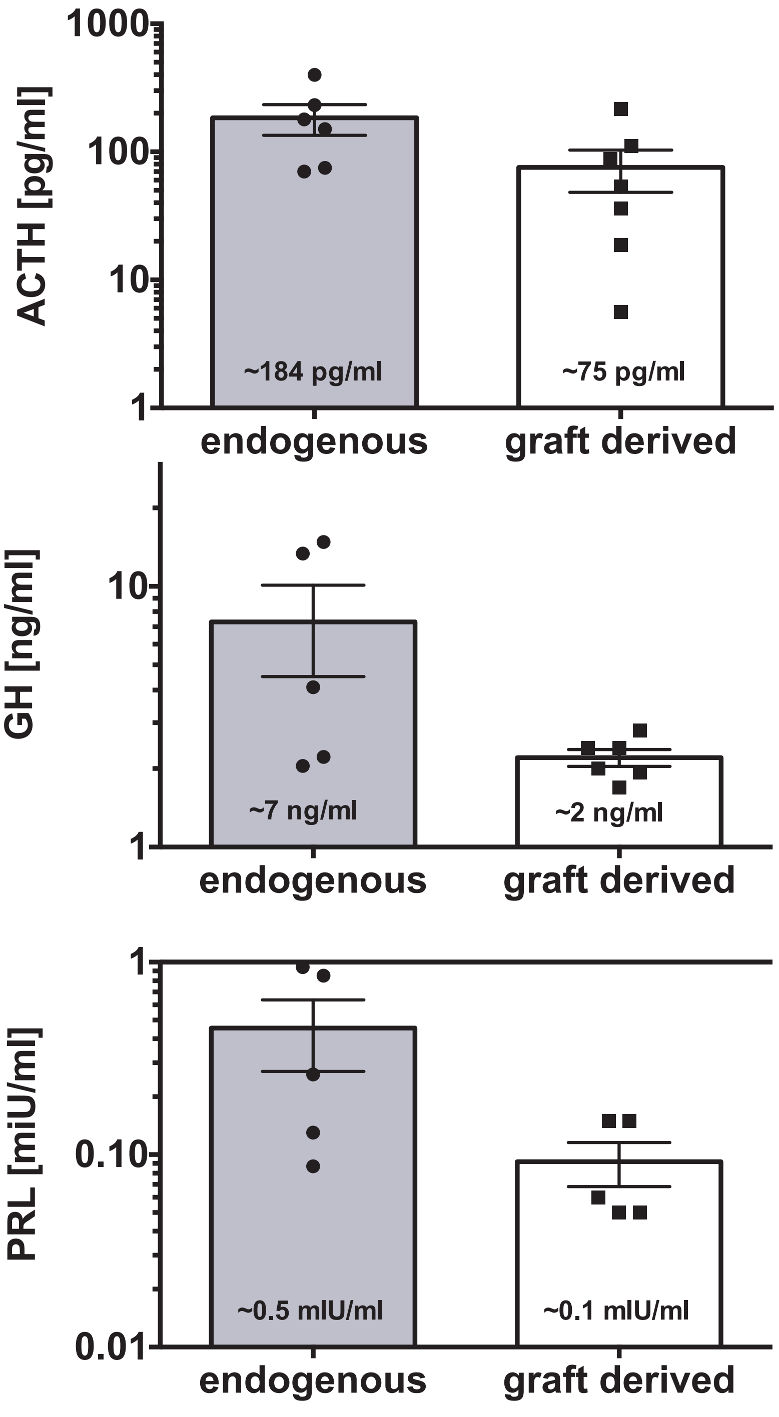

Supplemental Figure 7: Comparison of endogenous rat hormones in unlesioned animals and graft derived human hormones in lesioned animals, related to Figure 7.

Graft-derived hormone levels already presented in Main Figure 7 (week 5) are compared to endogenous hormone levels in unlesioned rats.

Supplementary Table S1: List of primers used in single cell qRT-PCR experiments

Target	FP	RP	Design RefSeq	Blast Hits	Gene Symbol	Gene Aliases
ACTB	CCAACCGCGAGAAGATGAC	TAGCACAGCCTGGATAGCAA	NM_001101.3	NM_001101	ACTB	
FSHB	GTCGTTTCTGCATAAGCATCA	CTGGCTGGGTCCTTATACAC	NM_000510.2	NM_001018080.1|NM_000510.2	FSHB	
GAPDH	GAACGGGAAGCTTGTCATCAA	ATCGCCCCACTTGATTTTGG	NM_002046.4	NM_002046|NM_001256799	GAPDH	
GATA2	GCCTGTGGCCTCTACTACAA	GTCTGGATCCCTTCCTTCTTCA	NM_001145661.1	NM_032638|NM_001145661|NM_001145662	GATA2	
GH1	ACTGGGCAGATCTTCAAGCA	GCAGCCCGTAGTTCTTGAGTA	NM_000515.3	NM_022560|NM_000515|NM_022559|NM_022562|NM_022561	GH1	
GH2	CCCGTCGCCTGTACCA	TGTTGGAATAGACTCTGAGAAGCA	NM_022557.3	NM_022558|NM_002059|NM_022557	GH2	
HESX1	GTTGGTATAGAGGCCGAAGACC	CCAGGATAGCAGTTTACTCTAAAGACA	NM_003865.2	NM_003865.2	HESX1	
ISL1	TCGCCTTGCAGAGTGACATA	CCCGGTCCTCCTTCTGAAAA	NM_002202.2	NM_002202.2	ISL1	
LHB	GCACCAAGGATGGAGATGC	GCTCCCTGGATGCCCAT	NM_000894.2	NM_000894	LHB	CGB4|hLHB|LSH-B
LHX3	TCGGACAAGGACAGCGTTCA	GCCCATTTCCGCCAAGGAA	NM_178138.3	NM_014564.2|NM_178138.3	LHX3	LIM3|CPHD3|M2-LHX3
LHX4	GGACTGTGGGGTTAGTGACA	CCCCACGTTGCCATAAATCC	NM_033343.2	NM_033343.2	LHX4	CPHD4
MYOD1	ACGAAGGCGCCTACTACA	GCAGTCTAGGCTCGACAC	NM_002478.4	NM_002478.4	MYOD1	
NANOG	TGCCTTGCTTTGAAGCATCC	TTTCTTCAGGCCCACAAATCAC	NM_024865.2	NM_024865	NANOG	
NEUROD1	GGCCCCAGGGTTATGAGACTA	ATCAGCCCACTCTCGCTGTA	NM_002500.4	NM_002500	NEUROD1	
OTX2	AGGAGGTGGCACTGAAAATCA	CTGTTGTTGGCGGCACTTA	NM_021728.2	NM_172337.1|NM_021728.2	OTX2	
PAX2	CGGCTGTGTCAGCAAAATCC	GCTTGGAGCCACCGATCA	NM_000278.3	NM_003989.3|NM_000278.3|NM_003987.3|NM_003988.3|NM_003990.3	PAX2	
PAX3	GCGGTCTGTGATCGAAACA	TCCTCCTCTTCACCTTTCCC	NM_181461.3	NM_181459.3|NM_001127366.2|NM_000438.5|NM_181458.3|NM_181457.3|NM_013942.4|NM_181460.3|NM_181461.3	PAX3	
PAX6	CCCCACATATGCAGACACACA	GAACTGACACACCAGGGGAAA	NM_001604.5	NM_001258465|NM_001258464|NM_001258463|NM_000280|NM_001604|NM_001127612|NM_001258462	PAX6	
PAX8	GCCCAGTGTCAGCTCCATTA	GCTGTCCATAGGGAGGTTGAA	NM_003466.3	NM_013992.3|NM_013953.3|NM_013952.3|NM_003466.3	PAX8	
PITX1	CCGTGTGGACCAACCTCA	GGTTACGCTCGCGCTTAC	NM_002653.4	NM_002653.4	PITX1	BFT|CCF|POTX|PTX1|LBNBG
PITX2	GCTGTGTGGACCAACCTTAC	CCCGAAGCCATTCTTGCATA	NM_000325.5	NM_000325.5|NM_153426.1|NM_153427.1	PITX2	
POMC	CTCACCACGGAAAGCAACC	CTGCTCGTCGCCATTTCC	NM_000939.2	NM_000939.2|NM_001035256.1	POMC	LPH|MSH|NPP|POC|ACTH|CLIP
POU1F1	AGGAACTCAGGCGGAAAAGTA	CAGGGCCTCCCCAACA	NM_000306.2	NM_000306|NM_001122757	POU1F1	PIT1|CPHD1|GHF-1|Pit-1|POU1F1a
POU5F1	GGTATTCAGCCAAACGACCAT	CCGCAGCTTACACATGTTCT	NM_002701.4	NM_001173531|NM_203289|NM_002701	POU5F1	
PRL	GGGCATGGAGCTGATAGTCA	TCCCGACCAGACAGGGTA	NM_000948.4	NM_000948.4|NM_001163558.1	PRL	
PROP1	AGTCAGCCTTTGGGAGGAAC	GTGAGCGCTCTTGCTTCC	NM_006261.4	NM_006261	PROP1	CPHD2|PROP-1
SIX1	AGAACCGGAGGCAAAGAGAC	CTGCTTGTTGGAGGAGGAGTTA	NM_005982.3	NM_005982	SIX1	
SIX6	AGGTGGGCAACTGGTTCA	CCCGGAACCCTGTGAC	NM_007374.2	NM_007374	SIX6	Six9|OPTX2|MCOPCT2
SOX17	CACAACGCCGAGTTGAGCAA	GCTCTGCCTCCTCCACGAA	NM_022454.3	NM_022454.3	SOX17	
SOX2	CATGAAGGAGCACCCGGATTA	CGGGCAGCGTGTACTTATCC	NM_003106.2	NM_003106.2	SOX2	
SOX3	GTGTGAAACGGCCCATGAAC	GTGCATCTTGGGGTTCTCCA	NM_005634.2	NM_005634	SOX3	PHP|GHDX|MRGH|PHPX|SOXB
T	CGCTTCAAGGAGCTCACCAA	GCCAGACACGTTCACCTTCA	NM_003181.2	NM_003181.2	T	
TBX19	ACCCAGTTCATAGCCGTGAC	AGGCTTTGGCAAAAGGATTGTAC	NM_005149.2	NM_005149.2	TBX19	TPIT|TBS19|dJ747L4.1
TSHB	AATACCAGGATGCCCACTCC	GTATTGCACTTGCCACACTTACA	NM_000549.4	NM_001277991|NM_000549	TSHB	TSH-B|TSH-BETA

Supplementary Table S2. Antibodies used in this study, related to Figure 1, 2, 3, 4, 6, 7, S1, S2, S3 and S5

Antigen	Company	Catalogue #	dilution
ACTH	NIDDK	hACTH-IC	1:100
CRYAA	OriGene	CF505577	1:100
CRYAB	OriGene	CF500680	1:100
DLX3	Santa Cruz	Sc-98522	1:100
ECAD	BD Pharmingen	560062	1:200
EYA1	Avia Systems Biology	ARP32434_P050	1:200
FOXG1	StemCulture	NCFAB	1:1000
FSH	NIDDK	hBetaFSH-IC-3	1:100
GFP	Abcam	Ab13970	1:1000
GH	NIDDK	hGH-IC-2	1:100
HESX1	Sigma	HPA024187	1:500
hNA	Millipore	MAB1281	1:100
Ki67	DAKO	M7240	1:100
LH	NIDDK	hBetaLH-IC-3	1:100
LHX3 (rabbit)	Millipore	AB3202	1:300
LHX3 (mouse)	DSHB	67.4E12	1:100
NKX2.1 (TTF1)	Thermo Scientific	MS-699	1:200
NEUROD1	BD Pharmingen	563000	1:300
OCT4	Cell Signaling	2840	1:400
PAX3	DSHB	Pax3	1:100
PAX6	Covance	PRB-278P	1:800
PITX1	Sigma	HPA008743	1:500
PRL	NIDDK	hPRL-IC-5	1:100
SIX1	Sigma	HPA001893	1:500 -1:1000
SIX3/6	Santa Cruz	sc-9126 X	1:300
SOX10	Santa Cruz	sc-17342	1:100
SOX2	Cell Signaling	3579	1:400
TFAP2A	DSHB	3B5	1:100
TSH	NIDDK	hBetaTSH-IC-2	1:100

Supplemental experimental procedures

ESCs and culture conditions

The human pluripotent stem cells H9 (WA-09, XX, passage 35-50), MEL-1 (XY, passage 20-40), HUES-6 (XX, passage 24-40), hiPSCs (in-house generated hiPSCs derived from the fetal fibroblast cell line MRC5 (ATCC CCL-171) (Chambers et al., 2009), XY, passage 15-30) and modified reporter cell lines (all H9 background, passage 40-75) were maintained on VTN-N (Fisher Scientific) using Essential8 medium (E8) (Fisher Scientific) (Chen et al., 2011) and passaged twice a week using EDTA (Chen, 2008). Cells were tested for mycoplasma contamination once a month.

ESC differentiation

Differentiation into neural ectoderm was performed as previously described (Chambers et al., 2009) with slight modifications. Briefly, cells were plated at 250 000 cells/cm^2 on VTN-N coated dishes in E8 + Y-27632 (Tocris). After 24h (day0) medium was changed to Essential6 (E6) (Fisher Scientific) supplemented with 10 μM SB431542 (Tocris Biosciences), 500 nM LDN193189 (Stem Cell Technologies) and 1μM XAV939 (Tocris Biosciences) (until day 5). From day 5 on XAV939 was removed from the medium. Medium was changed every day until day 11.

Hypothalamic ectoderm differentiation was performed as described earlier (Maroof et al., 2013; Merkle et al., 2015) with slight modifications. Briefly, cells were plated at 250,000 cells/cm^2 on VTN-N coated dishes in E8 + Y-27632. After 24h (day0) medium was changed to E6 supplemented with 10 μM SB431542 for 2 days. From day 2 on E6 medium was supplemented with high concentrations of SHH (1 μg/ml) until day 11. For conditioned medium preparation cells were cultured for 24h in E6 only and washed twice afterwards to remove potential SHH from the induction medium. On day 13 E6 only was added to the cells and conditioned for 24h. Prior to using it, the conditioned medium was sterile filtered to eliminate debris and dead cells.

For lens differentiation cells were plated at 250 000 cells/cm^2 on VTN-N coated dishes in E8 + 10 μM Y-27632. After 24h (day0) medium was changed to E6 supplemented with 10 μM SB431542 and 5 ng/ml BMP4 (R&D Systems). Medium was changed every day. On day 3 BMP4 was removed from the medium and cells were cultured in E6 + 10 μM SB431542 until day 15. From day 15 on cells were maintained in E6 only for up to 120 days. From day 30 on, medium was supplemented with VTN-N (1:100) once a week during feeding to prevent cells from peeling of the plate.

For pituitary differentiation cells were plated at 250 000 cells/cm^2 on VTN-N coated dishes (differentiation works best in 24 well plates) in E8 + 10 μM Y-27632. After 24h (day0) medium was changed to E6 supplemented with 10 μM SB431542 and 5 ng/ml BMP4. Medium was changed every day. On day 3 BMP4 was removed from the medium and cells were cultured for 1 day in E6 + 10 μM SB431542. For the standard differentiation conditions, E6 was supplemented with 10 μM SB431542, 200 ng/ml SHH (R&D Systems, C25II), 100 ng/ml FGF8b (R&D Systems) and 50 ng/ml FGF10 (Peprotech Inc.) on day 4. For some experiments SHH was replaced by 1 μM purmorphamine (Stemgent) or 1 μM SAG (Stemcell Technologies) From now on medium volume was doubled and cells were feed every other day until day 15. On day 15 of differentiation *SIX1::H2B-GFP*$^+$ cells were sorted using a BDFACS Aria III cell sorter. Purified cells were then plated as droplets (50,000 cells/10 μl drop) in E6 supplemented with 10 μM Y-27632, 200 ng/ml SHH, 100 ng/ml FGF8b and 50 ng/ml FGF10 on poly-ornithine/laminin/fibronectin-coated plates. After 24h medium was changed to E6 containing SHH, FGF8 and FGF10 until day 30. Medium was changed every other day. For some experiments pituitary induction was started slightly later (day 6) or cells were differentiated in medium conditioned by hypothalamic neuroectoderm from either day 4 or day 6 on. For the co-culture experiment *SIX1::H2B-GFP* positive cells were sorted on day 6 and 50,000 cells/cm^2 were plated directly on hypothalamic neuroectodermal cells in E6 only supplemented with 10 μM SB431542.

Pituitary cell maturation and subtype specification

Unless otherwise stated in the text, standard pituitary maturation medium on day 30 was changed to "E6 only" for additional 30 days. For patterning experiments (indicated in the text) E6 medium was supplemented with high concentrations of either FGF8 (100 ng/ml, dorsalize) or BMP2 (20 ng/ml, ventralize) or intermediate concentrations of both (FGF8 50 ng/ml, BMP2 10 ng/ml).

RNA extraction and traditional quantitative Real-time PCR

Total RNA was extracted from at least 3 independent experiments using the TRIzol (Fisher Scientific) reagent in combination with Phase-lock tubes (5Prime) according to the manufactures protocol. 1 µg of total RNA was reverse transcribed into cDNA using iScript (BioRad). For quantitative RT-PCR we used the SSoFast EvaGreen Mix (BioRad) in combination with QuantiTect primer assays (Qiagen) on a BioRad CFX96 Thermal Cycler. All reactions were run according to the manufacturer's protocol. Gene expression was normalized to glyceraldehyde 3-phosphate dehydrogenase (*GAPDH*) and a control cell type (indicated in the Figures). Results are calculated using the ΔΔCt method (Livak and Schmittgen, 2001).

Single Cell quantitative RT-PCR

For single cell PCR analysis cells were detached using Accutase. After filtering through a 40 µm cell strainer DAPI negative cells were sorted on a BDFACS Aria III machine to select for live cells. Sorted cell suspension was adjusted to a concentration of 400,000 cells/ml. Single cells were captured using the Fluidigm C1 system according to manufacturer's protocol. Capture rate of for each C1 chip (C1 Single-cell Auto Prep Array IFC (10 -17 µm)) was confirmed microscopically using a standard tissue culture brightfield microscope. Capture rates were as follows: day 30: 91% (87/96) day 60: 94% (90/96) day 60 FGF8: 93% (89/96) day 60 FGF8/BMP2: 89% (85/96) day 60 BMP2: 93% (89/96). Cells were lysed, RNA was extracted and transcribed into cDNA and pre-amplified using the C1 in combination with wet-lab tested Fluidigm DELTAgene assays following the manufacturer's protocol. The resulting cDNA was diluted 1:5 and subjected to single-cell PCR amplification on a Fluidigm 96.96 Dynamic Array using the Fluidigm BioMark system in combination with EvaGreen chemistry according to the manufacturer's manual ("Fast Gene Expression Analysis Using EvaGreen on the BioMark or BioMark HD System"). Each primer pair was run in technical duplicates on the chip. Only single cells with consistent amplification results between the technical primer replicates were considered positive to minimize false positive calls. Overall discrepancy rate was low (< 3% per primer pair). Expression data was analyzed using the Fluidigm Real-Time PCR analysis software in combination with the Fluidigm SINGuLAR Analysis Toolset for R (Version 3.0.2 (2013-09-25) "Frisbee Sailing").

Microscopy, Antibodies and Flow Cytometry

After washing the cells once with PBS, cells were fixed with 4% (v/v) paraformaldehyde for 20 min, washed twice with PBS, permeabilized using 0.1% (v/v) Triton X-100 in PBS, Cells were blocked with 10% (v/v) FCS in PBS for 1-5h at room temperature. Cells were incubated with primary antibodies diluted in 2% FCS (v/v) in PBS at 4°C overnight. A list of the primary antibodies used in this study is provided as **Table S2**. After primary antibody incubation cells were washed twice with PBS followed by incubation with appropriate AlexaFluor-conjugated secondary antibodies diluted in PBS at room temperature for 1h (1:1000; Molecular Probes, Thermo Fisher). After washing twice with PBS, nuclei were stained using DAPI. After an additional 2 washing steps, fluorescence images of the cells were acquired using an Olympus IX71 inverted microscope equipped with a Hamamatsu ORCA CCD camera. For immunohistochemical analysis, the animals were perfused with PBS and then 4% paraformaldehyde. Matrigel plugs were post-fixed in 4% paraformaldehyde and subsequently immersed in 30% sucrose. Matrigel plugs were cryosectioned at 30 µm for immunohistochemical analysis. The sections were pretreated with Antigen Retrieval Reagent-Universal solution (R&D systems). The sections were washed with PBS and then blocked with blocking solution (1%BSA-0.3%Triton-PBS) for 1 hour at room temperature. The sections were stained with hNA, Ki67, ACTH, GH, TSH, PRL, FSH and LH and subsequently with an Alexa-568 conjugated secondary antibody. The images were acquired using an Olympus BX51 Microscope equipped with a Hamamatsu camera. Stereological quantification of the number of ACTH cells in the whole matrigel plug was conducted using the optical fractionator probe, and the graft volume was analyzed using the Cavalieri estimator method. (Stereo Investigator Software, Microbrightfield Bioscience). For flow analysis cells (different reporter cell lines) were detached from cell culture plastic using TrypLE (Fisher Scientific). After washing once with PBS cells were resuspended in 2% FCS, 1 mM EDTA in PBS and DAPI. Cells were filtered using a 40 µm cell strainer and analyzed on BD LSRFortessa Flow Cytometer. Only single (doublet exclusion) live (DAPI-) cells were analyzed. Data was further processed using FlowJo Version 9.7.6 (FLOWJO LLC).

Cell Surface Marker Screen
For the BD Lyoplate™ cell surface marker screen day 30 cells were replated at a density of 100 000 cells/cm^2 into 96 well imaging plates using Accutase. After a 4 hour attachment phase cells were stained according to the user's manual for bioimaging. Cells were analyzed on an Operetta High Content Imaging System (Perkin Elmer). Images were processed and analyzed using the Harmony Software package (Perkin Elmer).

Stimulation of hormone release
To stimulate hormone release *in vitro*, cells were differentiated in 24 well plates as described above. On day 30 of differentiation cells were washed once with PBS and 250 µl of fresh medium containing either the solvent or the stimulant were added to each well. After 12 h the supernatant was removed and centrifuged for 5 min at 2000g to pellet debris. Supernatant was transferred into fresh reaction tubes, flash frozen and stored at -80ºC until ELISA measurements. Stimulants used were: CRF (Tocris, 1 µM), Stressin I (Tocris, 2 µM), Ghrelin (Tocris, 1 µM), Somatocrinin (Accurate Chemical, 1 µg/ml), Nafarelin (Tocris, 1 µM) and Urocortin (Tocris, 500 nM).

ELISA measurements
Hormone concentration in the supernatant of cells or in animal plasma was analyzed using ELISA measurements. Hormone concentration in the cell culture supernatant was assessed using traditional single hormone ELISA Kits according to the manufacturer's manual (ACTH (Calbiotech, detects rat and human ACTH)), hGH (R&D Systems, human specific), FSH (Calbiotech, FSH (lumELISA, human specific) and corticosterone (Abcam)). Plates were read using an EnSpire Multimode plate reader (PerkinElmer). Hormone concentration for *in vivo* samples was analyzed using either traditional ELISA (for ACTH only, serum diluted 1:2) or species specific (human or rat) Milliplex multiplex ELISA using Luminex technology (Millipore). Magnetic bead-based sandwich immunoassay was performed according to the manufacturer's manual. 25 µl of undiluted serum samples in duplicate wells were analyzed by Luminex FlexMap 3D (Luminex Corp, Austin, TX). Cytokine concentrations were determined by Luminex Xponent 4.1 and EMD-Millipore Milliplex Analyst v5.1 using 5-p log analysis.

Animal maintenance
Male Athymic nude rats (RNU rat Crl:NIH-Foxn1rnu, Charles River Laboratories) were hypophysectomized using the parapharyngeal approach at the age of 8 weeks by Charles Rivers Laboratories. Plasma ACTH was measured 1 week after hypophysectomy to confirm hypopituitarism. The hypophysectomized rats were randomized into two groups: Sham control (n=4), Human ES derived pituitary cells subcutaneously grafted group (n=7). Two million human ES-derived pituitary cells were suspended in matrigel (BD Biosciences) and injected subcutaneously. Blood was taken by retro orbital bleeding before graft, 1 week, 3 weeks, 5 weeks and 7 weeks after the transplantation under isoflurane anesthesia at 8 a.m. Blood was collected with K2 EDTA-treated BD Microtainer MAP (BD Biosciences) and plasma was isolated and stored at -80ºC. All animal procedures were done in accordance with protocols approved by Animal Care and Use Committee at MSKCC and following NIH guidelines.

Statistical analysis
Data are presented as sample means ± SEM, as indicated in each figure legend. Means represent the data of independent experiments (number indicated in the corresponding figure legend). Differences between groups were analyzed by unpaired t tests or one-way ANOVA with Bonferroni multiple-comparison post hoc test. $p < 0.05$ = *, $p < 0.01$ = **, $p < 0.001$ = ***, $p < 0.0001$ = ****

Supplemental References

Chambers, S.M., Fasano, C.A., Papapetrou, E.P., Tomishima, M., Sadelain, M., and Studer, L. (2009). Highly efficient neural conversion of human ES and iPS cells by dual inhibition of SMAD signaling. Nature biotechnology *27*, 275-280.
Chen, G. (2008). Splitting hESC/hiPSC lines with EDTA in feeder free conditions. In StemBook (Cambridge (MA)).

Chen, G., Gulbranson, D.R., Hou, Z., Bolin, J.M., Ruotti, V., Probasco, M.D., Smuga-Otto, K., Howden, S.E., Diol, N.R., Propson, N.E., *et al.* (2011). Chemically defined conditions for human iPSC derivation and culture. Nat Methods *8*, 424-429.

Livak, K.J., and Schmittgen, T.D. (2001). Analysis of relative gene expression data using real-time quantitative PCR and the 2(-Delta Delta C(T)) Method. Methods *25*, 402-408.

Maroof, A.M., Keros, S., Tyson, J.A., Ying, S.W., Ganat, Y.M., Merkle, F.T., Liu, B., Goulburn, A., Stanley, E.G., Elefanty, A.G., *et al.* (2013). Directed differentiation and functional maturation of cortical interneurons from human embryonic stem cells. Cell stem cell *12*, 559-572.

Merkle, F.T., Maroof, A., Wataya, T., Sasai, Y., Studer, L., Eggan, K., and Schier, A.F. (2015). Generation of neuropeptidergic hypothalamic neurons from human pluripotent stem cells. Development *142*, 633-643.
